# Design and synthesis of novel anti-urease imidazothiazole derivatives with promising antibacterial activity against *Helicobacter pylori*

**DOI:** 10.1371/journal.pone.0286684

**Published:** 2023-06-02

**Authors:** Afnan I. Shahin, Sumera Zaib, Seyed-Omar Zaraei, Reena A. Kedia, Hanan S. Anbar, Muhammad Tayyab Younas, Taleb H. Al-Tel, Ghalia Khoder, Mohammed I. El-Gamal

**Affiliations:** 1 Research Institute for Medical and Health Sciences, University of Sharjah, Sharjah, United Arab Emirates; 2 Faculty of Science and Technology, Department of Basic and Applied Chemistry, University of Central Punjab, Lahore, Pakistan; 3 Department of Clinical Pharmacy and Pharmacotherapeutics, Dubai Pharmacy College for Girls, Dubai, United Arab Emirates; 4 Department of Medicinal Chemistry, College of Pharmacy, University of Sharjah, Sharjah, United Arab Emirates; 5 Department of Pharmaceutics and Pharmaceutical Technology, College of Pharmacy, University of Sharjah, Sharjah, United Arab Emirates; 6 Faculty of Pharmacy, Department of Medicinal Chemistry, Mansoura University, Mansoura, Egypt; Helwan University, EGYPT

## Abstract

Urease enzyme is a known therapeutic drug target for treatment of *Helicobacter pylori* infection due to its role in settlement and growth in gastric mucosa. In this study, we designed a new series of sulfonates and sulfamates bearing imidazo[2,1-*b*]thiazole scaffold that exhibit a potent inhibitory activity of urease enzyme. The most potent compound **2c** inhibited urease with an IC_50_ value of 2.94 ± 0.05 μM, which is 8-fold more potent than the thiourea positive control (IC_50_ = 22.3 ± 0.031 μM). Enzyme kinetics study showed that compound **2c** is a competitive inhibitor of urease. Molecular modeling studies of the most potent inhibitors in the urease active site suggested multiple binding interactions with different amino acid residues. Phenotypic screening of the developed compounds against *H*. *pylori* delivered molecules of that possess high potency (**1a, 1d, 1h, 2d**, and **2f**) in comparison to the positive control, acetohydroxamic acid. Additional studies to investigate the selectivity of these compounds against AGS gastric cell line and *E*. *coli* were performed. Permeability of the most promising derivatives (**1a, 1d, 1h, 2d**, and **2f)** in Caco-2 cell line, was investigated. As a result, compound **1d** presented itself as a lead drug candidate since it exhibited a promising inhibition against urease with an IC_50_ of 3.09 ± 0.07 μM, MIC value against *H*. *pylori* of 0.031 ± 0.011 mM, and SI against AGS of 6.05. Interestingly, compound **1d** did not show activity against urease-negative *E*. *coli* and exhibited a low permeability in Caco-2 cells which supports the potential use of this compound for GIT infection without systemic effect.

## 1. Introduction

*Helicobacter pylori (H*. *pylori)* has been classified by the World Health Organization (WHO) as a group 1 carcinogen [1]. The role of *H*. *pylori* in gastric cancer was reinforced by the calculation of its attributable factor (AF), which was found to be 89.0%, posing a significant global burden [2]. Infecting approximately 50% of the global population, the *H*. *pylori* infection’s prevalence is of a great concern [3, 4]. Having a determinant pathogenic role in non-cardia gastric cancer, gastritis, peptic ulcer disease and gastric mucosa associated lymphoid tissue (MALT) lymphoma, it was also reported to have several extra gastric systemic manifestations. Additionally, Alzheimer’s disease, Parkinson’s disease, iron deficiency anemia, primary immune thrombocytopenia, coronary atherosclerotic disease, and diabetes mellitus have been reported to have strong association with *H*. *pylori* infection [5].

The WHO has included *H*. *pylori* within the list of 16 antibiotic-resistant bacteria posing major global health threats. This is in response to the increasing rate of antibiotic resistance complicating treatment strategies, which is faster than the rate of introducing new antibiotics [6]. *H*. *pylori* can survive the hostile pH conditions due to its abundant secretion of urease, and despite not being acidophil, it can reach and colonize the gastric mucosa in humans. Therefore, inhibiting the urease activity seems to be an important therapeutic target. Therefore, it is highly recommended to consider *H*. *pylori* as a high priority in terms of the need to develop novel agents to combat it [7–12].

Urease is a nickel-containing hetero-polymeric enzyme belonging to amidohydrolase and phosphotriesterase family which catalyzes the conversion of urea into ammonia and carbamate in living organism at almost 10^14^ times faster than non-catalyzed reactions [13, 14]. Hydrogen bonding enables the urease to make a weak interaction with urea to liberate the carbamate group, which delivers ammonia and carbonic acid [1]. Ureases are abundantly found in organisms such as prokaryotic bacteria, eukaryotic organism such as algae, fungi and plants [2]. Urease acts as a hostile factor in various ureolytic bacteria and plays a vital role in the pathogenesis of nephrolithiasis, chronic and acute pyelonephritis, gastric ulcer and some other diseases. Ureases are also indicated in the development of urolithiasis, portosystemic encephalopathy, and catheter encrustation [3].

Therefore, urease has become an important target for development of anti-Helicobacter drug candidates. *H*. *pylori* (microaerophilic bacteria) having effective urease activity is the detrimental bacteria in the digestive tract of human and was found to colonize >50% of human population. It also facilitates the organism to survive in acidic conditions by forming a layer around its surface by the ammonia production [4]. Consequently, inhibition of urease enzyme might be a promising action for avoiding detrimental effects of ureolytic infections in humans [6].

**Fig 1:**
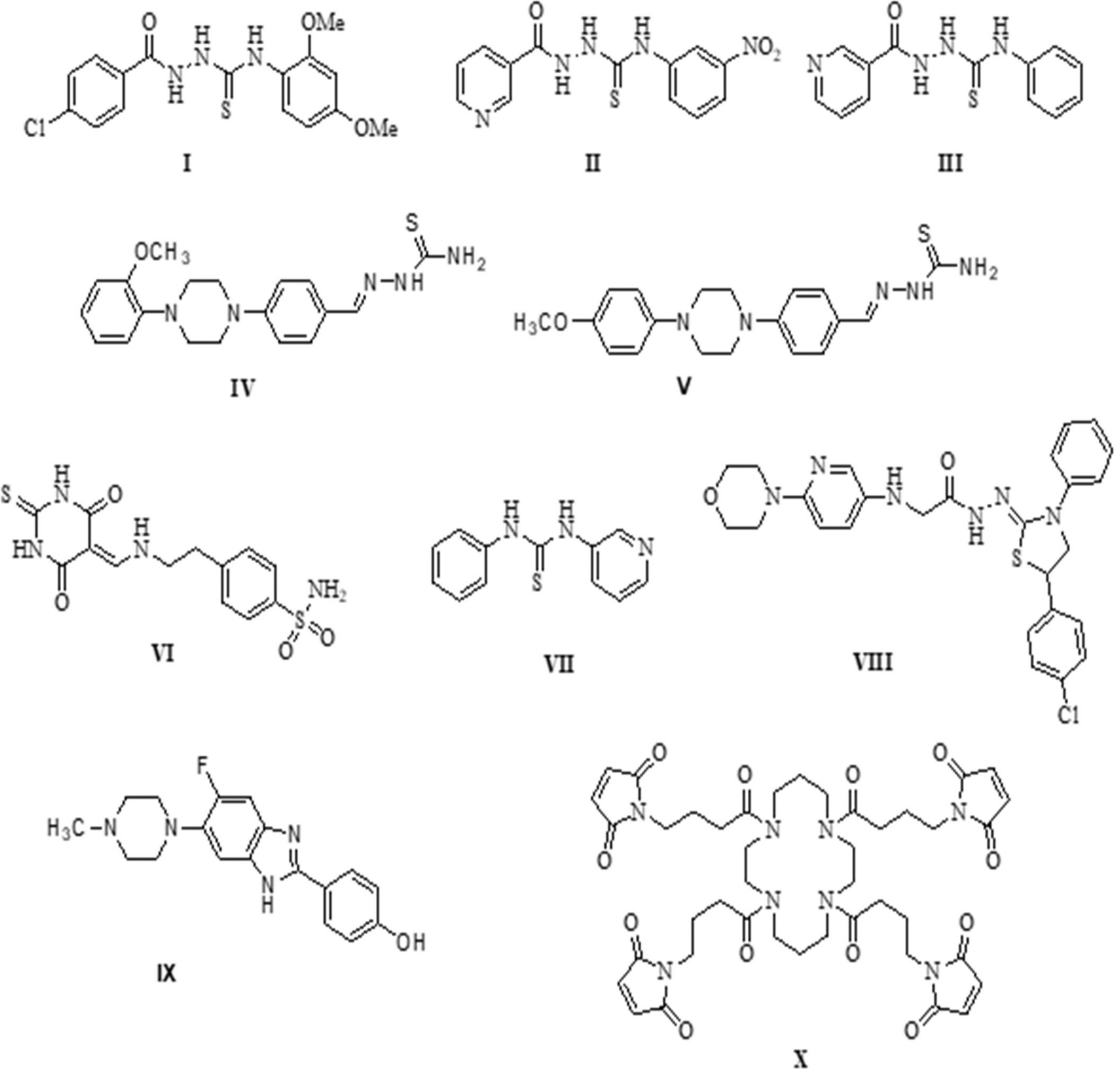
Depiction of several urease inhibitors.

Several potent urease inhibitors have been described (Fig 1). These molecules contain different chemical moieties such as thiosemicarbazides **I, II, III**, **IV,** and **V** [15–18], barbituric acid derivatives **VI** [19, 20], thiourea **VII** [21], morpholines **VIII** [22], benzimidazole derivatives **IX** [23] and nociceptin/orphanin derivatives **X** [24]. It is interesting to note that different reports examined the significance of H-bonding, metal-chelating and other moieties as important pharmacophoric characteristics for the inhibitors’ potency of urease enzyme [25–28]. All the reported urease inhibitors have a promising role in the treatment of various bacterial infections as well as other diseases. Urease inhibitors have been employed as antifungal, anti-diabetics, anti-oxidant, anti-inflammatory, anti-convulsant [29], anti-hypertensive, anti-cancer [30], anti-viral [31, 32] anti-tuberculosis, and anti-bacterial [33, 34].

Heterocyclic scaffolds are of a great interest to medicinal chemists to be utilized in constructing a pilot library of compounds, and thus expands the drug-like chemical space, resulting in the development of novel and active compounds pharmacologically [35, 36]. Numerous heterocyclic systems were reported in more than 85% of FDA approved drugs and hence they were witnessed as privilege scaffolds. [35, 36] It has been reported that the most heteroatoms present in numerous drugs are, nitrogen [37], sulfur [38], and oxygen [39]. Interestingly, the selection of heterocycles can offer a tool in optimizing the physicochemical properties or ADME properties due to variations in solubility, lipophilicity, polarity, and hydrogen bonding capacity among various heterocycles [35]. Statistically, 59% of the FDA approved drugs possess a nitrogen heterocycle, and 40% of those utilizing nitrogen heterocycles also contained a sulfur atom [40]. Hence, we aimed to shed the light on the potential role of scaffolds having both nitrogen and sulfur atoms, such as imidazothiazoles.

**Fig 2:**
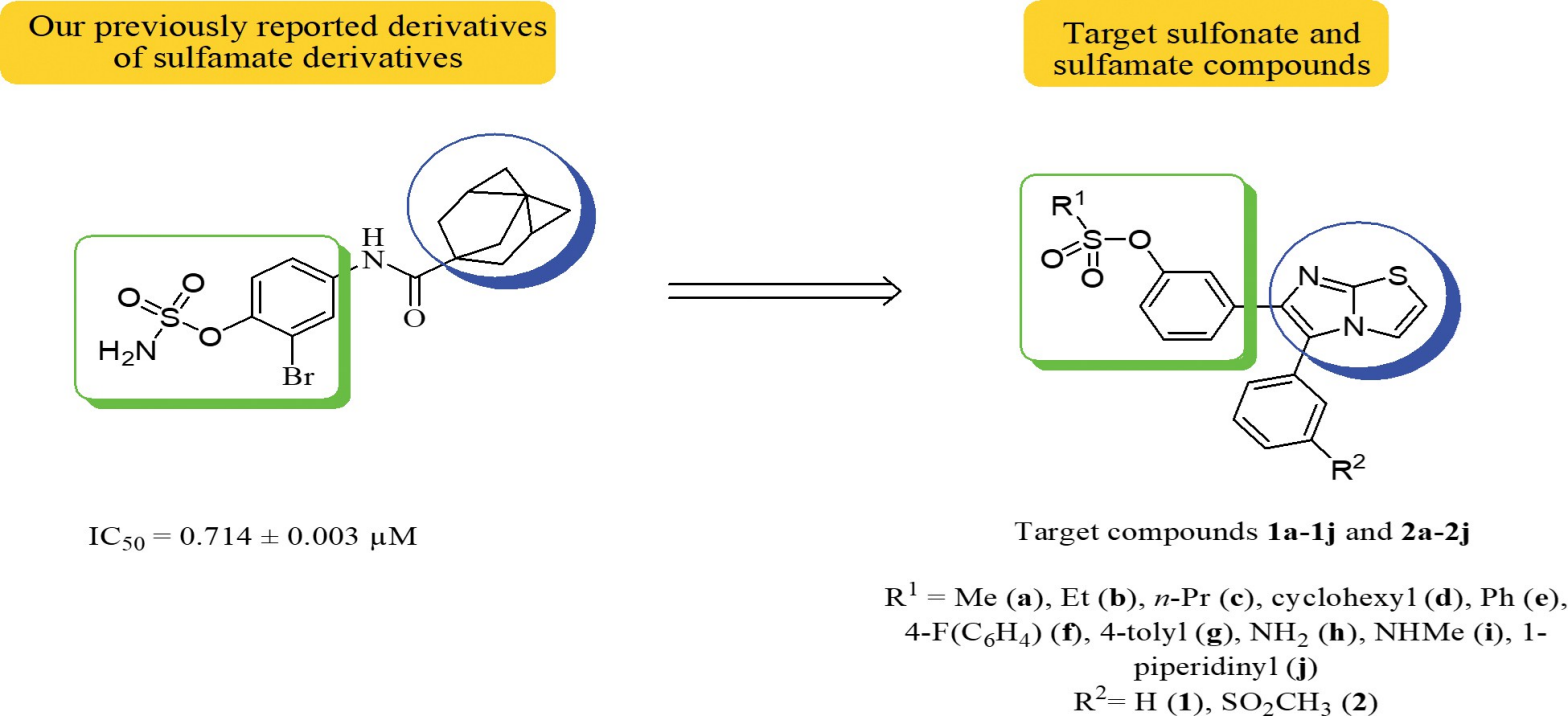
Previously synthesized compounds and target compounds as urease inhibitors.

In parallel, sulfonates and sulfamates have been endorsed as multifaceted and medicinally compatible moieties that have gained an immense significance by virtue of their value in medicinal compound libraries [41]. Sulfamate derivatives have been recently explored as Jack bean urease inhibitors (Fig 2) [42]. This led us to develop a new series of sulfonate and sulfamate derivatives as potential inhibitors of urease. The core scaffold of the developed series is the 5,6-diphenylimidazole[2,1-*b*]thiazole anchored with sulfonate and sulfamate moieties on the phenyl ring resident on the imidazole ring. To assess the activity of the developed compounds, various methods were utilized including enzymatic assay, antibacterial studies, structure-activity relationship (SAR), molecular modeling, and ADME analysis.

## 2. Materials and methods

### 2.1. General

Commercially available solvent and reagents were utilized with no additional purification. Analytical thin-layer chromatography (TLC) from Merck was adopting on silica gel 60 F254 plates. Column chromatography was often used to purify target compounds, using silica gel (0.040–0.063 mm, 230–400 mesh) and technical grade solvents. The determination of purity percentages was assessed by LC-MS and were confirmed to be more than 96%. Stuart melting point apparatus (Staffordshire, UK) was utilized to measure the melting points and are uncorrected. ^1^H NMR and ^13^C NMR spectra were recorded on a Bruker Avance 500 MHz spectrometer.

Jackbean urease kit, thiourea, urea, K_2_HPO_4_, LiCl_2_ and EDTA were purchased from Sigma Aldrich. Bacterial strains (*H*. *pylori* and *E*. *coli*) and the AGS cell line were purchased from the German collection of microorganisms and cell cultures, GmbH (DSMZ; Germany) and the German Cell Lines Service (CLS, Germany), respectively. Bacterial and cell culture media (Brucella agar, RPMI, FBS and horse serum) and Acetohydroxamic acid (AHA) were all purchased from Sigma, USA. To ensure microaerophilic culture of *H*. *pylori*, CampyGen 2.5 L pack (Oxoid, thermo scientific, UK) and anaerobic jar (Biomerieux, France) were used. The bacterial density was adjusted using a DensiCHEK PLUS device (Biomerieux, France). The optical density was determined using a Synergy H1 microplate reader (BioTek, USA) at a wavelength of 570–600 nm. The IC_50_ was calculated using GraphPad Prism 8.0.2 for windows (GraphPad Inc., La Jolla, CA, USA). For MIC, the agar dilution method was performed as per the CLSI guidelines.

### 2.2. Synthesis of the target compounds

#### 2.2.1. Synthesis of the intermediate compounds 6 and 9

They were successfully synthesized following the procedure reported in the literature [43].

#### 2.2.2. General procedure for synthesis of sulfonate derivatives 1a-1g and 2a-2g

The 3-(5-phenylimidazo[2,1-*b*]thiazol-6-yl)phenol (**6**, 34 mg, 0.117 mmol) or 3-(5-3-(methylsulfonyl)phenyl)imidazo[2,1-*b*]thiazol-6-yl)phenol (**9**, 40 mg, 0.108 mmol) was dissolved in anhydrous DMF (1 mL) at 0°C, triethylamine (0.506 g, 697 μL, 5 mmol) was then added at the same temperature. An appropriate sulfonyl chloride reagent (0.54–1.62 mmol) was diluted in anhydrous DMF (0.5 mL), this solution was added dropwise to the reaction mixture at 0°C. After 5 min, the reaction was stirred at room temperature for 1–4 h under nitrogen atmosphere. After confirming the reaction completion using TLC, the reaction mixture was extracted using brine (20 mL) and ethyl acetate (3 × 20 mL). The organic layer was collected and dried by anhydrous Na_2_SO_4_, and then evaporated to dryness. The final compound was then purified by normal phase column chromatography, using the mobile phase: hexane and ethyl acetate with an appropriate ratio.

#### 2.2.3. 3-(5-Phenylimidazo[2,1*-b*]thiazol-6-yl)phenyl methanesulfonate (1a)

Purified by flash column chromatography (silica gel, hexane:ethyl acetate 75%:25% v/v then switching to hexane:ethyl acetate 72%:28% v/v); yellow solid; yield: 20%; mp: 105–108°C; ^1^H NMR (CDCl_3_, 500 MHz) δ 7.58 (d, 1H, *J* = 7.5 Hz), 7.51–7.47 (m, 6H), 7.40 (s, 1H), 7.34 (t, 1H, *J* = 8.0 Hz), 7.20 (d, 1H, *J* = 8.0 Hz), 6.96 (s, 1H) [Ar-H], 3.06 (s, 3H, OSO_2_-CH_3_); ^13^C NMR (CDCl_3_, 125 MHz) δ 149.7, 148.9, 140.1, 135.2, 130.3, 129.8, 129.6, 129.5, 129.1, 126.5, 123.8, 121.7, 120.9, 117.9, 114.6 [Ar-C], 37.7 (OSO_2_-CH_3_); LC-MS: 371.18 [M + 1]^+^.

#### 2.2.4. 3-(5-Phenylimidazo[2,1*-b*]thiazol-6-yl)phenyl ethanesulfonate (1b)

Purified by flash column chromatography (silica gel, hexane:ethyl acetate 80%:20% v/v then switching to hexane:ethyl acetate 77%:23% v/v); brown solid; yield: 54%; mp: 119–121°C; ^1^H NMR (CDCl_3_, 500 MHz) δ 7.58 (d, 1H, *J* = 7.5 Hz), 7.56–7.52 (m, 3H), 7.49–7.47 (m, 4H), 7.37 (t, 1H, *J* = 8.5 Hz), 7.24 (dd, 1H, *J* = 1.5, 8.5 Hz), 7.13 (d, 1H, J = 2.0 Hz) [Ar-H], 3.24 (q, 2H, *J* = 7.5 Hz, OSO_2_-CH_2_), 1.46 (t, 3H, *J* = 7.5 Hz, OSO_2_-CH_2_-CH_3_); ^13^C NMR (CDCl_3_, 125 MHz) δ 149.7, 148.2, 135.3, 130.9, 130.6, 130.2, 130.0, 129.6, 129.3, 128.8, 127.9, 126.3, 123.8, 122.6, 120.9, 118.1, 116.1 [Ar-C], 45.3 (OSO_2_-CH_2_), 8.4 (OSO_2_-CH_2_-CH_3_); LC-MS: 385.17 [M + 1]^+^.

#### 2.2.5. 3-(5-Phenylimidazo[2,1*-b*]thiazol-6-yl)phenyl propane-1-sulfonate (1c)

Purified by flash column chromatography (silica gel, hexane:ethyl acetate 95%:5% v/v then switching to hexane:ethyl acetate 92%:8% v/v); brown solid; yield: 40%; mp: 120–123°C; ^1^H NMR (CDCl_3_, 500 MHz) δ 7.61 (d, 1H, *J* = 7.5 Hz), 7.58–7.44 (m, 7H), 7.40 (t, 1H, *J* = 7.5 Hz), 7.27 (s, 1H), 7.19 (s, 1H) [Ar-H], 3.23 (t, 2H, *J* = 8.0 Hz, OSO_2_-CH_2_), 1.96 (q, 2H, *J* = 7.5 Hz, OSO_2_-CH_2_-CH_2_), 1.11 (t, 3H, *J* = 7.5 Hz, OSO_2_-CH_2_-CH_2_-CH_3_); ^13^C NMR (CDCl_3_, 125 MHz) δ 149.5, 149.3, 141.8, 136.7, 130.0, 129.9, 129.6, 129.4, 128.9, 126.1, 123.6, 120.9, 120.6, 117.5, 113.2 [Ar-C], 52.1 (OSO_2_-CH_2_), 17.4 (OSO_2_-CH_2_-CH_2_), 13.0 (OSO_2_-CH_2_-CH_2_-CH_3_); LC-MS: 399.22 [M + 1]^+^.

#### 2.2.6. 3-(5-Phenylimidazo[2,1*-b*]thiazol-6-yl)phenyl cyclohexane-1-sulfonate (1d)

Purified by flash column chromatography (silica gel, hexane:ethyl acetate 80%:20% v/v then switching to hexane:ethyl acetate 75%:25% v/v); pale yellow solid; yield: 75%; mp: 118–120°C; ^1^H NMR (CDCl_3_, 500 MHz) δ 7.56 (d, 1H, *J* = 8.0 Hz), 7.52–7.44 (m, 6H), 7.38 (d, 1H, *J* = 4.5 Hz), 7.31 (t, 1H, *J* = 8.0 Hz), 7.16 (dd, 1H, *J* = 1.5 Hz, 8.0 Hz), 6.90 (d, 1H, *J* = 4.0 Hz) [Ar-H], 3.14–3.09 (m, 1H, OSO_2_-CH), 2.25 (d, 2H, *J* = 13.0 Hz, cyclohexyl CH-CH_2_), 1.90 (d, 2H, *J* = 13.0 Hz, cyclohexyl CH_2_-CH), 1.73–1.61 (m, 3H, cyclohexyl CH_2_-CH_2_-CH-CH_2_-CH_2_), 1.73–1.61 (m, 3H, cyclohexyl CH_2_-CH_2_-CH-CH_2_-CH_2_), 1.43–1.40 (m, 1H, cyclohexyl CH_2_-CH_2_-CH-CH_2_-CH_2_), 1.31–1.27 (m, 2H, cyclohexyl CH-CH_2_-CH_2_-CH_2_); ^13^C NMR (CDCl_3_, 125 MHz) δ 149.4, 149.0, 140.8, 135.6, 130.0, 129.7, 129.5, 129.2, 125.9, 123.6, 121.3, 120.8, 117.7, 113.8 [Ar-C], 60.2 (OSO_2_-CH), 26.6 (cyclohexyl CH_2_-CH-CH_2_), 25.1 (cyclohexyl CH_2_-CH_2_-CH-CH_2_-CH_2_), 22.8 (cyclohexyl CH-CH_2_-CH_2_-CH_2_); LC-MS: 439.10 [M + 1]^+^.

#### 2.2.7. 3-(5-Phenylimidazo[2,1*-b*]thiazol-6-yl)phenyl benzenesulfonate (1e)

Purified by flash column chromatography (silica gel, hexane:ethyl acetate 90%:10% v/v then switching to hexane:ethyl acetate 85%:15% v/v); white solid; yield: 20%; mp: 119–122°C; ^1^H NMR (CDCl_3_, 500 MHz) δ 7.78 (d, 2H, *J* = 7.5 Hz), 7.64 (t, 2H, *J* = 7.0 Hz), 7.51–7.48 (m, 5H), 7.41–7.39 (m, 3H), 7.25–7.23 (m, 2H), 6.95 (s, 1H), 6.85 (d, 1H, *J* = 8.0 Hz) [Ar-H]; ^13^C NMR (CDCl_3_, 125 MHz) δ 149.8, 148.7, 135.6, 134.3, 130.0, 129.8, 129.5, 129.4, 129.3, 128.8, 128.6, 126.4, 123.6, 121.5, 121.2, 117.9, 114.5 [Ar-C]; LC-MS: 433.23 [M + 1]^+^.

#### 2.2.8. 3-(5-Phenylimidazo[2,1*-b*]thiazol-6-yl)phenyl 4-fluorobenzenesulfonate (1f)

Purified by flash column chromatography (silica gel, hexane:ethyl acetate 95%:5% v/v then switching to hexane:ethyl acetate 93%:7% v/v); yellow solid; yield: 48%; mp: 109–111°C; ^1^H NMR (CDCl_3_, 500 MHz) δ 7.80–7.77 (m, 2H), 7.65 (d, 1H, *J* = 8.0 Hz), 7.54–7.49 (m, 3H), 7.42–7.39 (m, 3H), 7.28–7.27 (m, 1H), 7.20–7.14 (m, 3H), 6.98 (d, 1H, *J* = 4.5 Hz), 6.90 (dd, 1H, *J* = 1.5 Hz, 8.0 Hz) [Ar-H]; ^13^C NMR (CDCl_3_, 125 MHz) δ 167.1, 165.1, 149.7, 148.6, 131.4 d, *J* = 9.5 Hz, C-F coupling), 130.2, 129.8, 129.6, 129.4, 126.5, 123.7, 121.6, 121.0, 117.9, 116.7 (d, *J* = 22.6 Hz, C-F coupling) [Ar-C]; LC-MS: 451.21 [M + 1]^+^.

#### 2.2.9. 3-(5-Phenylimidazo[2,1*-b*]thiazol-6-yl)phenyl 4-methylbenzenesulfonate (1g)

Purified by flash column chromatography (silica gel, hexane:ethyl acetate 90%:10% v/v then switching to hexane:ethyl acetate 87%:13% v/v); white solid; yield: 48%; mp: 103–106°C; ^1^H NMR (CDCl_3_, 500 MHz) δ 7.65–7.62 (m, 3H), 7.52–7.49 (m, 3H), 7.42–7.39 (m, 3H), 7.27 (d, 2H, *J* = 8.0 Hz), 7.23 (t, 2H, *J* = 8.0 Hz), 6.97 (d, 1H, *J* = 4.0 Hz), 6.84 (dd, 1H, *J* = 2.0 Hz, 8.5 Hz) [Ar-H], 2.43 (s, 3H, Ph-CH_3_); ^13^C NMR (CDCl_3_, 125 MHz) δ 149.8, 148.6, 145.4, 132.5, 130.0, 129.9, 129.8, 129.5, 129.4, 128.8, 128.6, 126.3, 123.6, 121.6, 121.2, 117.9, 114.6 [Ar-C], 21.9 (Ph-CH_3_); LC-MS: 447.11 [M + 1]^+^.

#### 2.2.10. 3-(5-(3-(Methylsulfonyl)phenyl)imidazo[2,1*-b*]thiazol-6-yl)phenyl methanesulfonate (2a)

Purified by flash column chromatography (silica gel, hexane:ethyl acetate 66%:34% v/v then switching to hexane:ethyl acetate 64%:36% v/v); white solid; yield: 32%; mp: 159–160°C; ^1^H NMR (CDCl_3_, 500 MHz) δ 8.03–8.00 (m, 2H), 7.72 (t, 2H, *J* = 8.0 Hz), 7.62 (d, 1H, *J* = 7.0 Hz), 7.42–7.37 (m, 3H), 7.19 (d, 1H, *J* = 8.0 Hz), 6.98 (s, 1H) [Ar-H], 3.09 (s, 6H, 2SO_2_-CH_3_); ^13^C NMR (CDCl_3_, 125 MHz) δ 150.1, 149.5, 142.3, 135.6, 134.4, 131.2, 130.9, 130.5, 127.9, 127.6, 126.7, 121.5, 121.4, 121.1, 117.4, 114.6 [Ar-C], 44.6 (OSO_2_-CH_3_), 37.6 (SO_2_-CH_3_); LC-MS: 449.13 [M + 1]^+^.

#### 2.2.11. 3-(5-(3-(Methylsulfonyl)phenyl)imidazo[2,1*-b*]thiazol-6-yl)phenyl ethanesulfonate (2b)

Purified by flash column chromatography (silica gel, hexane:ethyl acetate 60%:40% v/v then switching to hexane:ethyl acetate 55%:45% v/v); brown solid; yield: 44%; mp: 206–209°C; ^1^H NMR (DMSO-*d*_*6*_, 500 MHz) δ 8.02–8.00 (m, 2H), 7.87–7.85 (m, 1H), 7.83–7.78 (m, 2H), 7.48–7.46 (m, 1H), 7.44–7.38 (m, 3H), 7.23–7.21 (m, 1H) [Ar-H], 3.43 (d, 2H, *J* = 7.5 Hz, OSO_2_-CH_2_), 3.25 (s, 3H, SO_2_-CH_3_), 1.28 (t, 3H, *J* = 7.5 Hz, OSO_2_-CH_2_-CH_3_); ^13^C NMR (DMSO-*d*_*6*_, 125 MHz) δ 149.4, 149.1, 141.9, 141.6, 136.1, 134.3, 130.8, 130.6, 130.4, 127.5, 127.0, 125.6, 121.6, 121.0, 120.4, 118.6, 114.8 [Ar-C], 44.5 (OSO_2_-CH_2_), 43.5 (SO_2_-CH_2_), 8.0 (OSO_2_-CH_2_-CH_3_); LC-MS: 463.12 [M + 1]^+^.

#### 2.2.12. 3-(5-(3-(Methylsulfonyl)phenyl)imidazo[2,1*-b*]thiazol-6-yl)phenyl propane-1-sulfonate (2c)

Purified by flash column chromatography (silica gel, hexane:ethyl acetate 70%:30% v/v then switching to hexane:ethyl acetate 65%:35% v/v); brown solid; yield: 80%; mp: 188–191°C; ^1^H NMR (CDCl_3_, 500 MHz) δ 8.03–7.99 (m, 2H), 7.74–7.64 (m, 2H), 7.60 (d, 1H, *J* = 8.0 Hz), 7.41–7.35 (m, 3H), 7.15 (dd, 1H, *J* = 1.5 Hz, 8.0 Hz), 6.94 (d, 1H, *J* = 4.5 Hz) [Ar-H], 3.18–3.15 (m, 2H, OSO_2_-CH_2_), 3.10 (s, 3H_,_ SO_2_-CH_3_), 1.99–1.92 (m, 2H, OSO_2_-CH_2_-CH_2_), 1.09 (t, 3H, *J* = 7.5 Hz, OSO_2_-CH_2_-CH_2_-CH_3_); ^13^C NMR (CDCl_3_, 125 MHz) δ 150.3, 149.4, 143.1, 142.2, 136.1, 134.4, 131.6, 130.8, 130.3, 127.7, 127.4, 126.5, 121.5, 121.1, 117.3, 114.1 [Ar-C], 52.3 (OSO_2_-CH_2_), 44.5 (SO_2_-CH_3_), 17.4 (OSO_2_-CH_2_-CH_2_), 13.0 (OSO_2_-CH_2_-CH_2_-CH_3_); LC-MS: 477.17 [M + 1]^+^.

#### 2.2.13. 3-(5-(3-(Methylsulfonyl)phenyl)imidazo[2,1*-b*]thiazol-6-yl)phenyl cyclohexane-1-sulfonate (2d)

Purified by flash column chromatography (silica gel, hexane:ethyl acetate 70%:30% v/v then switching to hexane:ethyl acetate 65%:35% v/v); white solid; yield: 16%; mp: 142–144°C; ^1^H NMR (CDCl_3_, 500 MHz) δ 8.06–8.05 (m, 2H), 7.76–7.72 (m, 2H), 7.69 (d, 1H, *J* = 8.0 Hz), 7.48 (d, 1H, *J* = 4.0 Hz), 7.43 (t, 1H, *J* = 8.0 Hz), 7.33 (s, 1H), 7.18 (dd, 1H, *J* = 1.5 Hz, 8.0 Hz), 7.14 (d, 1H, *J* = 3.0 Hz) [Ar-H]; 3.17–3.14 (m, 1H, OSO_2_-CH), 3.11 (s, 3H, SO_2_-CH_3_), 2.25 (d, 2H, *J* = 12.0 Hz, cyclohexyl CH-CH_2_), 1.94–1.91 (m, 2H, cyclohexyl CH_2_-CH), 1.75–1.63 (m, 4H, cyclohexyl CH_2_-CH_2_-CH-CH_2_-CH_2_), 1.35–1.31 (m, 2H, cyclohexyl CH-CH_2_-CH_2_-CH_2_), ^13^C NMR (CDCl_3_, 125 MHz) δ 150.3, 149.3, 143.2, 142.2, 136.0, 134.5, 131.6, 130.8, 130.2, 127.8, 127.4, 126.3, 121.5, 121.3, 121.1, 117.4, 114.1 [Ar-C], 60.3 (OSO_2_-CH), 44.7 (cyclohexyl CH_2_-CH-CH_2_), 26.6 (cyclohexyl CH_2_-CH_2_-CH-CH_2_-CH_2_), 25.1(cyclohexyl CH-CH_2_-CH_2_-CH_2_); LC-MS: 517.24 [M + 1]^+^.

#### 2.2.14. 3-(5-(3-(Methylsulfonyl)phenyl)imidazo[2,1*-b*]thiazol-6-yl)phenyl benzenesulfonate (2e)

Purified by flash column chromatography (silica gel, hexane:ethyl acetate 75%:25% v/v then switching to hexane:ethyl acetate 72%:28% v/v); off-white solid; yield: 33%; mp: 193–195°C; ^1^H NMR (CDCl_3_, 500 MHz) δ 8.01 (s, 2H), 7.78 (d, 2H, *J* = 8.0 Hz), 7.72 (s, 2H), 7.65 (q, 2H, *J* = 7.5 Hz), 7.51 (t, 2H, *J* = 8.0 Hz), 7.41 (s, 1H), 7.25–7.23 (m, 2H), 6.96 (s, 1H), 6.71 (d, 1H, *J* = 7.5 Hz) [Ar-H], 3.11 (s, 3H, SO_2_-CH_3_); ^13^C NMR (CDCl_3_, 125 MHz) δ 150.1, 149.7, 142.3, 135.3, 134.5, 134.3, 131.2, 130.9, 130.1, 129.4, 128.5, 127.9, 127.6, 126.7, 121.6, 121.4, 121.3, 117.4, 114.5 [Ar-C], 44.6 (SO_2_-CH_3_); LC-MS: 511.19 [M + 1]^+^.

#### 2.2.15. 3-(5-(3-(Methylsulfonyl)phenyl)imidazo[2,1*-b*]thiazol-6-yl)phenyl 4-fluorobenzenesulfonate (2f)

Purified by flash column chromatography (silica gel, hexane:ethyl acetate 70%:30% v/v then switching to hexane:ethyl acetate 65%:35% v/v); white solid; yield: 44%; mp: 103–105°C; ^1^H NMR (CDCl_3_, 500 MHz) δ 8.02–8.00 (m, 2H), 7.82–7.79 (m, 2H), 7.73–7.72 (m, 2H), 7.61 (d, 1H, *J* = 7.5 Hz), 7.41 (d, 1H, *J* = 4.5 Hz), 7.25–7.18 (m, 4H), 6.97 (d, 1H, *J* = 4.5 Hz), 6.74 (dd, 1H, *J* = 1.5 Hz, 8.0 Hz) [Ar-H], 3.11 (s, 3H, SO_2_-CH_3_); ^13^C NMR (CDCl_3_, 125 MHz) δ 167.2, 165.2, 150.0, 149.6, 142.3, 142.2, 135.3, 134.2, 131.5 (d, *J* = 9.8 Hz, C-F coupling), 131.3, 131.3, 131.2, 130.9, 130.1, 127.8 (d, *J* = 30.9 Hz, C-F coupling), 126.8, 121.6, 121.5, 121.4, 117.4, 116.8 (d, *J* = 22.8 Hz, C-F coupling), 114.6 [Ar-C], 44.5 (SO_2_-CH_3_); LC-MS: 529.03 [M + 1]^+^.

#### 2.2.16. 3-(5-(3-(Methylsulfonyl)phenyl)imidazo[2,1*-b*]thiazol-6-yl)phenyl 4-methylbenzenesulfonate (2g)

Purified by flash column chromatography (silica gel, hexane:ethyl acetate 70%:30% v/v then switching to hexane:ethyl acetate 65%:35% v/v); white solid; yield: 17%; mp: 172–174°C; ^1^H NMR (CDCl_3_, 500 MHz) δ 8.02 (s, 2H), 7.73 (s, 2H), 7.65 (d, 3H, *J* = 7.5 Hz), 7.41 (s, 1H), 7.30 (d, 2H, *J* = 7.5 Hz), 7.24 (s, 2H), 6.97 (s, 1H), 6.70 (d, 1H, *J* = 8.0 Hz) [Ar-H], 3.12 (s, 3H, SO_2_-CH_3_), 2.44 (s, 3H, Ph-CH_3_,); ^13^C NMR (CDCl_3_, 125 MHz) δ 149.7, 149.7, 145.7, 142.5, 134.5, 134.0, 133.9, 132.3, 131.1, 130.5, 130.3, 130.0, 128.6, 128.1, 128.1, 126.8, 121.8, 121.7, 121.5, 115.6 [Ar-C], 44.7 (SO_2_-CH_3_), 21.9 (Ph-CH_3_); LC-MS: 525.12 [M + 1]^+^.

#### 2.2.17. General procedure for synthesis of sulfamate derivatives 1h-1j and 2h-2j

The hydroxyl intermediate **5** (34 mg, 0.117 mmol) or **8** (40 mg, 0.108 mmol) was dissolved in anhydrous DMAc (1–2 M) at 0°C. At the same temperature, an appropriate sulfamoyl chloride reagent (1.62 mmol) was added dropwise to the reaction mixture. In case of compounds **1j** and **2j**, sodium hydride 60% dispersed in mineral oil (4.7 mg, 0.117 mmol) was added in case of compounds **1j** or **2j** before adding piperidine-1-sulfonyl chloride. After 5 min, the reaction was stirred at room temperature overnight under nitrogen atmosphere until reaction completion was confirmed by TLC. The reaction mixture was extracted using brine (20 mL) and ethyl acetate (3×20 mL). The organic layer was collected and dried by anhydrous Na_2_SO_4_, and then evaporated to dryness. The final compound was then purified by normal phase column chromatography, using the mobile phase: hexane and ethyl acetate with an appropriate ratio.

#### 2.2.18. 3-(5-Phenylimidazo[2,1*-b*]thiazol-6-yl)phenyl sulfamate (1h)

Purified by flash column chromatography (silica gel, hexane:ethyl acetate 80%:20% v/v then switching to hexane:ethyl acetate 75%:25% v/v); white crystal; yield: 46%; mp: 173–175°C; ^1^H NMR (DMSO-*d*_*6*_, 500 MHz) δ 7.98 (brs, 2H, NH_2_), 7.73 (d, 1H, *J* = 3.5 Hz), 7.55–7.45 (m, 6H), 7.39–7.34 (m, 3H), 7.15 (d, 1H, *J* = 7.0 Hz) [Ar-H]; ^13^C NMR (DMSO-*d*_*6*_, 125 MHz) δ150.3, 148.5, 140.7, 136.0, 129.6, 129.4, 129.3, 129.3, 129.2, 128.8, 124.8, 123.1, 120.8, 120.8, 118.5, 114.4 [Ar-C]; LC-MS: 372.13 [M + 1]^+^.

#### 2.2.19. 3-(5-Phenylimidazo[2,1*-b*]thiazol-6-yl)phenyl methylsulfamate (1i)

Purified by flash column chromatography (silica gel, hexane:ethyl acetate 70%:30% v/v then switching to hexane:ethyl acetate 65%:35% v/v); pale yellow solid; yield: 43%; mp: 161–164°C; ^1^H NMR (DMSO-*d*_*6*_, 500 MHz) δ 8.18 (brs, 1H, NH), 7.70 (d, 1H, *J* = 4.5 Hz), 7.55–7.43 (m, 7H), 7.37 (t, 1H, *J* = 8.0 Hz), 7.33 (d, 1H, *J* = 4.5 Hz), 7.14 (dd, 1H, *J* = 1.5 Hz, 8.0 Hz) [Ar-H], 2.61 (d, 3H, *J* = 4.5 Hz, NH-CH_3_); ^13^C NMR (DMSO-*d*_*6*_, 125 MHz) δ 149.9, 148.5, 140.6, 136.3, 129.8, 129.5, 129.4, 129.3, 128.8, 124.9, 123.2 120.5, 120.2, 118.4, 114.4 [Ar-C], 29.1 (NH-CH_3_); LC-MS: 386.12 [M + 1]^+^.

#### 2.2.20. 3-(5-Phenylimidazo[2,1*-b*]thiazol-6-yl)phenyl piperidine-1-sulfonate (1j)

Purified by flash column chromatography (silica gel, hexane:ethyl acetate 75%:25% v/v then switching to hexane:ethyl acetate 72%:28% v/v); yellow solid; yield: 20%; mp: 112–115°C; ^1^H NMR (CDCl_3_, 500 MHz) δ 7.55–7.42 (m, 7H), 7.40 (s, 1H), 7.31–7.29 (m, 1H), 7.20 (d, 1H, *J* = 7.5 Hz), 6.94 (s, 1H) [Ar-H], 3.29 (s, 4H, piperidine CH_2_-N-CH_2_), 1.63 (s, 4H, piperidine CH_2_-CH_2_-N-CH_2_-CH_2_), 1.55 (d, 2H, *J* = 4.5 Hz, piperidine N-CH_2_-CH_2_-CH_2_); ^13^C NMR (CDCl_3_, 125 MHz) δ 150.6, 148.8, 130.0, 129.8, 129.7, 129.6, 129.3, 129.1, 125.7, 124.2, 123.6, 123.6, 121.1, 120.8, 117.9, 116.0, 115.5, 114.2 [Ar-C], 48.0 (piperidine CH_2_-N-CH_2_), 25.1 (piperidine CH_2_-CH_2_-N-CH_2_-CH_2_), 23.5 (piperidine N-CH_2_-CH_2_-CH_2_); LC-MS: 440.11 [M + 1]^+^.

#### 2.2.21. 3-(5-(3-(Methylsulfonyl)phenyl)imidazo[2,1*-b*]thiazol-6-yl)phenyl sulfamate (2h)

Purified by flash column chromatography (silica gel, hexane:ethyl acetate 75%:25% v/v then switching to hexane:ethyl acetate 72%:28% v/v); white solid; yield: 13%; mp: 150–153°C; ^1^H NMR (Methanol-*d*_*4*_, 500 MHz) δ 8.11–8.07 (m, 2H), 7.92–7.89 (m, 2H), 7.82 (t, 1H, *J* = 8.0 Hz), 7.49–7.44 (m, 4H), 7.34 (d, 1H, *J* = 3.5 Hz) [Ar-H], 3.17 (s, 3H, SO_2_-CH_3_); ^13^C NMR (Methanol-*d*_*4*_, 125 MHz) δ 152.4, 150.9, 143.5, 140.9, 135.7, 133.9, 132.1, 131.4, 130.8, 129.3, 129.2, 127.2, 123.7, 123.6, 122.9, 120.2, 117.4 [Ar-C], 44.2 (SO_2_-CH_3_); LC-MS: 450.01 [M + 1]^+^.

#### 2.2.22. 3-(5-(3-(Methylsulfonyl)phenyl)imidazo[2,1*-b*]thiazol-6-yl)phenyl methylsulfamate (2i)

Purified by flash column chromatography (silica gel, hexane:ethyl acetate 70%:30% v/v then switching to hexane:ethyl acetate 65%:45% v/v); yellow solid; yield: 92%; mp: >250°C; ^1^H NMR (DMSO-*d*_*6*_, 500 MHz) δ 8.20 (brs, 1H, NH), 8.02–8.00 (m, 2H), 7.87–7.85 (m, 1H), 7.82–7.78 (m, 2H), 7.43–7.37 (m, 4H), 7.19–7.16 (m, 1H) [Ar-H], 3.25 (s, 3H, SO_2_-CH_3_), 2.62 (s, 3H, NH-CH_3_); ^13^C NMR (DMSO-*d*_*6*_, 125 MHz) δ 150.1, 149.4, 141.9, 141.7, 135.9, 134.3, 130.8, 130.7, 130.1, 127.4, 127.0, 125.1, 121.5, 120.9, 120.4, 118.6, 114.7 [Ar-C], 43.4 (SO_2_-CH_3_), 29.2 (NH-CH_3_); LC-MS: 464.12 [M + 1]^+^.

#### 2.2.23. 3-(5-(3-(Methylsulfonyl)phenyl)imidazo[2,1*-b*]thiazol-6-yl)phenyl piperidine-1-sulfonate (2j)

Purified by flash column chromatography (silica gel, hexane:ethyl acetate 75%:25% v/v then switching to hexane:ethyl acetate 70%:30% v/v); off-white crystal, yield: 22%; mp: 76–79°C; ^1^H NMR (CDCl_3_, 500 MHz) δ 8.02 (d, 2H, *J* = 7.5 Hz), 7.71 (d, 1H, *J* = 5.0 Hz), 7.59 (s, 1H), 7.42–7.38 (m, 3H), 7.16 (d, 1H, *J* = 7.5 Hz), 6.99 (s, 1H) [Ar-H], 3.30 (s, 4H, piperidine CH_2_-N-CH_2_), 3.10 (s, 3H, SO_2_-CH_3_), 1.64 (s, 4H, piperidine CH_2_-CH_2_-N-CH_2_-CH_2_), 1.56 (d, 2H, *J* = 4.0 Hz, piperidine N-CH_2_-CH_2_-CH_2_); ^13^C NMR (CDCl_3_, 125 MHz) δ 150.6, 149.6, 142.4, 134.7, 131.1, 130.4, 128.1, 128.0, 126.0, 121.5, 120.9, 117.7 [Ar-C], 48.1 (piperidine CH_2_-N-CH_2_), 44.7 (SO_2_-CH_3_), 25.1 (piperidine CH_2_-CH_2_-N-CH_2_-CH_2_), 23.5 (piperidine N-CH_2_-CH_2_-CH_2_); LC-MS: 518.06 [M + 1]^+^.

### 2.3. Urease inhibition assay

Compounds **1a-1j** and **2a-2j** were tested to evaluate the inhibitory potential activity employing the indophenol method [44] with a slight modification [45]. Reaction mixture comprising 40 μL of buffer (K_2_HPO_4_: 10 mmol/L, urea: 100 mmol/L, LiCl_2_: 10 mmol/L, EDTA: 1 /L mmol, pH = 8.2) and 5 U/mL enzyme (10 μL) were pre-incubated with 10 μL of synthesized compounds (1 mM) and substrate urea (1 mM) were incubated at room temperature in 96 well plate for half an hour. After the incubation at room temperature, absorbance was taken at specific wavelength (630 nm) employing a microplate reader (Bio-Tek, USA) with R1 and R2 reagents in each well. The reactions were measured in triplicate fashion. To calculate the inhibition percentage following equation was used.

Percentage inhibition = 100 –[tested compound/control compound] × 100.

Thiourea was used as positive control and to estimate the IC_50_ values of different concentrations of the synthesized compounds **1a-1j** and **2a-2j** using GraphPad Prism 8.0.2 was used to observe the data (GraphPad, California, USA).

### 2.4. Enzyme kinetics studies

The type of enzyme inhibition was determined by Michaelis-Menten kinetics experiments. In order to investigate the potential mechanism of action of the potent compound **2c**, detailed kinetics studies were conducted. The initial inhibitory efficacy rates of enzyme activity were measured at four different concentrations of substrate (0.5, 1.0, 1.5 and 2.0 mM) in the presence or absence of compound **2c** (0, 1.5, 3.0, 4.5 μM) against urease.

### 2.5. Anti-helicobacter activity testing

#### 2.5.1. Bacterial culture

The *H*. *pylori* reference strain ATCC 43504 (DSM 21031) was purchased from the German collection of microorganisms and cell cultures, GmbH (DSMZ; Germany). *H*. *pylori* was grown on Brucella agar plates (Sigma, USA) supplemented with 10% horse serum (Sigma, USA) for 72 h at 37 ˚C under 98% humidity and microaerophilic conditions (5% O_2_, 10% CO_2_, and 85% N_2_) using microaerophilic CampyGen 2.5 L pack (Oxoid, thermo scientific, UK) and anaerobic jar (Biomerieux, France). *H*. *pylori* culture was inspected after 3 to 5 days. The *E*. *coli* reference strain ATCC 25922 was purchased from the American Type Culture Collection (ATCC, Manassas, VA, USA). *E*. *coli* was grown on nutrient agar plates (Sigma, USA) for 24 h at 37 ˚C under aerobic conditions. Bacterial load was adjusted using a DensiCHEK PLUS device (Biomerieux, France). A regular gram stain (Biomerieux, France) was performed to confirm the identity and the morphology of *H*. *pylori* and *E*. *coli* as well as to confirm the absence of any contamination. Furthermore, a regular spotting checkup was also performed behind the micro broth dilution testing followed by a gram stain to confirm the identity of the bacteria and the absence of any potential contamination.

#### 2.5.2. Antimicrobial susceptibility testing

For MIC, the agar dilution method was performed as per the CLSI guidelines. 5 μL of the *H*. *pylori* suspension (2.0 McFarland) was plated on the Mueller–Hinton agar (Sigma, USA) supplemented with 10% horse serum including various concentrations of compounds **1a-1j** and **2a-2j** (0.8 mM, 0.4 mM, 0.2 mM, 0.1 mM, 0.05 mM, 0.025 mM, 0.0125 mM, and 0.006 mM). The MIC was determined based on the lowest concentration that resulted in visible growth inhibition on the agar well plate. For the micro broth dilution test, compounds **1a-1j** and **2a-2j** were prepared by 2-fold serial dilution from a concentration of 0.8 mM to 0.006 mM and added to an equal *H*. *pylori* suspension of 1.0 McFarland in Mueller–Hinton broth (Sigma, USA) for 72 h. Final optical density of the bacterial suspension was measured by spectrophotometry using Synergy H1 microplate reader (BioTek, USA) at 600 nm. The half maximal inhibitory concentration (IC_50_) was calculated using Graph Pad Prism 8.02 for windows (Graph Pad Inc., La Jolla, CA, USA). All the tested derivatives compounds were dissolved in dimethyl sulfoxide (DMSO, Sigma, USA). In order to exclude the antimicrobial activity of the DMSO, the maximum concentration of the used DMSO did not exceed the 2% in the highest tested concentration (0.8 mM) of the 20 compounds. The 2% DMSO did not show any inhibitory effect on *H*. *pylori* growth. Amoxicillin (Sigma, USA) (10 μg/mL and 0.007 μg/mL) and (12 μg/mL and 3 μg/mL) was used as a control antibiotic to confirm the agar dilution protocol for *H*. *pylori* and *E*. *coli*, respectively. Acetohydroxamic acid (AHA, Sigma, CAS number: 546-88-3) was used as a positive anti-urease control. All antimicrobial susceptibility experiments were repeated twice.

### 2.6. Cytotoxicity testing against AGS gastric cells

#### 2.6.1. Mammalian cell culture

Human gastric adenocarcinoma AGS cells (ATCC CRL-1739) were purchased from the German Cell Lines Service (CLS, Germany) and cultured in RPMI-1640 medium (Sigma, USA) supplemented with 10% fetal bovine serum (FBS, Sigma, USA) and streptomycin-penicillin (100 μg/mL and 100 IU/mL) (Sigma, USA). Cells were incubated at 37 ˚C in a humidified atmosphere with 5% CO_2_ until 80–90% confluency.

#### 2.6.2. Cytotoxicity assay

The (4, 5-dimethythiazol-2-yl) -2, 5-diphenyltetrazolium bromide (MTT, Sigma, USA) assay was used to assess the cytotoxicity of the five most significant compounds. AGS cells (5×10^3^/well) were seeded in 96-well plates for 24 h and treated with various concentrations (0.8 mM, 0.4 mM, 0.2 mM, 0.1 mM, 0.05 mM, 0.025 mM, 0.0125 mM, and 0.006 mM) of the five most significant anti-urease imidazothiazole derivatives compounds (compound **1a**, **1d**, **1h**, **2d**, and **2f**). After 48 h treatment, 100 μL of MTT solution (50 μg/mL) was added to each well, and the cells were incubated for additional 2 h at 37 ˚C. The solubilization of MTT crystals was accomplished by adding 100 μL of DMSO followed by 10 min of incubation. The plates were shaken for 5 min to dissolve the MTT formazan crystals. The optical density of each well was determined using a Synergy H1 microplate reader (Biotek, USA) at a wavelength of 570 nm. The IC_50_ of the tested compounds was calculated using GraphPad Prism 8.0.2 for windows (GraphPad Inc., La Jolla, CA, USA) from two repeated experiments.

### 2.7. Caco-2 permeability testing

#### 2.7.1. Permeability

The apparent permeability coefficient (P_app_) of the test compound was calculated as follows:

$$P_{app}(\mathrm{cm}/s)=\frac{V_{R}*C_{R,end}}{\Delta t}*\frac{1}{A*(C_{D,mid}‐C_{R,mid})}$$

where V_R_ is the volume of the receiver chamber. C_R,end_ is the concentration of the test compound in the receiver chamber at the end time point, Δt is the incubation time and A is the surface area of the cell monolayer. C_D,mid_ is the calculated mid-point concentration of the test compound in the donor side, which is the mean value of the donor concentration at time 0 minute and the donor concentration at the end time point. C_R,mid_ is the mid-point concentration of the test compound in the receiver side, which is one half of the receiver concentration at the end time point. Concentrations of the test compound were expressed as peak areas of the test compound.

#### 2.7.2. Recovery of the test compound from the permeability assay

The recovery of the test compound was calculated as follows:

$$Recovery\;(\%)=\frac{V_{D}*C_{D,end}+V_{R}*C_{R,end}}{V_{D}*C_{D0}}*100$$

where V_D_ and V_R_ are the volumes of the donor and receiver chambers, respectively. C_D,end_ is the concentration of the test compound in the donor sample at the end time point. C_R,end_ is the concentration of the test compound in the receiver sample at the end time point. C_D0_ is the concentration of the test compound in the donor sample at time zero. Concentrations of the test compound are expressed as peak areas of the test compound.

#### 2.7.3. Fluorescein assessment for permeability assays

Fluorescein was used as the cell monolayer integrity marker. Fluorescein permeability assessment (in the A-B direction at pH 7.4 on both sides) was executed after the permeability assay for the test compound. The cell monolayer that had a fluorescein permeability below 1.5 x 10^−6^ cm/s for Caco-2 cells was considered intact, and the permeability result of the test compound from intact cell monolayer is reported.

### 2.8. *In silico* studies protocols

#### 2.8.1. Structure selection and preparation

To investigate the significant interactions of the inhibitors with the enzyme urease, the employment of docking protocols Jack bean urease crystallographic structure (3LA4) and *H*. *pylori* urease was downloaded from RCSB PDB library and prepared for docking analysis [46, 47]. Prior to docking studies, construction of the compounds and the enzyme (urease) were formed as following; Protonate3D [48] technique was used to protonate the enzyme structure employing MOE (molecular modelling program) [49]. To avoid the energy deficit as possible, Amber99 force field were applied to the crystallographic molecules. To overcome the loss of active pocket during the calculation of energy minimization, small force was applied to the backbone atom in their specific location. Consequently, water molecules and ligands were removed from the protein and polar hydrogen atoms were added to the X-ray structure in specific geometries with the MOE.

#### 2.8.2. Compounds preparation

Using “wash” module, 3D structural coordinates of powerful inhibitors (**1a**, **1d**, **1h**, **2b**, **2c**, and **2f**) were generated with MOE and assigned protonation and ionization states in the physiological pH range. Subsequently, MMFF94x force field were used to minimize the energy of compound structure for docking analysis.

#### 2.8.3. Docking studies

Different software such as BioSolveIT, GmbH Germany [50] and LeadIT were used to investigate the binding interactions. Receptor was loaded through load or prepare receptor utility of LeadIT software as well as metal was selected as part of it. General spacing of amino acid residues (9.0) was used to determine the receptor binding site. FlexX program utility of LeadIT was used to dock the target compounds or inhibitors, in which 50 different conformations for each complex (protein-ligand complex) were selected depending on binding affinity free energy for selective molecules for docking studies. Docking parameters remained as default and top 30 highest scoring poses were selected for further study [51]. To find out the best receptor binding pose, stable and lowest free binding energy pose was identified and visualized with Discovery Studio Visualizer v4 [52], to explore the complex for binding interactions.

#### 2.8.4. Molecular dynamics simulations

Protein Data Bank was used to retrieve the crystallographic three-dimensional structure of Jack bean urease PDB ID: 3LA4 (www.pdb.org) [46]. GROMOS96 force field was used for the protonation and manipulation of the protein with 43a1 parameter adjustment. Groningen Machine for Chemical Simulation (GROMACS), module 5.1.4 was employed for the molecular dynamic simulations according to earlier described protocol with slight modifications [41, 53–55]. PRODRG online server was used for the parameterization of the potent compounds, **1h** and **2c** [56]. Molecular description and visualization were done with MOE and VMD [57]. For the neutralization of receptor, addition of water molecules and assimilation of counter ions was done in the crystallographic structure. Consequently, equilibration and minimization of energy was acquired with NVT (amount of substance (N), volume (V) and temperature (T)) and NPT (amount of substance (N), constant-pressure (P) and constant-temperature (T)) (100 ps) sequential runs with the confinement of heavy atoms of protein.

Results were submitted to 30 ns molecular dynamics simulations with 2 fs for each simulation after the minimization of energy. The minimization of energy was achieved using GROMOS96 forcefield having 43a1 parameter set. All the simulations were done by PBC (periodic boundary conditions). Minimization of energy was achieved by the steepest descent method. NVT and NPT runs used Berendsen thermostat for temperature (almost 303 K) and Parrinello-Rehman barostat for pressure coupling (almost 1.01 bar). PME (Particle Mesh Ewald) and terminated radius of 10 Å protocol were noticed. Subsequently, XMGRACE, version 5.1.19 was used to plot the RMSD (root mean square deviations), variations and radius of rotation [58].

## 3. Results and discussion

### 3.1. Chemistry

**Scheme 1:**
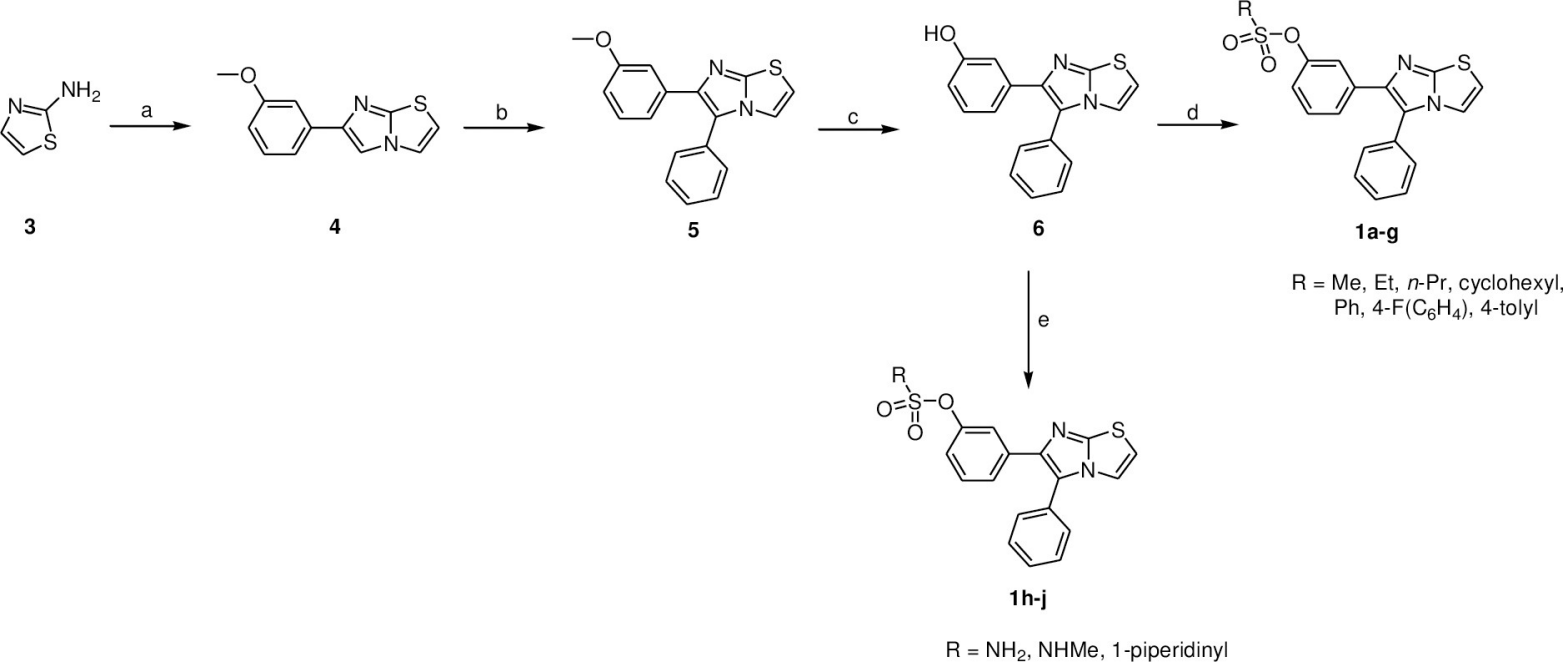
Reagents and reaction conditions: a) α-bromo-3-methoxyacetophenone, absolute EtOH, reflux, 16 h, 80%; b) iodobenzene, Pd(OAc)_2_, Cs_2_CO_3_, PPh_3_, anhydrous DMF, 80°C, 12 h, 30%; c) BBr_3_, anhydrous CH_2_Cl_2_, -78°C, 1 h; rt, overnight, 40%; d) triethylamine, appropriate sulfonyl chloride derivative, anhydrous DMF, 0°C then rt, 2 h; e) appropriate sulfamoyl chloride derivative, anhydrous DMAc, 0°C then rt, overnight (compounds **1h,i**); piperidine-1-sulfonyl chloride, NaH, anhydrous DMAc, 0°C then rt, overnight (compounds **1j**).

The synthesis of the intended derivatives **1a-1j** is described in Scheme 1. Heterocyclization reaction between the 2-aminothiazole (**3**) and α-bromo-3-methoxyacetophenone in refluxing ethanol delivered compound **4** [52]. Under Heck reaction conditions, the latter gave compound **5** when reacted with iodobenzene. Demethylation of **5** using boron tribromide yielded **6** [43, 59]. The target sulfonate derivatives (**1a-1g**) were formed by reacting intermediate (**6**) with the appropriate sulfonyl chloride reagent in the presence of triethylamine. Whereas the synthesis of target sulfamate derivatives **1h-1j** were furnished using the appropriate sulfamoyl chloride reagent.

**Scheme 2:**
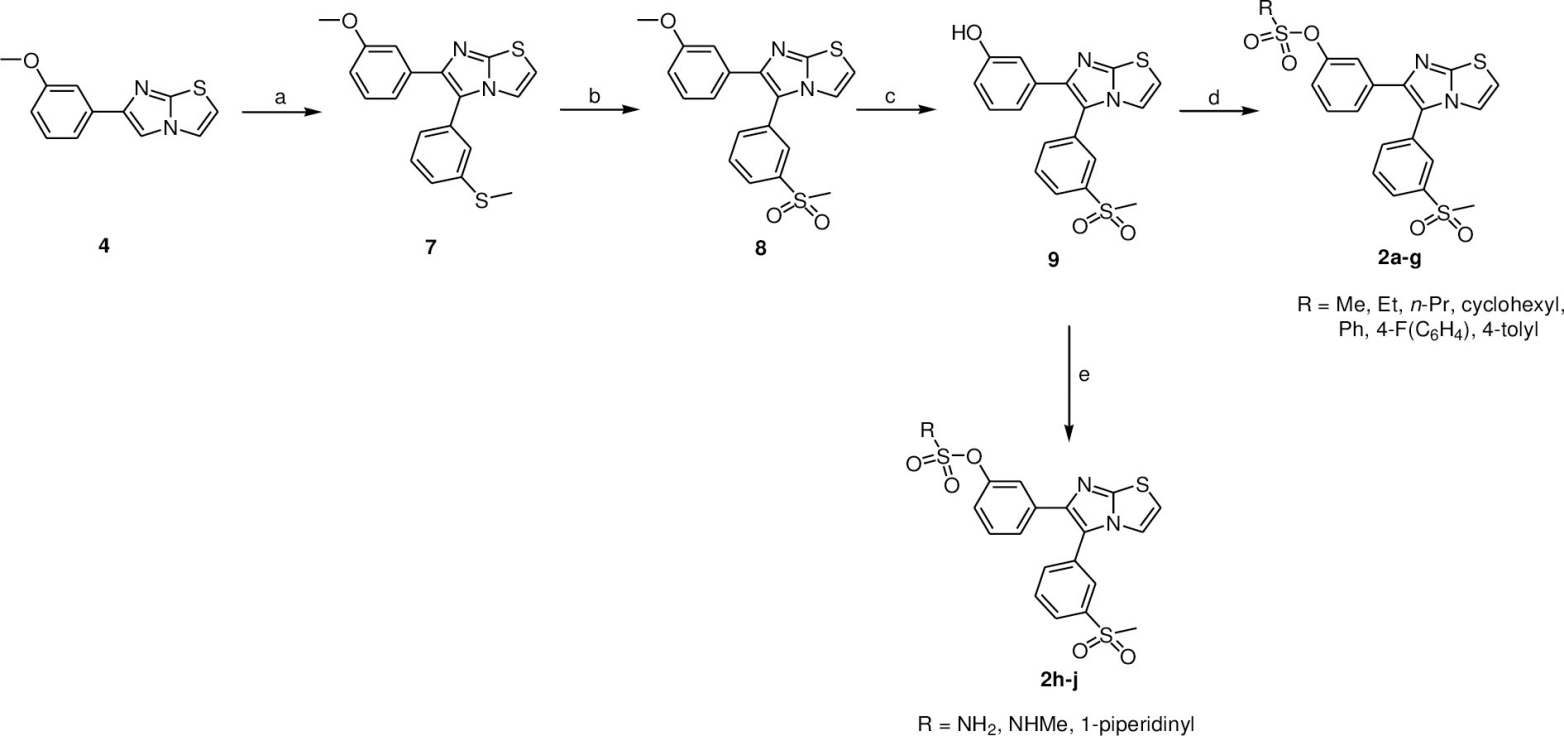
Reagents and reaction conditions: a) 3-Iodothioanisole, Pd(OAc)_2_, Cs_2_CO_3_, PPh_3_, anhydrous DMF, 80°C, 12 h, 40%; b) Oxone, MeOH, H_2_O, rt, 16 h, 78%; c) BBr_3_, anhydrous CH_2_Cl_2_, -78°C, 1 h; rt, overnight, 40%; d) triethylamine, appropriate sulfonyl chloride derivative, anhydrous DMF, 0°C then rt, 2 h; e) appropriate sulfamoyl chloride derivative, anhydrous DMAc, 0°C then rt, overnight (compounds **2h,i**); piperidine-1-sulfonyl chloride, NaH, anhydrous DMAc, 0°C then rt, overnight (compounds **2j**).

On the other side, compounds **2a-2j** were synthesized as shown in Scheme 2. Compound **4** was reacted with 3-iodothioanisole to yield intermediate **7** using Heck reaction conditions. Moreover, the sulfide group of compound **7** was oxidized to sulfone to deliver compound **8** using oxone [59], followed by demethylation using boron tribromide to obtain 3-(5-3-(methylsulfonyl)phenyl)imidazo[2,1-*b*]thiazol-6-yl)phenol (**9**) [43]. The construction of the sulfonates **2a-2g** and sulfamates **2h-2j** (Scheme 2) was carried out under the same conditions as described Scheme 1.

### 3.2. Urease inhibition assays and structure-activity relationship analyses

We started our discovery process by subjecting the developed sulfonates **(1a-1g, 2a-2g)** and sulfamates **(1h-1j, 2h-2j)** to urease enzymatic assay using indophenol method (Table 1).

**Table 1:** Urease inhibition of sulfonate and sulfamate derivatives (1a-1j and 2a-2j).

Compound No.	Potency against urease IC_50_ ± SEM (μM)^a^
**1a**	4.10 ± 0.03
**1b**	4.01 ± 0.11
**1c**	4.04 ± 0.09
**1d**	3.09 ± 0.07
**1e**	4.30 ± 0.01
**1f**	5.17 ± 0.01
**1g**	3.22 ± 0.06
**1h**	2.96 ± 0.02
**1i**	5.51 ± 0.17
**1j**	6.97 ± 0.09
**2a**	5.01 ± 0.03
**2b**	4.97 ± 0.11
**2c**	2.94 ± 0.05
**2d**	4.46 ± 0.12
**2e**	5.21 ± 0.03
**2f**	7.19 ± 0.09
**2g**	5.59 ± 0.04
**2h**	4.72 ± 0.13
**2i**	4.74 ± 0.21
**2j**	5.82 ± 0.02

^a^ The results are expressed as means of triplicate assays ± SEM.

Generally, all the synthetic compounds revealed potent activity (IC_50_ values in the range of 2.94 to 7.19 μM). Compound **2c** was the most potent compound in the series, having an IC_50_ value of 2.94 ± 0.05 μM, 8-folds more potent than the positive control (thiourea; IC_50_ = 22.3 ± 0.11 μM). Derivatives **1a-1j** showed comparable inhibitory activity compared to their sulfone counterparts **2a-2j**. Aliphatic sulfonate derivatives (**1a-d** & **2a-d**) were relatively more potent than aromatic sulfonates and sulfamates. Among the aliphatic derivatives **1a-d**, no significant change was observed upon increasing sulfonate chain length (IC_50_s of compounds **1a**, **1b**, and **1c** are 4.10 ± 0.03, 4.01 ± 0.11, 4.04 ± 0.09 μM, respectively), while replacing the chain with a cyclohexane moiety improved the inhibitory activity (IC_50_ of compounds **1d** is 3.09 ± 0.07 μM). A similar trend was observed in compounds **2a-d** which exhibited less potent activity in comparison to **1a-d** except in the case of compound **1c** where it showed the most potent activity against urease (IC_50_s of compounds **2a**, **2b**, **2c**, and **2d** are 5.01 ± 0.03, 4.97 ± 0.11, 2.94 ± 0.05, 4.46 ± 0.12 μM, respectively). The aromatic sulfonate derivatives **1e-g** & **2e-g** were less potent compared to their aliphatic derivatives except in the case of compound **1g** which was substituted with methyl group at position 4 of the terminal sulfonate chain. On the other side, the less potent compound possesses contains a fluorine substituent at the same position, indicating that electron withdrawing groups are not tolerated on this position (IC_50_s of compounds **1e**, **1f**, **1g**, **2e**, **2f**, and **2g** are 4.30 ± 0.01, 5.17 ± 0.01, 3.22 ± 0.06, 5.21 ± 0.03, 7.19 ± 0.09, 5.59 ± 0.04 μM, respectively). In addition, the sulfamate derivatives **1h-1j** & **2h-2j** showed moderate activities, except compound **1h** which possesses a sulfamate moiety with an inhibitory activity similar to that of compound **2c**. Including substituents on the sulfamate group reduced the inhibitory activity. This is evident when comparing the activities of the unsubstituted compounds sulfamates **1h-1j** & **2h-2j**, with the substituted ones (IC_50_s of compounds **1h**, **1i**, **1j**, **2h**, **2i**, and **2j** were 2.96 ± 0.02, 5.51 ± 0.17, 6.97 ± 0.09, 4.72 ± 0.13, 4.74 ± 0.21, 5.82 ± 0.02 μM, respectively).

### 3.3. Mechanism of inhibition

Kinetics studies were performed to determine the mode of action of compound **2c**. The mechanism of inhibition of urease was determined by enzyme kinetics using urea as a substrate. To determine the type of inhibition, analyze the effect of inhibitor on V_max_ and K_*m*_, a Lineweaver-Burk plot (reciprocal of reaction rates 1/S and reciprocal of substrate concentrations 1/V) was used.

**Fig 3:**
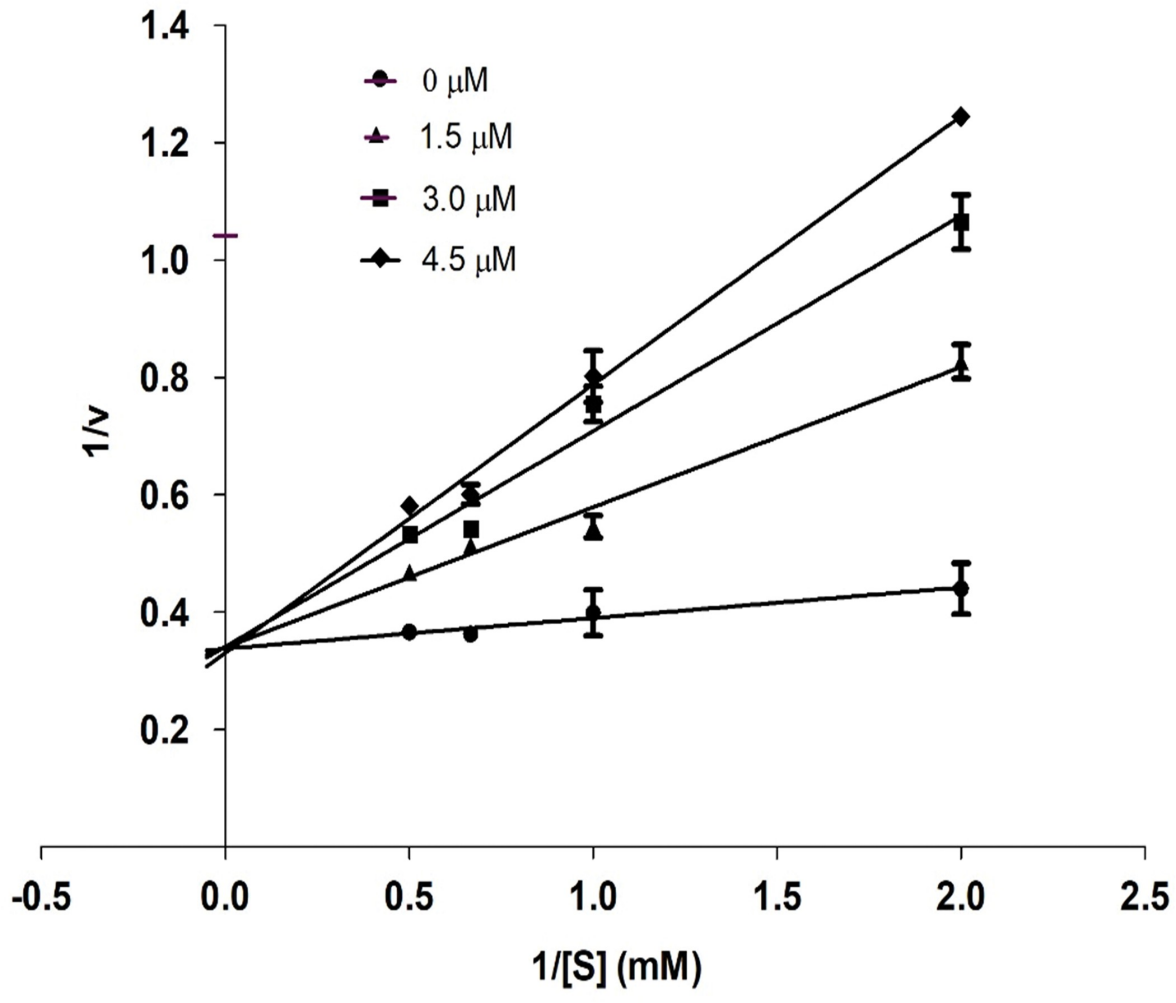
Inhibition of urease by compound 2c. Lineweaver-Burk graph exhibiting reciprocal rate of reaction 1/V against reciprocal of substrate 1/S.

Different concentrations of inhibitor **2c** and substrate were used to perform kinetics. Four concentrations (0, 1.5, 3.0, 4.5 μM) of inhibitor **2c** and four concentrations (0.5, 1.0, 1.5 and 2.0 mM) of substrate were used. Compound **2c** exhibited competitive inhibition with urea within the active pocket of urease. An increase in K_*m*_ does not change the value of V_*max*_ along lines intersecting at the y-axis in a competitive inhibition as shown in Fig 3.

### 3.4. Screening against *Helicobacter pylori*

The developed compounds were screened for their activity against *H*. *pylori*. The known urease inhibitor, acetohydroxamic acid, was utilized as a positive control in this assay. The minimum inhibitory concentration (MIC) and IC_50_ values are depicted in Table 2.

**Table 2:** Antibacterial activity of the target compounds 1a-1j and 2a-2j and acetohydroxamic acid against *H*. *pylori*^a^.

Compound No.	MIC value against *H*. *pylori* ± SEM (mM)	IC_50_ value against *H*. *pylori* ± SEM (mM)
**1a**	0.019 ± 0.004	0.023 ± 0.009
**1b**	0.80 ± 0.000	0.082 ± 0.029
**1c**	0.8 ± 0.000	0.364 ± 0.000
**1d**	0.031 ± 0.011	0.02 ± 0.003
**1e**	0.4 ± 0.000	0.153 ± 0.033
**1f**	0.2 ± 0.000	0.147 ± 0.000
**1g**	0.15 ± 0.029	0.037 ± 0.006
**1h**	0.05 ± 0.000	0.028 ± 0.001
**1i**	0.8 ± 0.000	0.285 ± 0.092
**1j**	0.2 ± 0.000	0.131 ± 0.002
**2a**	0.25 ± 0.050	0.118± 0.005
**2b**	0.8 ± 0.000	0.255 ± 0.035
**2c**	0.8 ± 0.000	0.281 ± 0.021
**2d**	0.022 ± 0.004	0.015 ± 0.003
**2e**	0.409 ± 0.391	0.331 ± 0.021
**2f**	0.038 ± 0.013	0.023 ± 0.008
**2g**	0.275 ± 0.125	0.103 ± 0.088
**2h**	0.4 ± 0.000	0.225 ± 0.005
**2i**	0.25 ± 0.050	0.123 ± 0.015
**2j**	0.8 ± 0.000	0.288 ± 0.018
**Acetohydroxamic acid (AHA)**	12.5 ± 0.000	7.38 ± 0.320

^a^ The results are expressed as means of triplicate assays ± SEM.

The results indicated that the methanesulfonate derivatives **1a** and **2a** are more potent than the corresponding ethanesulfonate (**1b** and **2b**) and *n*-propanesulfonate (**1c** and **2c**) analogues. In addition, the cyclohexanesulfonate derivatives **1d** and **2d** showed higher activity than the corresponding aryl sulfonate analogues **1e-g** and **2e-g**. Furthermore, *N-*substitution of the sulfamate derivatives indicated to be detrimental for the antibacterial activity. The free sulfamate derivative **1h** is more potent than the *N*-methylsulfamate and 1-piperidinesulfonate analogues **1i** and **1j**, respectively.

As indicated from Table 2, all the developed compounds **1a-1j** and **2a-2j** are more potent than the reference molecule, acetohydroxamic acid. The low antibacterial activity of the acetohydroxamic acid might be due to its low lipophilicity which might hinder its penetration into the bacterial cells. Among all the target compounds, **1a**, **1d**, **1h**, **2d**, and **2f** exhibited the strongest antibacterial activity against *H*. *pylori*. As such, these five compounds were then subjected to further investigations.

### 3.5. Cytotoxicity evaluation of compounds 1a, 1d, 1h, 2d, 2f, and acetohydroxamic acid against AGS gastric cells

Following the promising anti-helicobacter activity of compounds **1a**, **1d**, **1h**, **2d**, and **2f,** their cytotoxicity against AGS gastric cancer cells was studied. The purpose of this study was to investigate the selectivity of the five compounds against *H*. *pylori* compared to AGS cells. As a result, the IC_50_ values of compounds **1a**, **1d**, **1h**, **2d**, and **2f,** in addition to acetohydroxamic acid are shown in Table 3. In comparison with their IC_50_ values against *H*. *pylori* (Table 2), the selectivity indexes of compounds **1a**, **1d**, **1h**, **2d**, and **2f** toward *H*. *pylori vis-à-vis* AGS cells are 3.09, 6.05, 5.18, 2.0, and 1.95, respectively. Therefore, by adjusting the concentration, the compounds can exert antibacterial activity against *H*. *pylori* with minimal or no cytotoxicity on gastric cells. It is noteworthy that the selectivity indexes of compounds **1a**, **1d**, and **1h** that lack *meta*-sulfone group on the phenyl ring at position 5 of the imidazothiazole nucleus are higher than those of compounds **2d** and **2f** possessing sulfone groups.

**Table 3:** Cytotoxicity evaluation by MTT assay (IC_50_, mM) of compounds 1a, 1d, 1h, 2d, 2f, and acetohydroxamic acid^a^.

Compound No.	IC_50_ value against AGS cells ± SEM (mM)	Selectivity index (SI)
**1a**	0.071 ± 0.017	3.09
**1d**	0.121 ± 0.044	6.05
**1h**	0.145 ± 0.045	5.18
**2d**	0.030 ± 0.011	2.0
**2f**	0.045 ± 0.005	1.95
**Acetohydroxamic acid (AHA)**	5.9 ± 0.3	-

^a^ The IC_50_ were calculated from two repeated independent experiments and presented as ± SEM.

Furthermore, increasing the concentration of the tested compounds led to cytotoxic effect against those cells. These compounds might have another application as anti-gastric cancer candidates. They can be useful in cases of simultaneous *H*. *pylori* infection and gastric cancer. Further studies are needed for deeper investigation of this cytotoxicity and its underlying molecular mechanism(s).

### 3.6. Selectivity of the most promising compounds against *H*. *pylori* and *E*. *coli*

*H*. *pylori* is a urease-positive bacteria while *Escherichia coli* (*E*. *coli)* is a urease-negative bacteria. Herein, we planned to validate the mechanism of action of the the most promising anti-helicobacter compounds **1a**, **1d**, **1h**, **2d**, and **2f** by investigating their activity against *E*. *coli*, to test whether their antibacterial potency differ in urease-positive and urease-negative bacteria. The results are summarized in Table 4. The five compounds showed a very promising inhibitory effect against *H*. *pylori* with no activity against *E*. *coli*. It could be concluded that there is a correlation between the antibacterial activity of our urease inhibitors and the presence of urease enzyme in *H*. *pylori*. This corroboration might confirm the mechanism of action of the developed compounds as urease inhibitors.

**Table 4:** Selectivity of compounds 1a, 1d, 1h, 2d, 2f, and acetohydroxamic acid against *H*. *pylori* (urease positive) and *E*. *coli* (urease negative)^a^.

Compound No.	*H*. *pylori* (Urease^+^)		*E*. *coli* (Urease^-^)	
	MIC (mM) ±SEM	IC_50_ (mM) ±SEM	MIC (mM) ±SEM	IC_50_ (mM) ±SEM
**1a**	0.019 ±0.004	0.023 ±0.009	> 0.8	No effect
**1d**	0.031 ±0.011	0.02 ±0.003	> 0.8	No effect
**1h**	0.05 ±0.000	0.028 ±0.001	> 0.8	No effect
**2d**	0.022 ±0.004	0.015±0.003	> 0.8	No effect
**2f**	0.038 ±0.013	0.023±0.008	> 0.8	No effect
**Acetohydroxamic acid (AHA)**	12.5±0.000	7.38 ±0.320	100	39.91± 1.78

^a^ The results were calculated as means of three independent experiments ± SEM.

### 3.7. Caco-2 permeability

Caco-2 permeability assay is commonly utilized to measure the potential oral absorption of the tested compounds [60]. The most active anti-helicobacter compounds **1a**, **1d**, **1h**, **2d**, **2f,** and the reference drugs labetalol and propranolol were tested for their permeability in Caco-2 A-B at pH 6.5–7.4 and at their MIC value against *H*. *pylori*. The results are shown in Table 5. The two cyclohexanesulfonate derivatives **1d** and **2d**, as well as the 4-fluorobenzenesulfonate derivative **2f** showed the lowest permeability and practically their oral absorption is estimated to be very low. This is an interesting finding as their oral administration can lead to only targeting the GIT without being orally absorbed and hence devoid of systemic side effects. As can be noticed from their structures, compounds **1d**, **2d**, and **2f** are less polar than **1a** (methanesulfonate) and **1h** (free sulfamate). This might lead to lower aqueous solubility and hence low oral absorption.

**Table 5:** Caco-2 A-B permeability of compounds 1a, 1d, 1h, 2d, 2f, and the reference drugs labetalol and propranolol at pH 6.5–7.4^a^.

Tested compound	Test concentration (μM)	Permeability (10^−6^ cm/s)	% Recovery
**1a**	19	18.63 ± 0.79	34.38% ± 0.98%
**1d**	31	0.04 ± 0.01	62.98% ± 1.30%
**1h**	50	16.92 ± 0.87	32.56% ± 0.48%
**2d**	22	0.07 ± 0.02	73.45% ± 1.62%
**2f**	38	1.98 ± 0.23	71.82% ± 0.74%
**Labetalol**	10	19.16 ± 0.56	99.12% ± 0.42%
**Propranolol**	10	39.02 ± 0.44	88.70% ± 0.86%

^a^ The results are expressed as means of triplicate experiments ± SEM.

### 3.8. Molecular docking studies

**Fig 4:**
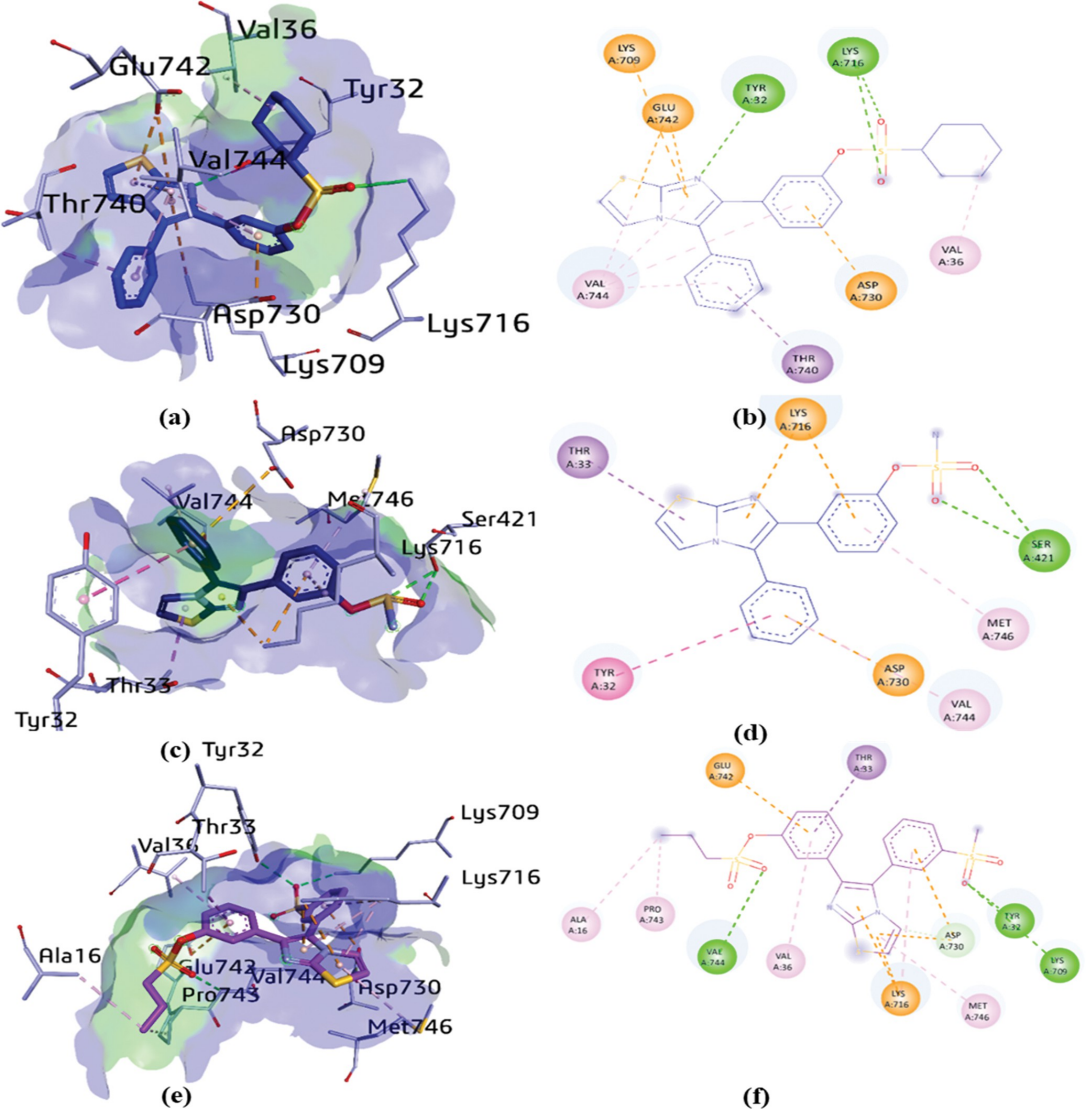
3D and 2D binding interactions of compounds **1d** (a, b), **1h** (c, d), and **2c** (e, f) with several amino acid residues. H-bonds presented in green, alkyl and π-alkyl in pink, π-sigma in purple, π-cation and anion in orange and π-π T shaped interactions in dark pink dotted lines.

The most potent compounds (**1d, 1h,** and **2c**) were docked inside the active pocket of urease to identify the important binding interactions with the amino acid residues of receptor (3LA4) [61]. The docking studies of compound **1d** within the active site of urease exhibited various vital binding interactions with the amino acid residues; THR740, ASP730, VAL36, LYS716, TYR32, LYS709, GLU742 and VAL744. The imidazole-thiazole ring revealed multiple interactions with LYS709, GLU742, TYR32 and VAL744 including π-cation (4.87 Å), π-anion (4.09 Å and 4.99 Å) hydrogen bonding (2.66 Å) and π-alkyl linkages (5.11 Å and 4.31 Å), respectively. The phenyl ring of compound **1d** exhibited a π-sigma and π-alkyl interactions between amino acid residues THR740 and VAL744 with distances of 3.89 Å and 4.75 Å, respectively. Conventional hydrogen bonding (3.22 Å and 2.94 Å) was displayed between O24 and O25 of sulfonate and LYS716. However, VAL744 and ASP730 exhibited a π-alkyl linkage (5.39 Å) and a π-anion interaction with phenyl ring of **1d**, respectively. Moreover, hydrophobic interaction (4.30 Å) was also observed between VAL36 and cyclohexane (Fig 4A and 4B).

In addition, the docking studies of compound **1h** within the active site of enzyme urease explored various important interactions with SER421, LYS716, THR33, TYR32, ASP730, VAL744 and MET746 amino acid residues. It exhibited hydrogen bond between the oxygen atoms (024 and O25) and SER421 (2.97 Å and 2.94 Å). Consequently, LYS716 residue showed π-cation interactions with the imidazole and phenyl ring of **1h** *via* distances of 4.06 Å and 4.20 Å, respectively. However, the thiazole ring exhibited a π-sigma linkage with amino acid residue THR33 with a distance of 3.65 Å. Additionally, the phenyl ring depicted multiple interactions with several amino acid residues like π-π T-shaped (TYR32; 5.26 Å), π-anion (ASP730; 4.71 Å) and π-alkyl (VAL744; 5.17 Å). Moreover, MET746 revealed a π-alkyl interaction (5.27 Å) with the phenyl ring of compound **1h** as shown in (Fig 4C and 4D).

The docking analysis of the most potent compound, **2c**, within the active pocket of urease indicated multiple interactions with many amino acid residues such as LYS709, TYR32, ASP730, MET746, LYS716, VAL36, VAL744, PRO743, ALA16, GLU742, and THR33. For the sake of evaluation of docking results of compound **2c**, it was depicted that TYR32 and LYS709 are involved in the formation of conventional hydrogen bonding (3.19 Å and 3.29 Å) with O30 of the mesyl group. In addition, O25 of the other sulfonate group presented a hydrogen bond with VAL744 at a distance of 3.28 Å. Consequently, LYS716 exhibited a π-cation interaction with the imidazole and the thiazole rings of compound **2c** with distances of 3.81 Å and 4.37 Å, respectively. Moreover, GLU742 (4.60 Å), THR33 (3.73 Å), VAL36 (5.00 Å) exhibited π-anion, π-sigma and π-alkyl interaction with the phenyl ring of **2c**, respectively. Furthermore, ASP730 amino acid showed π-anion interaction with both the thiazole (3.92 Å) and phenyl ring (3.42 Å). However, π-alkyl interactions were also observed deeply inside the active moiety of the compound (thiazole ring) with MET746 (5.20 Å) and (phenyl ring) with LYS716 (5.24 Å). Subsequently, alkyl linkages were established through phenyl propane sulfonate ALA16 (4.44 Å) and PRO743 (4.58 Å) (Fig 4E and 4F).

The network of interactions between compounds **1d, 1h,** and **2c** in the active site of urease alongside the corresponding distance are shown in Table 6. The docking analysis exhibited that the most potent inhibitor **2c** that bears an *n*-propyl and a mesyl phenyl groups were responsible for the strongest binding interactions in the active site of urease. The *in vitro* results of the synthesized compounds possessing appreciable inhibitory activity against urease were further supported by their network of interactions in the catalytic site of the enzyme.

**Table 6:** Types of binding interaction, distance and atoms involved in interactions of compounds 1d, 1h, and 2c within 3LA4 receptor.

Compounds	Binding interactions			
	Ligands Atoms	Receptors Atoms	Interaction Type	Distance (Å)
**1d**	N9	TYR32	H-bond	2.66
	O24	LYS716	H-bond	2.94
	O25	LYS716	H-bond	3.22
	Imidazole ring	LYS709	π-cation	4.37
	Imidazole ring	GLU742	π-anion	4.49
	Imidazole ring	VAL744	π-alkyl	4.31
	Thiazole ring	GLU742	π-anion	4.09
	Thiazole ring	VAL744	π-alkyl	5.11
	Phenyl ring	VAL744	π-alkyl	5.39
	Phenyl ring	THR740	π-alkyl	4.75
	Phenyl ring	ASP730	π-anion	3.68
	Cyclohexane	VAL36	Alkyl bond	4.30
**1h**	O24	SER421	H-bond	2.94
	O25	SER421	H-bond	2.97
	Imidazole ring	LYS716	π-cation	4.06
	Thiazole ring	THR33	π-sigma	3.65
	Phenyl ring	TYR32	π-π T shaped	5.26
	Phenyl ring	ASP730	π-anion	4.71
	Phenyl ring	VAL744	π-alkyl	5.17
	Phenyl ring	MET746	π-alkyl	5.27
	Phenyl ring	LYS716	π-cation	4.20
**2c**	O30	TYR32	H-bond	3.19
	O30	LYS709	H-bond	3.29
	O25	VAL744	H-bond	3.28
	Thiazole ring	LYS716	π-cation	4.37
	Thiazole ring	MET746	π-alkyl	5.20
	Thiazole ring	ASP730	π-anion	3.92
	Imidazole ring	LYS716	π-cation	4.37
	Phenyl ring	GLU742	Pi-anion	4.60
	Phenyl ring	THR33	π-sigma	3.73
	Phenyl ring	VAL36	π-alkyl	5.00
	Phenyl ring	ASP730	π-anion	3.42
	Phenyl ring	LYS716	π-alkyl	5.24
	C27	ALA16	Alkyl bond	4.44
	C27	PRO743	Alkyl bond	4.58

**Fig 5:**
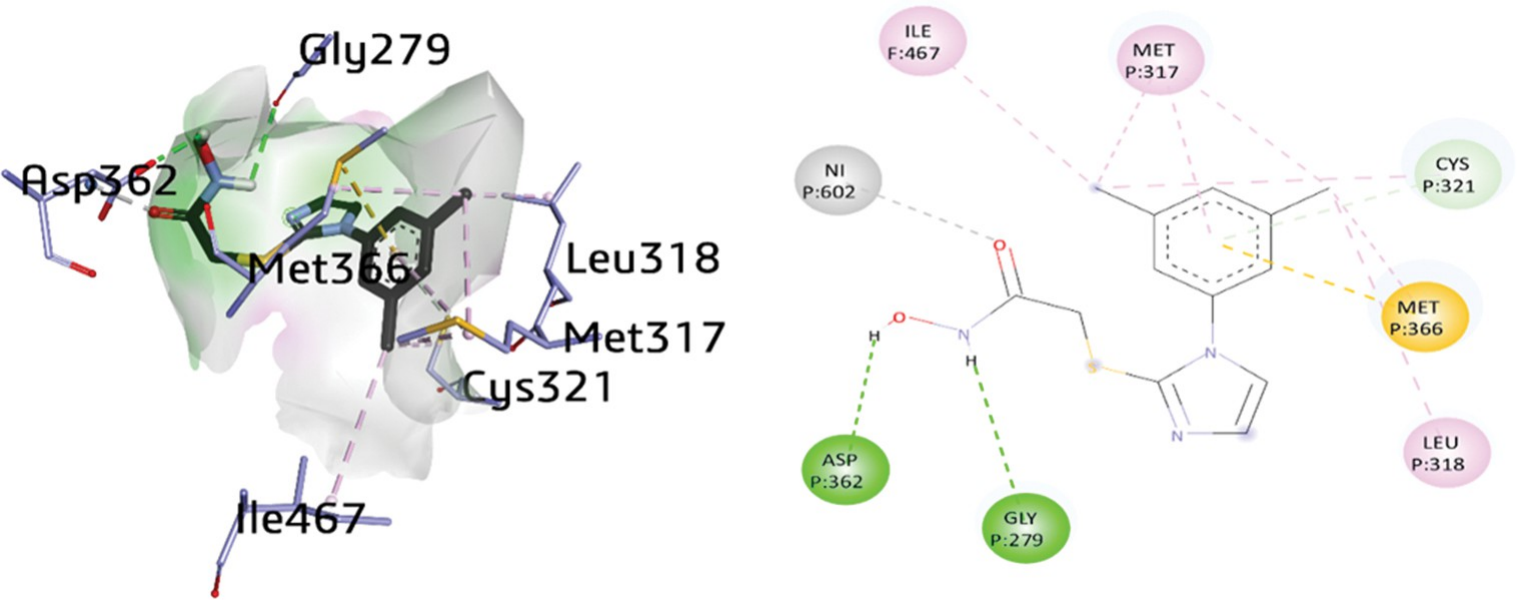
3D and 2D visualization of DJM (cognate ligand).

For the preformed docking studies, X-ray structure of *H*. *pylori* urease (PDB: ID 6ZJA) was designated due to its high crystallographic resolution (2.00 Å) [40]. Molecular docking studies of all the synthesized inhibitors were carried out against urease to probe the possible binding interactions. The study of the active pocket of urease co-crystal and all the potent inhibitors was exhibited in Fig 5. The orientation of the active compounds **1a**, **1d**, **1h**, **2d**, and **2f** along with the crystallographic inhibitor 2-[1-(3,5-dimethylphenyl)-1*H*-imidazol-2-yl]sulfanyl}-*N*-hydroxy acetamide (**DJM)** were presented in the active binding site of urease.

The active site of urease was enclosed by amino acid residues such as ASP362, GLY279, LEU318, MET366, CYS321, MET317, ILE467 and Ni 602. The cognate ligand exhibited two conventional hydrogen bonding with ASP362 (2.29 Å) and GLY279 (2.87 Å) and π-alkyl linkage with MET 317 (5.16 Å). Additionally, multiple alkyl interactions were noticed with LEU318 (4.29 Å), MET366 (4.46 Å), CYS321 (4.02 Å), MET317 (5.03 Å and 3.98 Å), and ILE467 (4.94 Å). Moreover, π-sigma and π-donor hydrogen bond were also observed with MET366 and CYS321 with distances of 4.28 Å and 3.76 Å, respectively. Nickel (Ni) was also linked with the O17 (2.21 Å) of the cognate ligand as shown in Fig 5.

**Fig 6:**
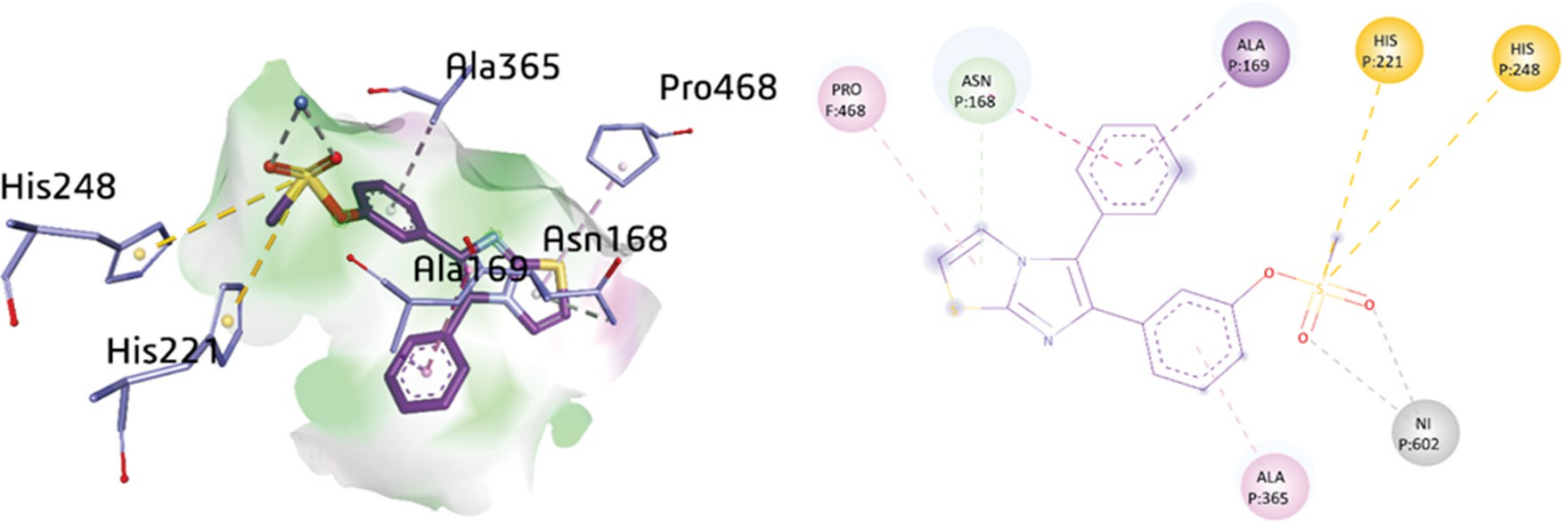
3D and 2D visualization of compound 1a.

Compound **1a** indicated various important interactions in the active site including π-alkyl between PRO468 (5.27 Å) and ALA365 (4.41 Å) with the thiazole ring and the phenyl ring, respectively. Consequently, π-sulfur bonds were observed between two amino acid residues, HIS248 (5.70 Å) and HIS221 (5.79 Å) and sulfur atom of sulfonate group. However, π-sigma bond was noticed between the ALA169 with a distance of 3.74 Å and the phenyl ring of the compound. Additionally, amide-π-stacking interaction was exhibited between the ASN168 (4.84 Å) and the phenyl ring. Moreover, C-H bond (4.01 Å) was depicted between the ASN168 and the thiazole ring. The O24 (2.56 Å) and O25 (2.81 Å) were bonded with the Ni 602 of compound **1a** as shown in Fig 6.

**Fig 7:**
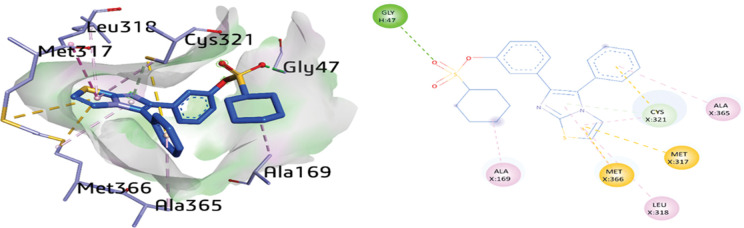
3D and 2D visualization of compound 1d.

Another potent compound, **1d** was docked inside the active pocket of urease and exhibited multiple interactions including amide-π stacking, π-alkyl and π-sulfur. The thiazole ring exhibited several interactions with various amino acid residues such as, amide-π stacking interactions with the MET317 (4.57 Å), while π-alkyl linkage was detected between LEU318 (5.21 Å) and CYS321 (4.76 Å), and π-sulfur with MET366 (3.87 Å) and MET317 (5.72 Å). Additionally, H-bonding was also noticed between the GLY47 with the distance of 3.14 Å and the sulfonate group. Furthermore, the imidazole ring exhibited π-alkyl and π-donor H-bonding with the amino acid residues MET366 (5.10 Å) and CYS321 (3.98 Å), respectively. Additionally, the amino acids CYS321 (5.14 Å) and ALA365 (5.01 Å) depicted π-sulfur and π-alkyl bond, respectively. Moreover, the amino acid ALA169 depicted an alkyl linkage with a distance of 3.60 Å with the cyclohexyl ring of compound **1d** as shown in Fig 7.

**Fig 8:**
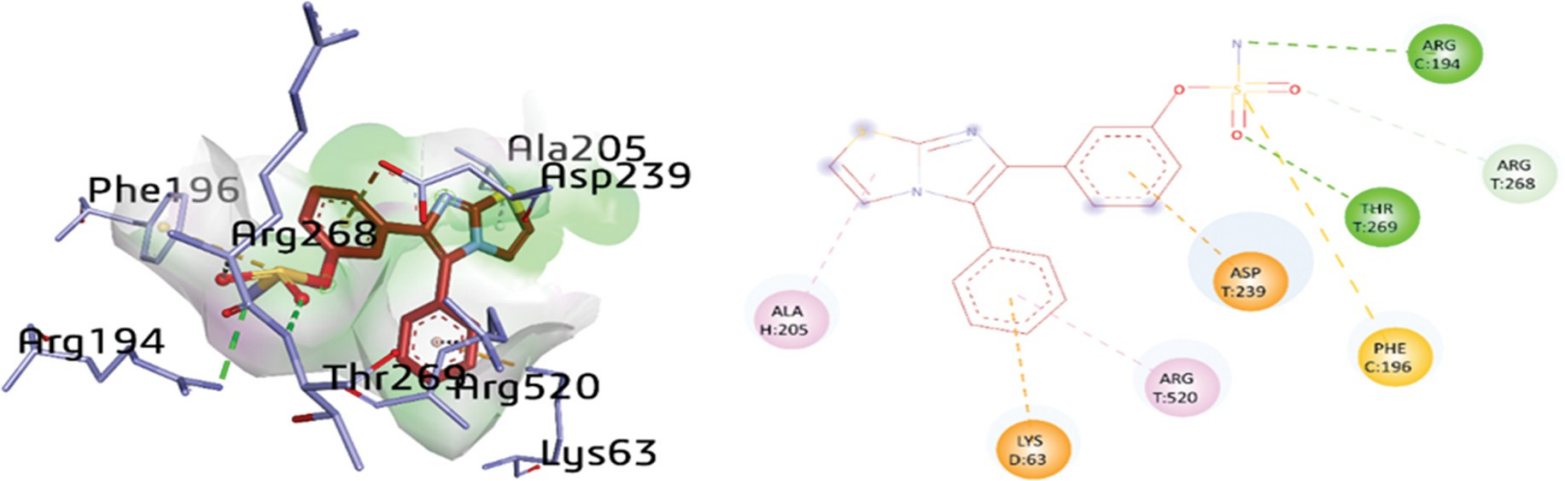
3D and 2D visualization of compound 1h.

The docking analysis of compound **1h** within the active pocket of urease depicted a few interactions with amino acid residues such as AGR194, THR269, ARG268, PHE196, ASP239, ARG520, LYS63, and ALA205. Upon exploration of docking results, ARG194 (3.13 Å) and THR269 (3.14 Å) were involved in H-bonding with the nitrogen and oxygen atoms of the sulfamate group, respectively. In addition, two π-alkyl linkages were also detected between the amino acids ALA205 (4.29 Å) and ARG520 (4.29 Å) and the thiazole and phenyl rings, respectively. Moreover, π-cation and π-anion interactions were also established between LYS63 (3.95 Å) and ASP239 (3.41 Å) and the phenyl ring of compound **1h**, respectively. However, a π-sulfur linkage (5.89 Å) was also depicted between the sulfur atom of the sulfamate moiety and PHE196, as shown in Fig 8.

**Fig 9:**
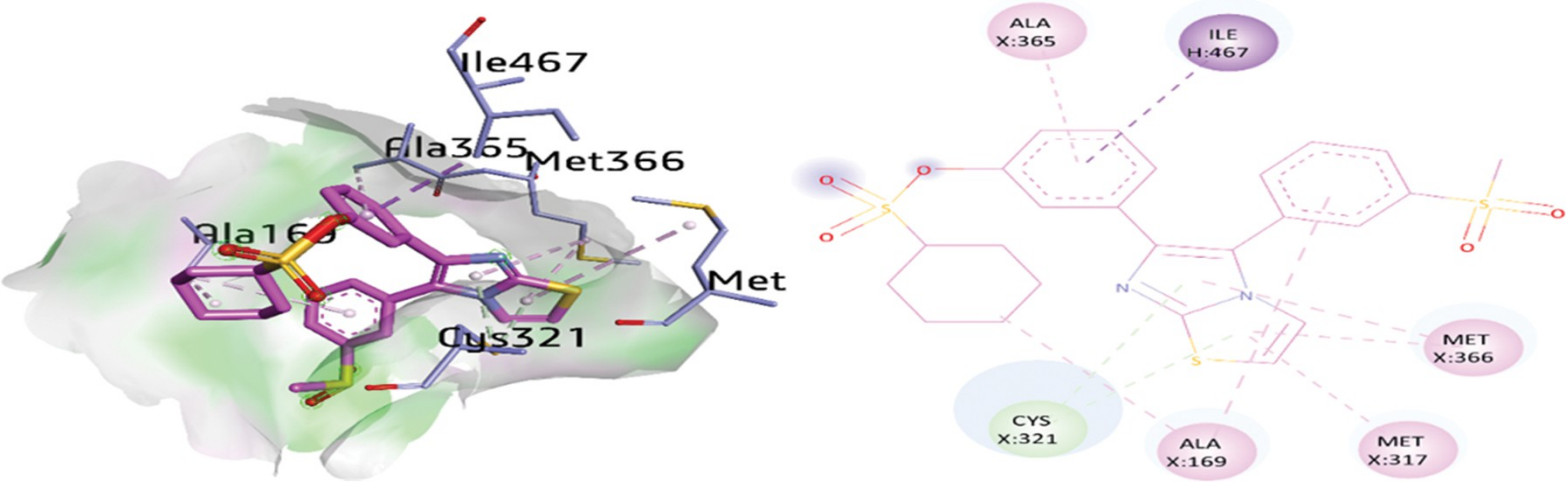
3D and 2D visualization of compound 2d.

The docking analysis of compound **2d** inside the active site of urease exhibited important interactions with ALA365, ILE467, MET366, MET317, ALA169 and CYS321. The imidazole ring exhibited π-alkyl and π-donor hydrogen bond with amino acid MET366 (5.15 Å) and CYS321 (3.81 Å), respectively. Furthermore, the thiazole ring also exhibited multiple interactions such as π-alkyl and π-donor H-bonding with various amino acids including MET366 (4.30 Å), MET317 (5.39 Å), and CYS321 (3.90 Å). On the other hand, ALA169 (4.93 Å) and ALA365 (5.32 Å) were involved in π-alkyl interactions with the phenyl ring of compound **2d**. Moreover, a π-sigma linkage was noticed between ILE467 (3.51 Å) and the phenyl ring as shown in Fig 9.

**Fig 10:**
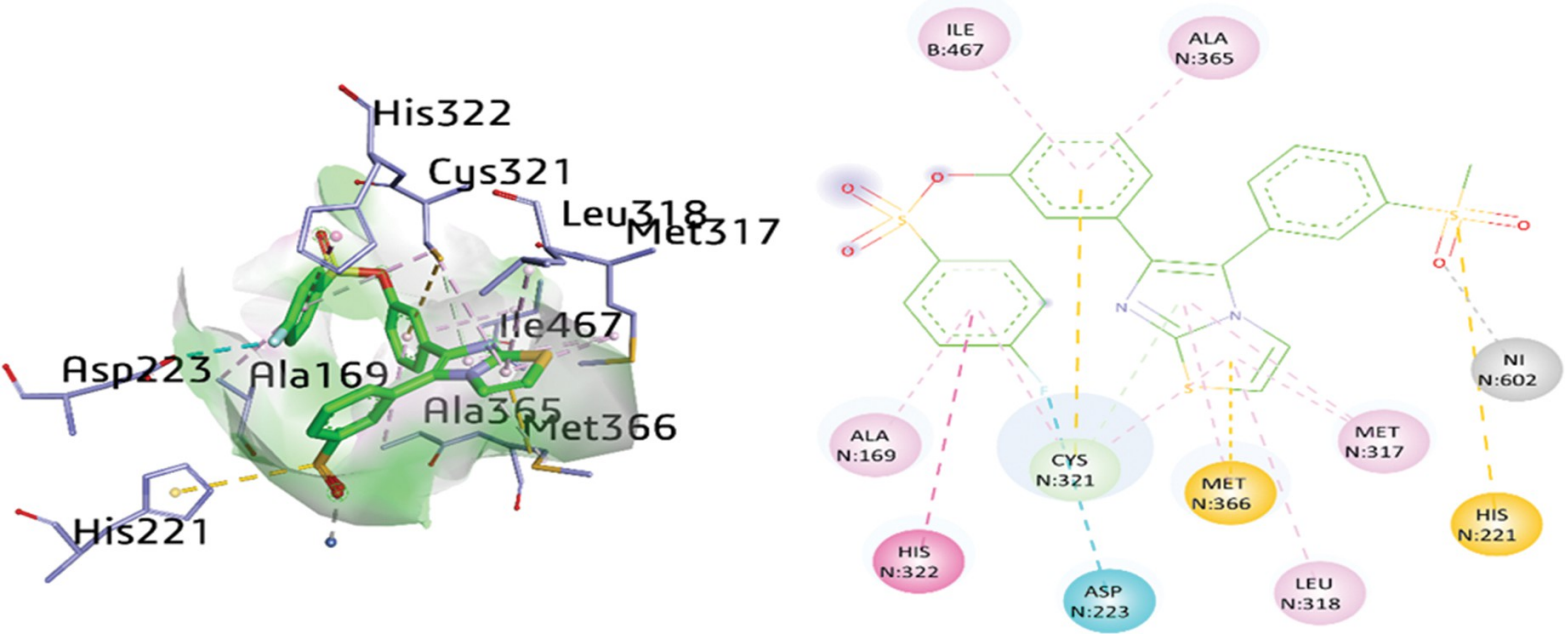
3D and 2D visualization of compound 2f.

The docking analysis of compound **2f** inside the active binding site of urease showed various interactions with several amino acids’ residues including ILE467, ALA365, ALA169, HIS322, CYS321, ASP223, MET366, LEU317, and HIS221. Ion-dipole interaction was detected between Ni (602) and the oxygen atom of the sulfonate group with a distance of 2.44 Å. The thiazole ring presented multiple interactions such as π-alkyl and π-sigma with the amino acid residues MET317 (5.24 Å), LEU318 (4.95 Å) and CYS321 (5.21 Å), and MET366 (3.73 Å). On the other hand, the imidazole ring also depicted π-alkyl interactions with MET317 (5.31 Å), MET366 (4.36 Å) and π-donor H-bonding with CYS328 (3.87 Å). CYS321 also revealed a π-sulfur linkage with the phenyl of a distance 5.23 Å, while π-π T-shaped interactions were also noticed between the other phenyl and HIS322 with a distance of 5.80 Å in compound **2f**. The 2 phenyl rings also exhibited multiple π-alkyl interactions with ALA169, CYS321, ALA365 and ILE467 with distances of 4.82 Å, 4.85 Å, 5.26 Å and 5.31 Å, respectively. Additionally, the amino acid ASP223 formed halogen (fluorine) bond with a distance of 3.68 Å. Moreover, HIS221 was involved in a π-sulfur bond with a distance of 4.79 Å in compound **2f** as shown in Fig 10. The different interactions between compounds **1a, 1d, 1h, 2d, 2f**, DJM and the protein (urease) are shown in Table 7.

**Table 7:** Types of binding interaction, distance and atoms involved in interactions of compounds 1a, 1d, 1h, 2d, 2f, and DJM.

Compounds	Binding interactions			
	Ligands Atoms	Receptors Atoms	Interaction Type	Distance (Å)
**1a**	O24	Ni602	Metal-acceptor	2.56
	O25	Ni602	Metal-acceptor	2.81
	S22	HIS221	π-sulfur	5.59
	S22	HIS248	π-sigma	5.70
	Thiazole ring	PRO468	π-alkyl	5.27
	Thiazole ring	ASN168	π-donor H-bond	3.39
	Phenyl ring	ASN168	Amide-π stacked	4.84
	Phenyl ring	ALA169	π-sigma	3.74
	Phenyl ring	ALA365	π-alkyl	4.41
**1d**	O24	GLY47	H-bond	3.14
	Imidazole ring	MET366	π-alkyl	5.10
	Imidazole ring	CYS321	π-donor H-bond	3.98
	Thiazole ring	CYS321	π-alkyl	4.76
	Thiazole ring	LEU318	π-alkyl	5.21
	Thiazole ring	MET366	π-sulfur	3.87
	Thiazole ring	MET317	π-sulfur	5.72
	Phenyl ring	CYS321	π-sulfur	5.14
	Phenyl ring	ALA365	π-alkyl	5.01
	Cyclohexane	ALA169	Alkyl	3.60
**1h**	N23	ARG194	H-bond	3.13
	O25	THR269	H-bond	3.14
	O24	ARG268	C-H bond	3.67
	Thiazole ring	ALA205	π-alkyl	4.29
	Phenyl ring	ARG520	π-alkyl	4.29
	Phenyl ring	LYS63	π-cation	3.95
	Phenyl ring	ASP239	π-anion	3.41
	S22	PHE196	π-sulfur	5.89
**2d**	Thiazole ring	MET317	π-alkyl	5.39
	Thiazole ring	MET366	π-alkyl	4.30
	Thiazole ring	CYS321	π-donor H-bond	3.90
	Imidazole ring	CYS321	π-donor H-bond	3.81
	Imidazole ring	MET366	π-alkyl	5.15
	Phenyl ring	ALA169	π-alkyl	4.93
	Phenyl ring	ALA365	π-alkyl	5.32
	Phenyl ring	ILE467	π-sigma	3.51
	Cyclohexane	ALA169	Alkyl	3.68
**2f**	O34	NI602	H-bond	2.44
	F31	ASP223	Halogen	3.68
	S32	HIS221	π-sulfur	4.79
	Thiazole ring	MET317	π-alkyl	5.24
	Thiazole ring	LEU318	π-alkyl	4.95
	Thiazole ring	CYS321	π-alkyl	5.15
	Thiazole ring	MET366	π-sulfur	3.73
	Imidazole ring	MET317	π-alkyl	5.31
	Imidazole ring	MET366	π-alkyl	4.36
	Imidazole ring	CYS321	π-alkyl	3.87
	Phenyl ring	ALA169	π-alkyl	4.82
	Phenyl ring	CYS321	π-alkyl	4.85
	Phenyl ring	ILE467	π-alkyl	5.31
	Phenyl ring	ALA365	π-alkyl	5.26
	Phenyl ring	CYS321	π-sulfur	5.23
	Phenyl ring	HIS322	π-π- T-shaped	5.80
**DJM**	H33	ASP362	H-bond	2.29
	H34	GLY279	H-bond	2.87
	O17	NI602	Metal-acceptor	2.21
	C1	ILE467	Alkyl	4.94
	C1	MET317	Alkyl	3.98
	C1	CYS321	Alkyl	4.02
	C5	MET317	Alkyl	5.03
	C5	MET366	Alkyl	4.46
	C5	LEU318	Alkyl	4.29
	Phenyl ring	CYS321	π-donor H-bond	3.76
	Phenyl ring	MET317	π-alkyl	5.16
	Phenyl ring	MET366	π-sulfur	4.28

### 3.9. Molecular dynamics simulation

In addition to molecular docking analysis, we have carried out a molecular simulation for the most potent inhibitors **1h** and **2c**. The molecular dynamic simulation of the urease in complex with compound **1h** was performed in an aqueous medium for 30 ns using the best docked pose bearing minimum binding energy. Different interactions (non-covalent) between compound **1h** and **2c** in the active binding site of enzyme (urease) were observed in a time dependent fashion.

**Fig 11:**
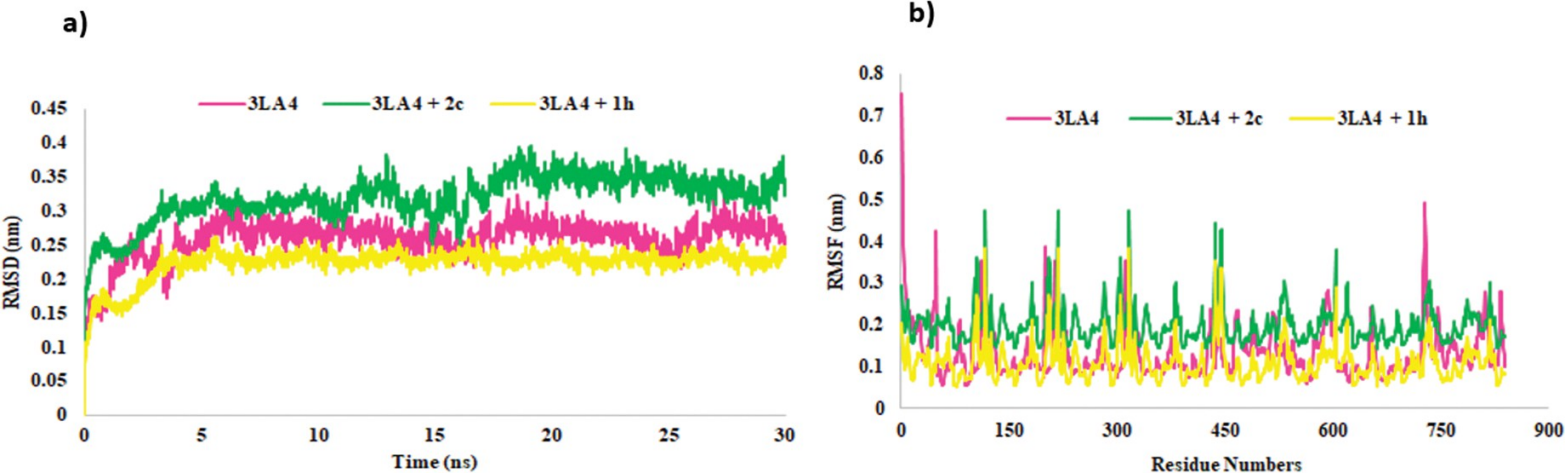
RMSD (11a) and RMSF (11b) of protein backbone (3LA4) during the simulation time of 30 ns in the presence and absence of **1h** and **2c**.

The RMSD results of MD showed the stability of protein alone and its complex with inhibitor. As shown in Fig 11A, comprehensive and reasonable simulations were noticed for the protein as well as **2c**-complex. Within the time span of 30 ns, stability was achieved and the number of deviations were found to be less. Overall, the stability was analyzed within the range of 0.2–0.4 nm and time frame of about 4 ns. The significant results were obtained with less deviation and more stability. The instability and to some extent deviations were noticed during 15–20 ns after which, the system attained its stability and consistency.

The root-mean square deviation (RMSD) results of MD simulation depicted the overall stability of apo protein and its complex with compound **1h**. As shown in Fig 11A, reasonable simulations were observed for the receptor and its complex with inhibitor. Within the range of 30 ns, stability was attained and little deviations were noticed. Overall, the stability of **1h** complex was estimated within the range of 0.1–0.25 nm and a time frame of about 5 ns. The structure of compound exhibited less deviation between 1–5 ns, after which the remaining system achieved its stability and consistency.

The root-mean square fluctuation (RMSF) shown in Fig 11B depicted that the protein alone and complex were flexible during the time span of the calculations. The system having both the protein and **2c**-complex showed fluctuations with a few amino acids previously reported. However, overall motion of systems was stable and no new fluctuations with amino acids were observed. The RMSF exhibited in Fig 11B depicted the flexibility of the protein in the presence or absence of inhibitor. As shown in Fig 11B, apo and holo protein depicted significant fluctuations with amino acid residues previously described. The results suggested the stability of the receptor-ligand complex (**1h**) as compared to the apo-protein alone. However, both systems exhibited stability and less fluctuations with amino acids.

**Fig 12:**
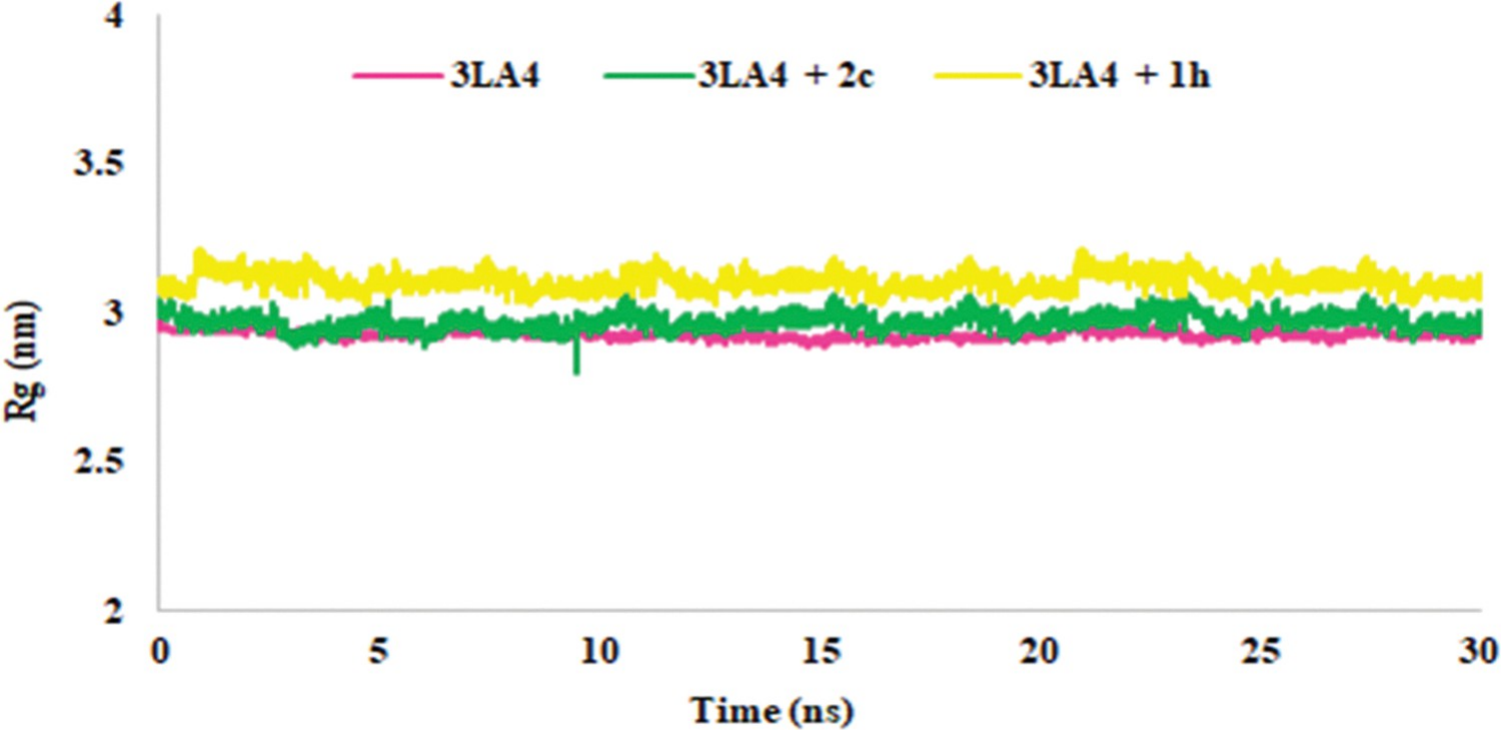
Rg in the presence and absence of 1h and 2c during simulation time (30 ns).

When the radius of gyration (Rg) was observed for the protein and complex, the compactness of the system during the simulation time depicted the folding and unfolding of structure. The protein in the presence and absence of inhibitor was almost compact and represented the average score of 2.9 nm with protein and 2.96 nm with **2c**-complex (Fig 12).

The radius of gyration was measured to establish the compactness of the system during simulation time to describe the folding and unfolding of structure. The results shown in Fig 12 exhibited the compactness of apo-protein in the presence or absence of inhibitor and revealed average score of 2.9 nm with receptor and 3.2 nm with **1h**-complex (Fig 12).

Therefore, the overall stability, fluctuations and compactness of protein and complex were investigated and found in agreement with the docking studies. This might be the reason for increased inhibition of **1h** and **2c** among all other compound towards Jack bean urease enzyme [62].

**Fig 13:**
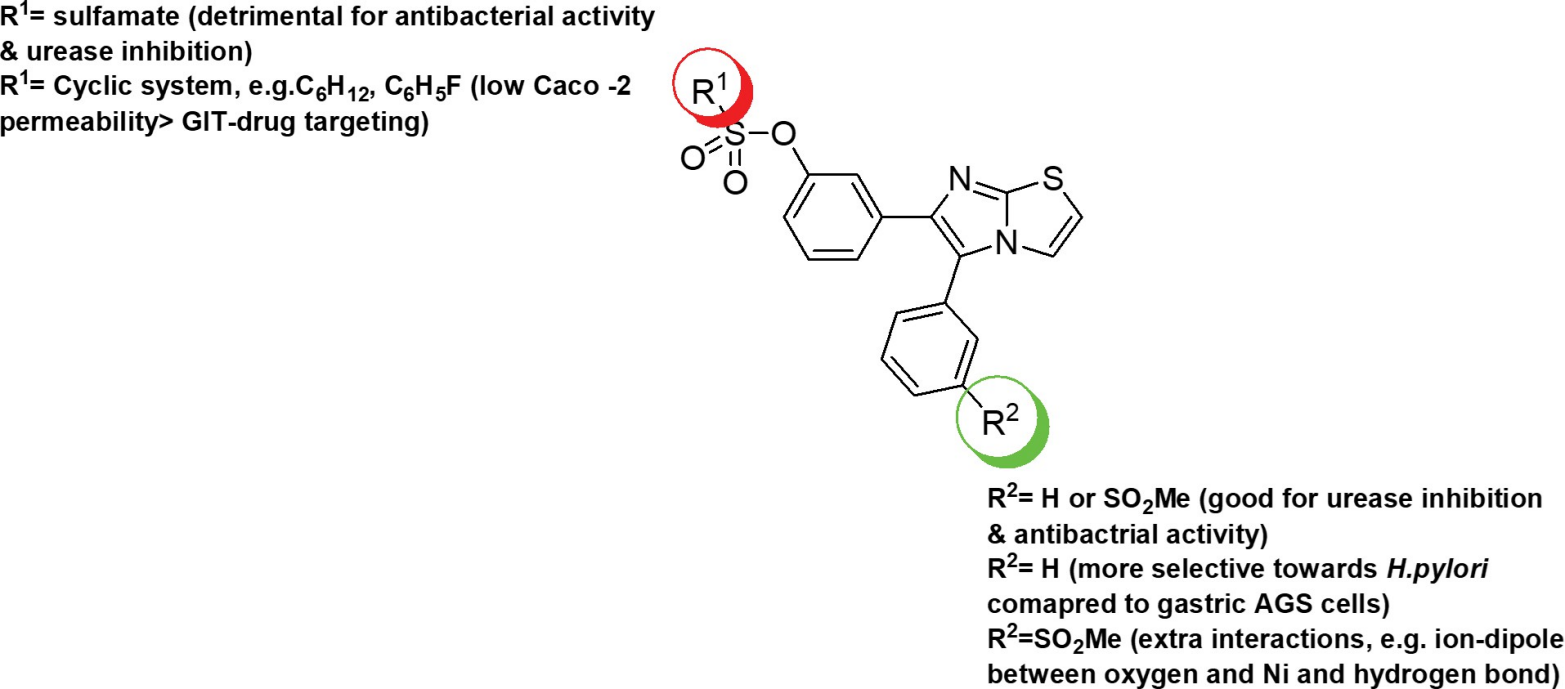
Summary of SAR of the target compounds (1a-1j and 2a-2j) based on both *in vitro* and *in silico* results.

Based on our *in vitro* and *in silico* findings, the favored key structural features (pharmacophores) that influence the bioactivity or/and physicochemical properties of this series were outlined in Fig 13. Concisely, sulfamates were not favorable for both, neither for anti-urease activity nor antibacterial activity, except for compound **1h**. Furthermore, incorporating cyclic substituents, either cyclohexyl or 4-fluorobenzene at the sulfonate group has reduced Caco-2 permeability which permits to achieve probable GIT-drug targeting. Insignificant difference was revealed upon substituting either phenyl or 3-mesyl phenyl group a position-5 of the imidazothiazole nucleus. However, the computational findings presented the contribution of the oxygen atoms of the mesyl group in the formation of ion-dipole interaction with the cation binding moiety, Ni (compound **2f**), in addition it contributes to form additional hydrogen bond such as in compound **2c**. Additional interactions can possibly increase the affinity of the compound towards the target, urease, and consequently enhancing its potency.

## 4. Conclusions

In summary, recognizing the importance of sulfonate and sulfamate as intricate synthons for the discovery of drug candidates, a new series of sulfonate and sulfamate derivatives were developed. Electron-rich and electron-poor functional groups at imidazo[2,1-*b*]thiazole ring afforded a great opportunity for creating H-bonding interactions with several amino acid residues of urease including TYR32, LYS716, SER421, LYS709, and VAL744. The developed compounds **1a-1j** and **2a-2j** exhibited significant inhibitory activities towards urease as compared to the thiourea positive control. Compound **2c** (a competitive inhibitor of urease with IC_50_ value of 2.94 ± 0.05 μM) exhibited an 8-fold stronger inhibition against urease compared to thiourea. Consequently, *in silico* analysis of compound **2c** exhibited multiple important interactions such H-bonding, π-anion, π-cation, π-alkyl and alkyl interactions with different amino acids including TYR32, LYS709, VAL744, LYS716, MET746, ASP730, LYS716, GLU742, THR33, VAL36, ASP730, ALA16 and PRO743. Molecular dynamic simulation further confirmed the overall protein stability and its complex with compound **1h** and **2c**.

Moreover, the developed compounds were subjected to anti-helicobacter testing, which revealed submillimolar MIC and IC_50_ values. Among constructed series, the five most potent imidazothiazole compounds (**1a, 1d, 1h, 2d**, and **2f)** were subjected to further studies that include investigating their cytotoxicity against AGS gastric cancer cells. Compound **1d** exhibited the highest selectivity, with an SI value of 6.05. Additionally, selectivity testing was also conducted against urease-positive bacteria (*H*. *pylori*) and urease-negative bacteria (*E*. *coli*). Interestingly, all the five compounds lacked inhibitory activity against *E*. *coli* which concludes the selectivity of those imidazothiazole derivatives against urease-positive bacteria *H*. *pylori*. Furthermore, the potential oral absorption of the most promising compounds was evaluated by exploiting Caco-2 permeability studies. Our findings revealed that compounds **1d, 2d**, and **2f** are suitable agents to target the GIT due to their low permeability, and hence having low oral absorption thereby eliminating systemic side effect(s). *In vitro* and *in silico* results led us to understand the indicative parameters important for SAR, sulfonate side chain was more preferred for anti-urease and antibacterial activity. Substitution of cyclic groups (cyclohexane or 4-fluorobenzene) at the sulfonate moiety lowers Caco-2 permeability which aids to achieve GIT-drug targeting. Having a phenyl or 3-mesyl phenyl group at position-5 of the imidazothiazole scaffold did not show any significant difference in the *in vitro* studies, however the *in silico* studies showed that the oxygen atoms of the 3-mesyl group can reveal an extra interactions, such as hydrogen bond with a nearby amino acid residue or ion-dipole interaction with the metal cation, Ni.

Overall, the cyclohexanesulfonate derivative **1d** is the most promising among this series of compounds. It showed promising activity and high SI against *H*. *pylori*, as well as poor Caco-2 A-B permeability. It can be utilized as a promising hit for development of target antimicrobial agents for management of *H*. *pylori* infections.

## Supporting information

S1 File The supplementary file contains LC-MS/^1^H NMR/^13^C NMR charts, dose-response curves against urease, as well as antibacterial and cytotoxicity results of the target compounds. (DOCX)

S1 Graphical abstract (DOCX)
