# Supplementary material for: Design and synthesis of novel anti-urease imidazothiazole derivatives with promising antibacterial activity against Helicobacter pylori
Source: PLoS One. 2023 Jun 2;18(6):e0286684. doi: 10.1371/journal.pone.0286684 (PMC10237672; doi:10.1371/journal.pone.0286684)
Supplement: S1 File — (DOCX) [file pone.0286684.s001.docx]

**Design and synthesis of novel anti-urease imidazothiazole derivatives with promising antibacterial activity against *Helicobacter pylori***

**Afnan I. Shahin ^1^, Sumera Zaib ^2,*^, Seyed-Omar Zaraei ^1^, Reena A. Kedia ^1^, Hanan S. Anbar ^3^, Muhammad Tayyab Younas ^2^, Taleb H. Al-Tel ^1,4^, Ghalia Khoder ^1,5,*^, and Mohammed I. El-Gamal ^1,4,6,*^**

^1^ Research Institute for Medical and Health Sciences, University of Sharjah, Sharjah 27272, United Arab Emirates

^2^ Department of Basic and Applied Chemistry, Faculty of Science and Technology, University of Central Punjab, Lahore-54590, Pakistan

^3^ Department of Clinical Pharmacy and Pharmacotherapeutics, Dubai Pharmacy College for Girls, Dubai 19099, United Arab Emirates

^4^ Department of Medicinal Chemistry, College of Pharmacy, University of Sharjah, Sharjah 27272, United Arab Emirates

^5^ Department of Pharmaceutics and Pharmaceutical Technology, College of Pharmacy, University of Sharjah, Sharjah 27272, United Arab Emirates

^6^ Department of Medicinal Chemistry, Faculty of Pharmacy, Mansoura University, Mansoura 35516, Egypt

^*^Corresponding authors. E-mail addresses: [drmelgamal2002@gmail.com](mailto:drmelgamal2002@gmail.com) & [malgamal@sharjah.ac.ae](mailto:malgamal@sharjah.ac.ae) & [drmelgamal@mans.edu.eg](mailto:drmelgamal@mans.edu.eg) (M.I. El-Gamal); [gkhoder@sharjah.ac.ae](mailto:gkhoder@sharjah.ac.ae) (G. Khoder); [sumera.biochem@gmail.com](mailto:sumera.biochem@gmail.com) ; [sumera.zaib@ucp.edu.pk](mailto:sumera.zaib@ucp.edu.pk) (S. Zaib).

| **Content** | **Page No.** |
| --- | --- |
| LC-MS, ^1^H NMR, and ^13^C NMR charts of compounds **1a-1j** and **2a-2j** | S2-S41 |
| Dose-response curves of compounds **1a-1j** and **2a-2j** against urease enzyme | S42-S43 |
| Antibacterial and cytotoxicity results | S44-S48 |

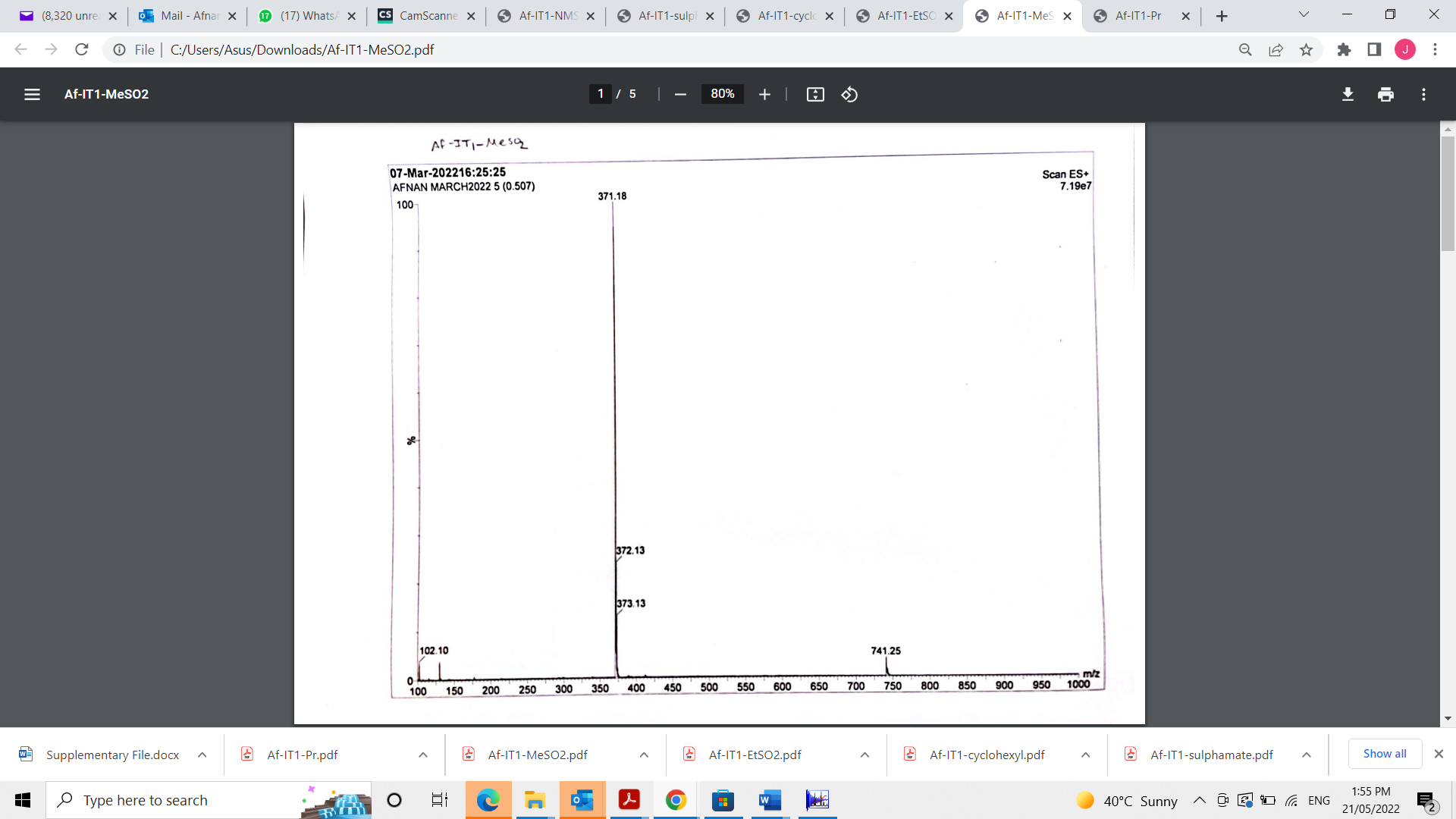


**Figure S1.** LC-MS chart of compound **1a**.


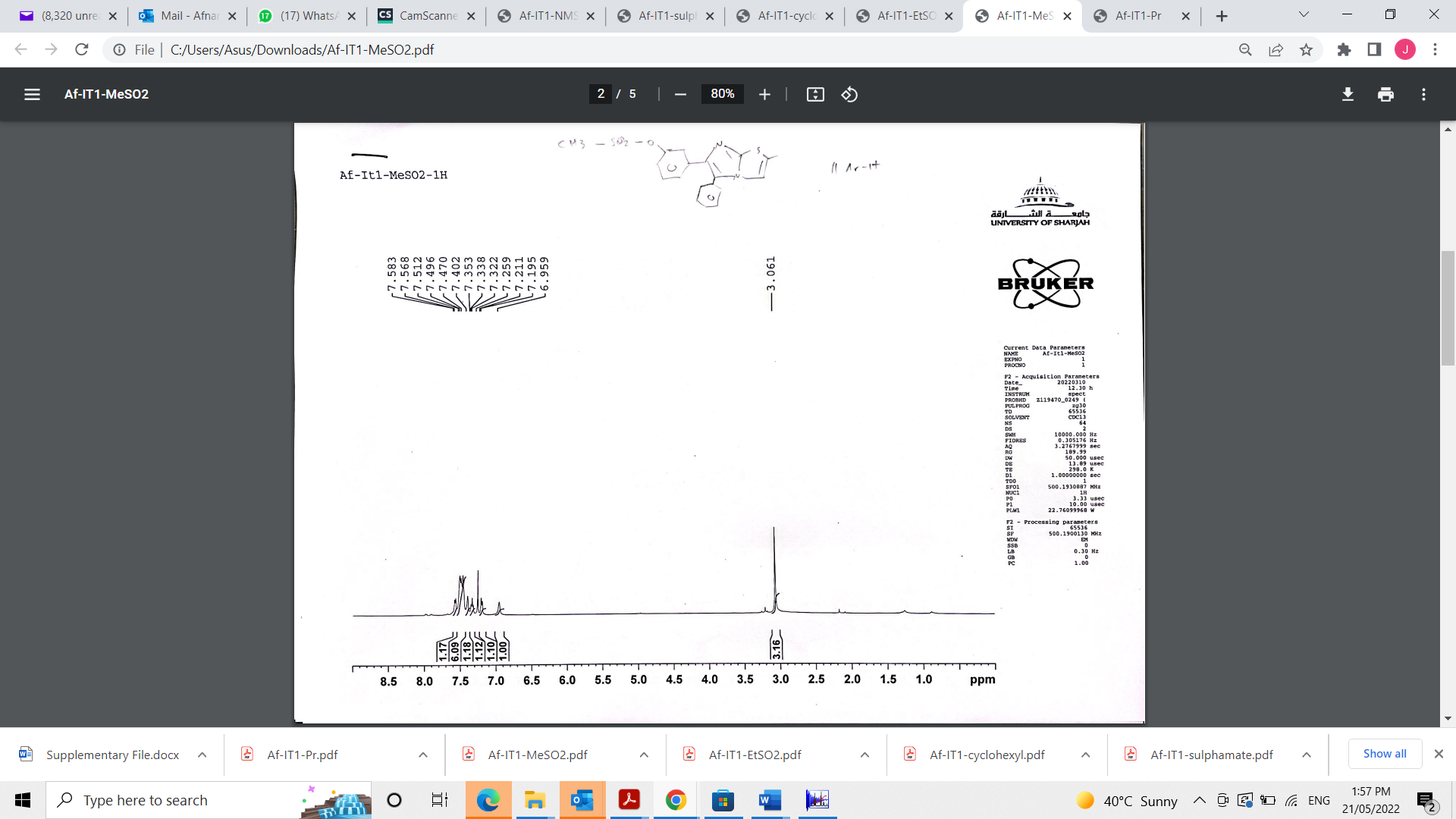


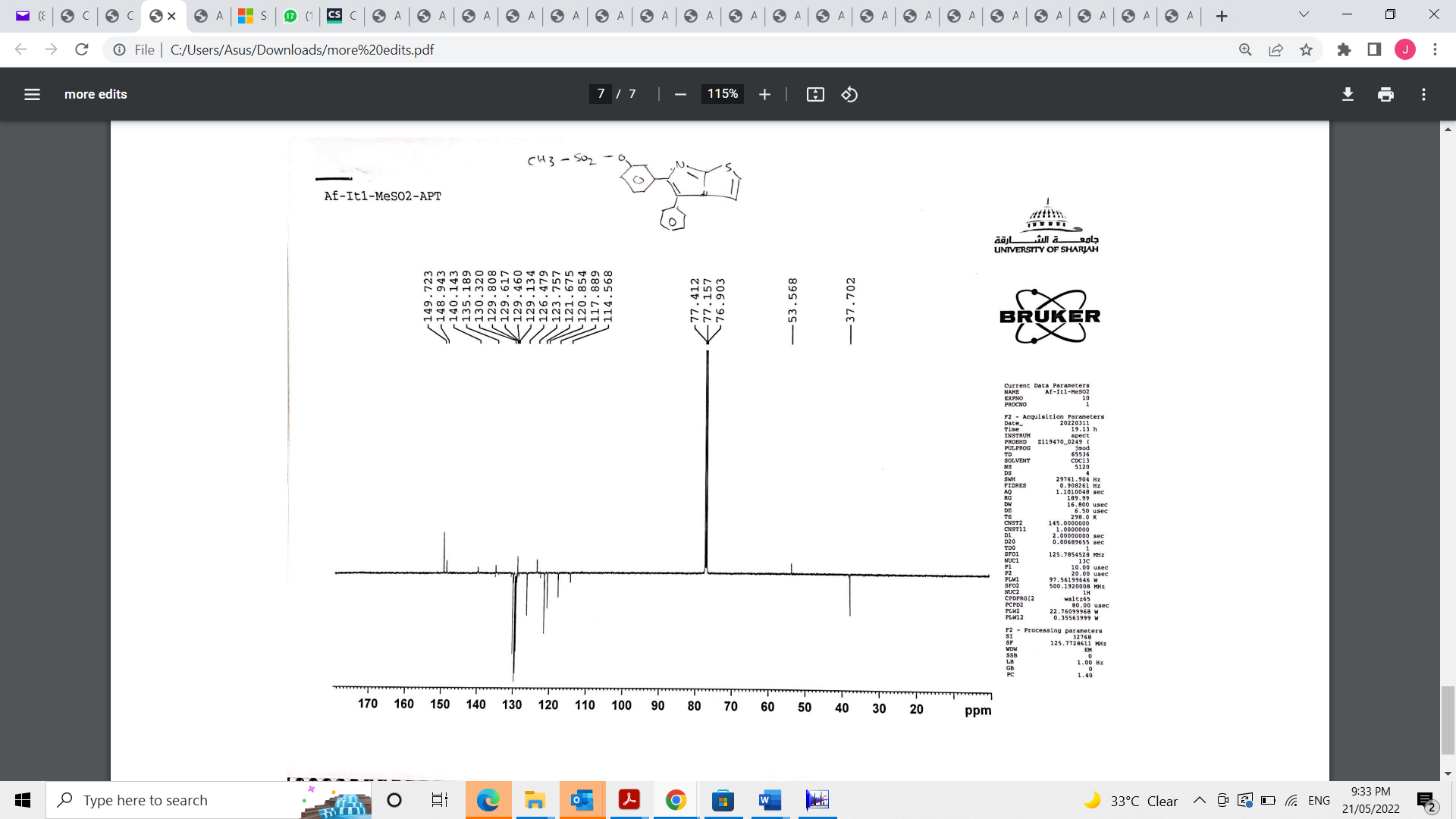


**Figure S2.** ^1^H NMR and ^13^C NMR charts of compound **1a**.

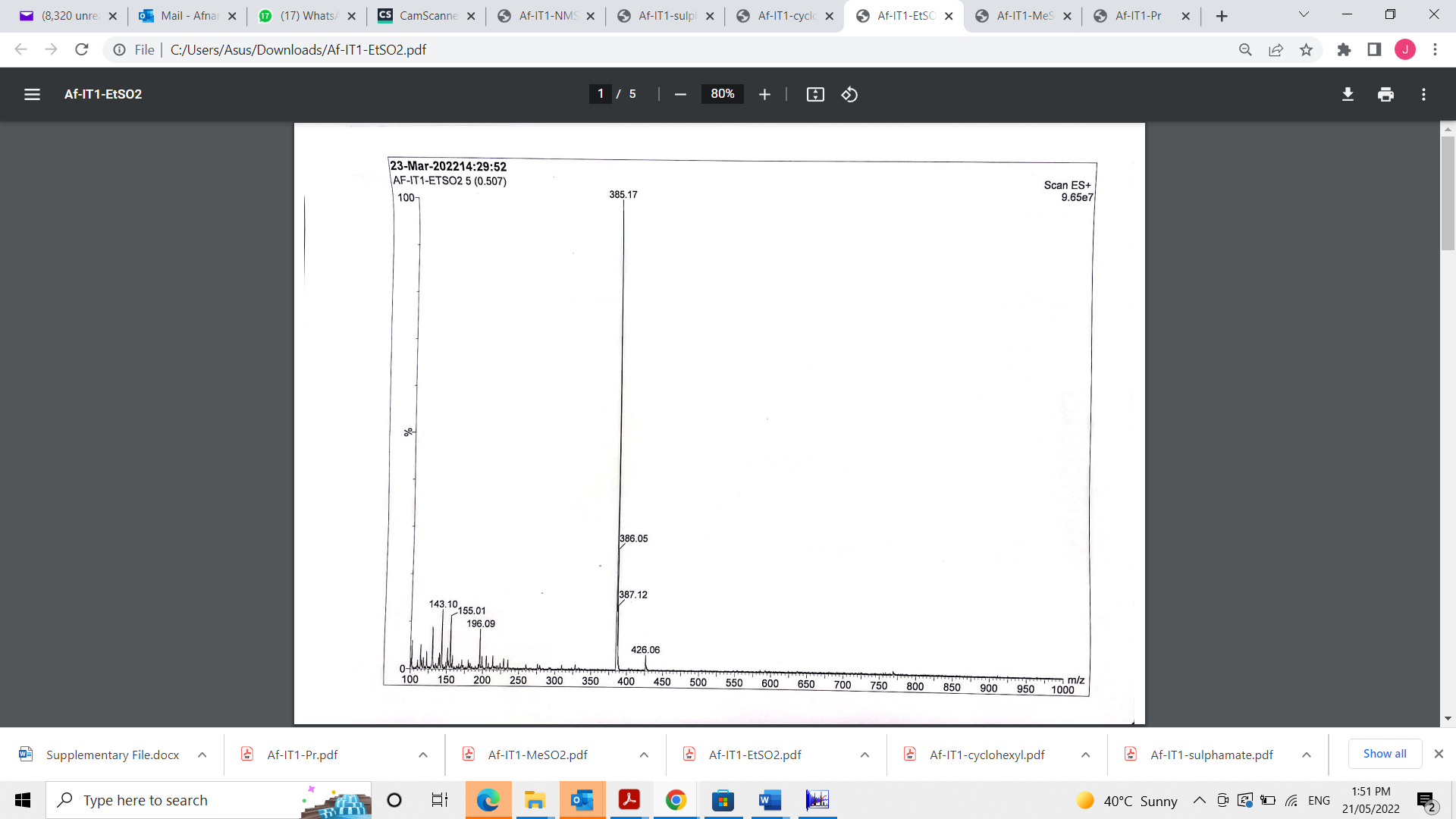


**Figure S3.** LC-MS chart of compound **1b**.


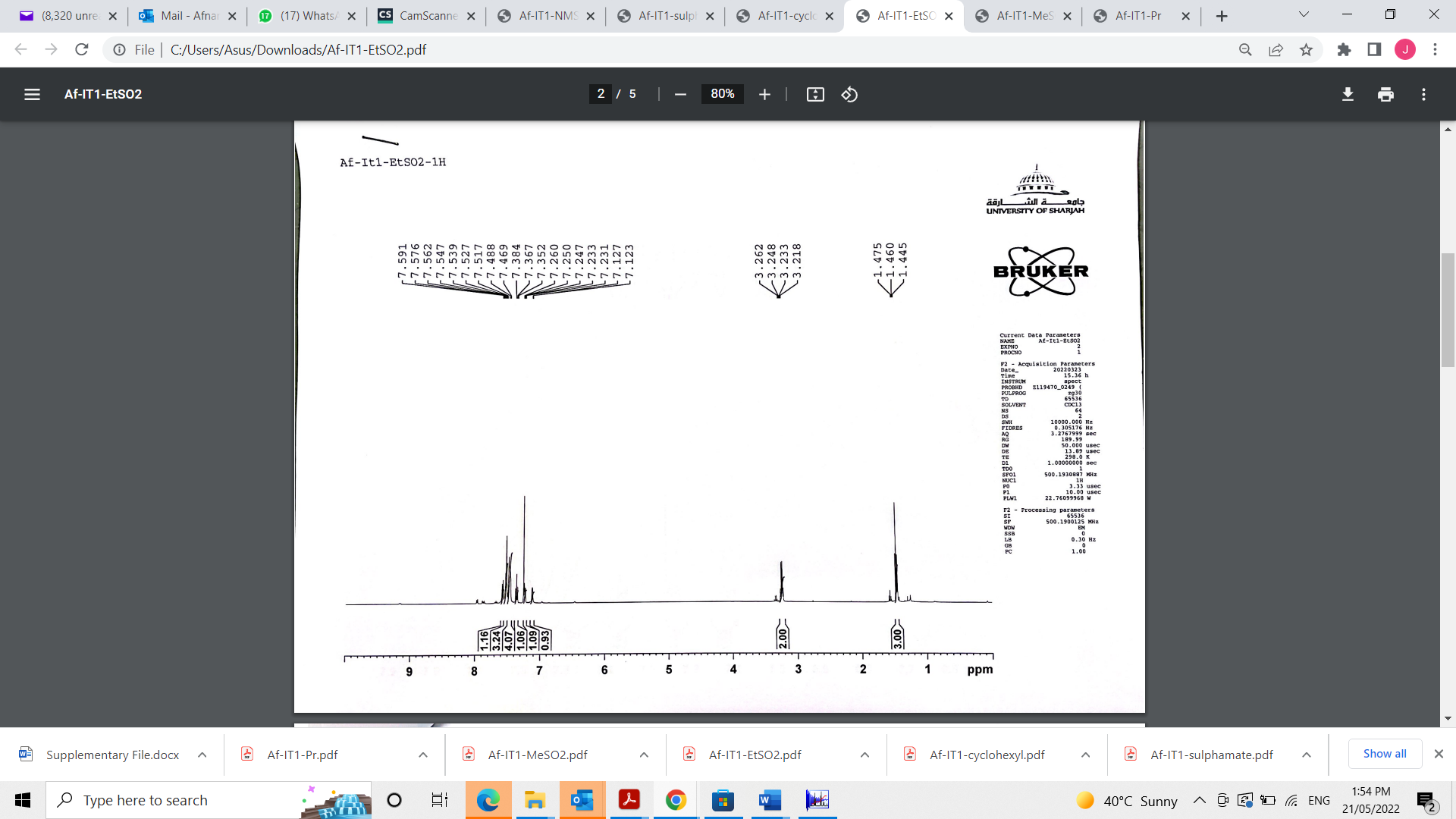


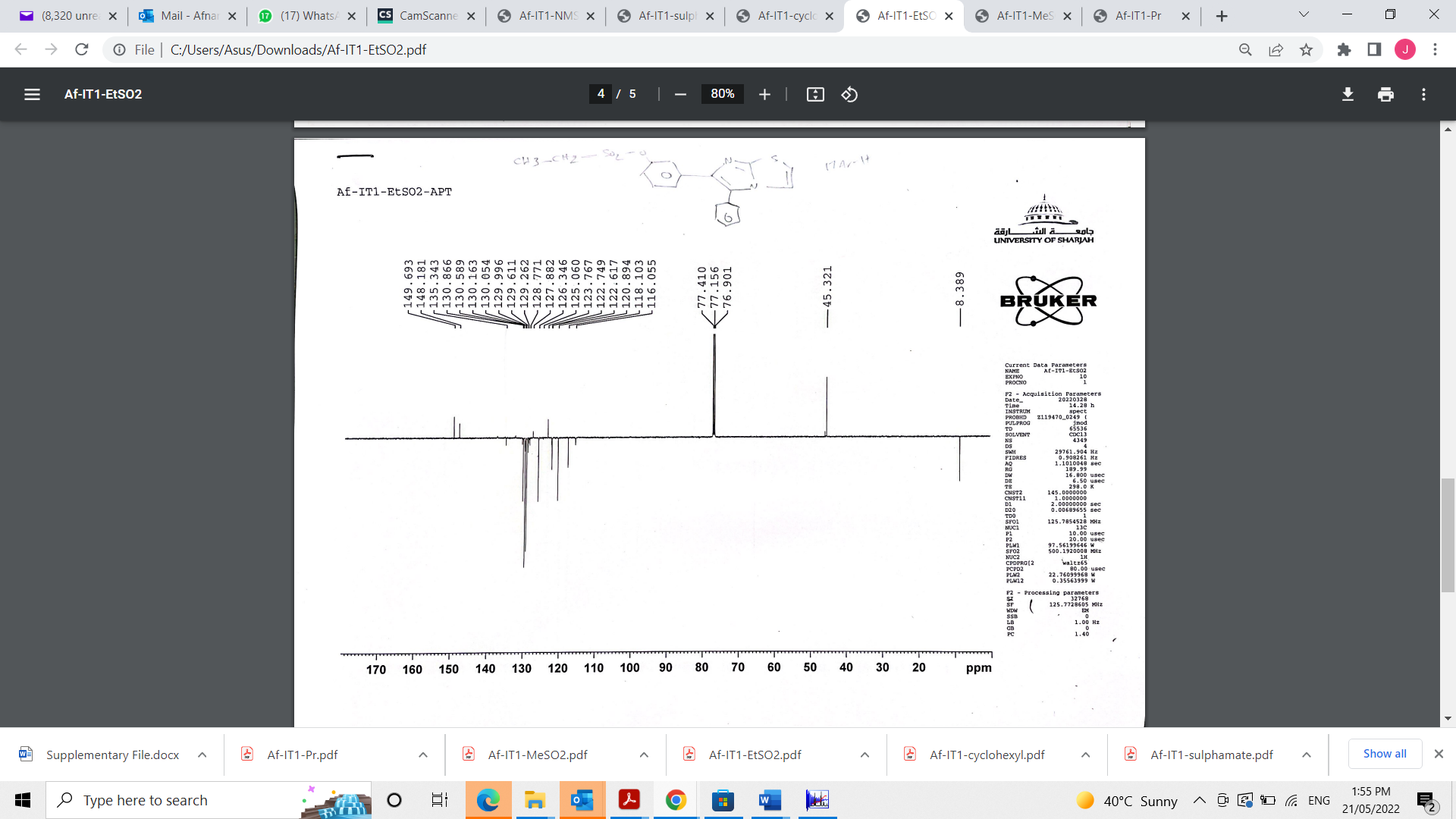


**Figure S4.** ^1^H NMR and ^13^C NMR charts of compound **1b**.

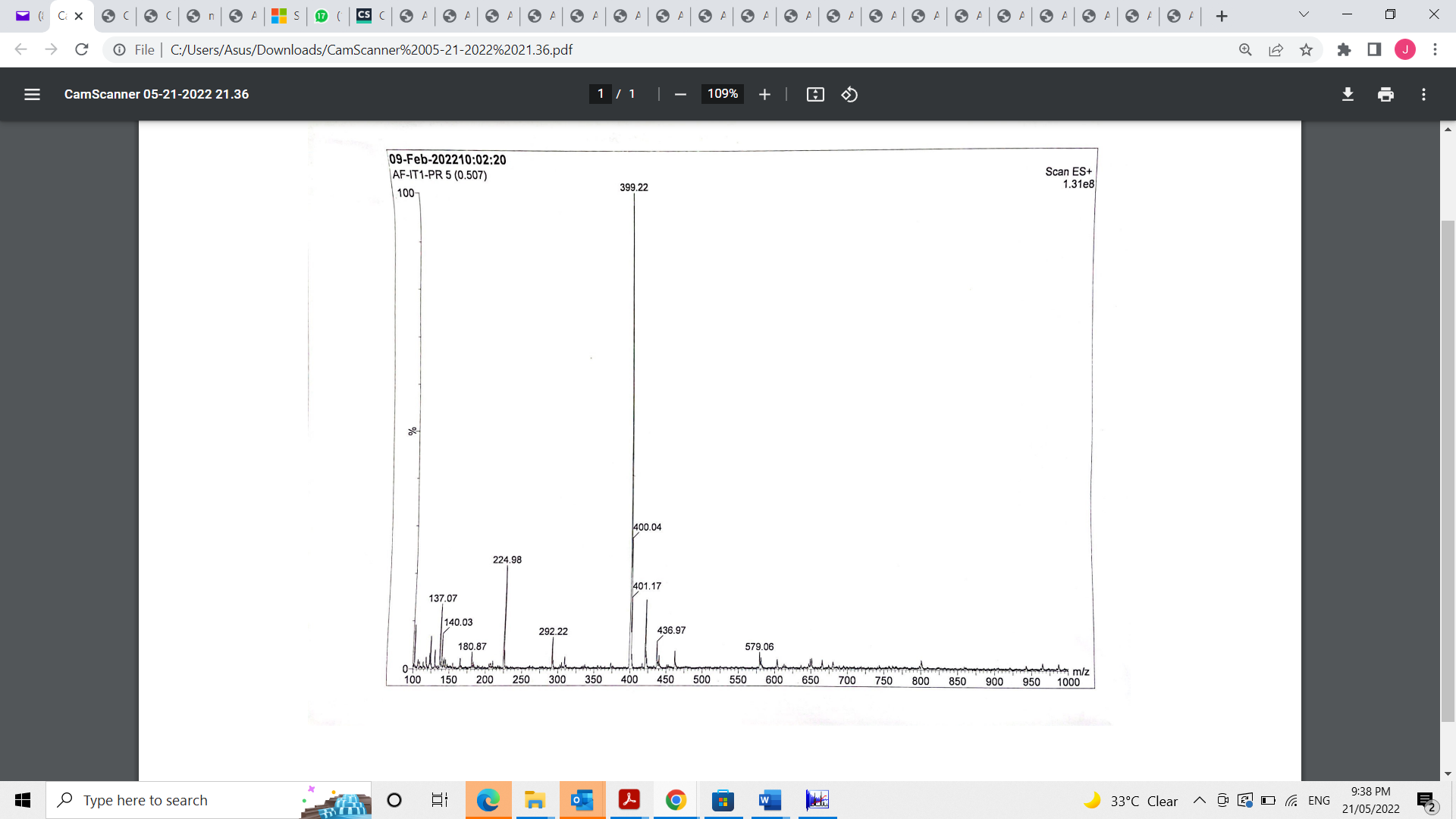


**Figure S5.** LC-MS chart of compound **1c**.

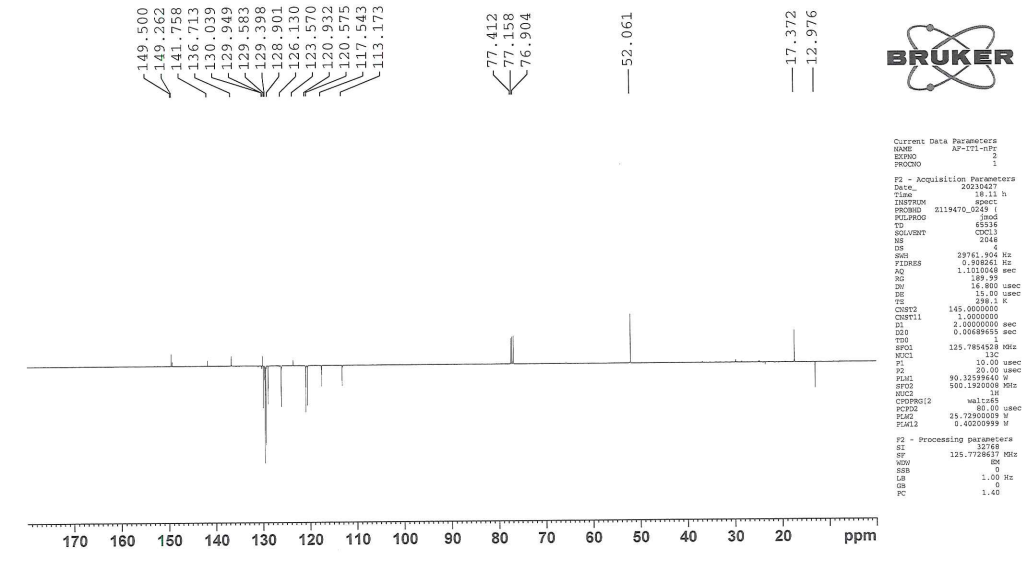


**Figure S6.** ^1^H NMR and ^13^C NMR charts of compound **1c**.

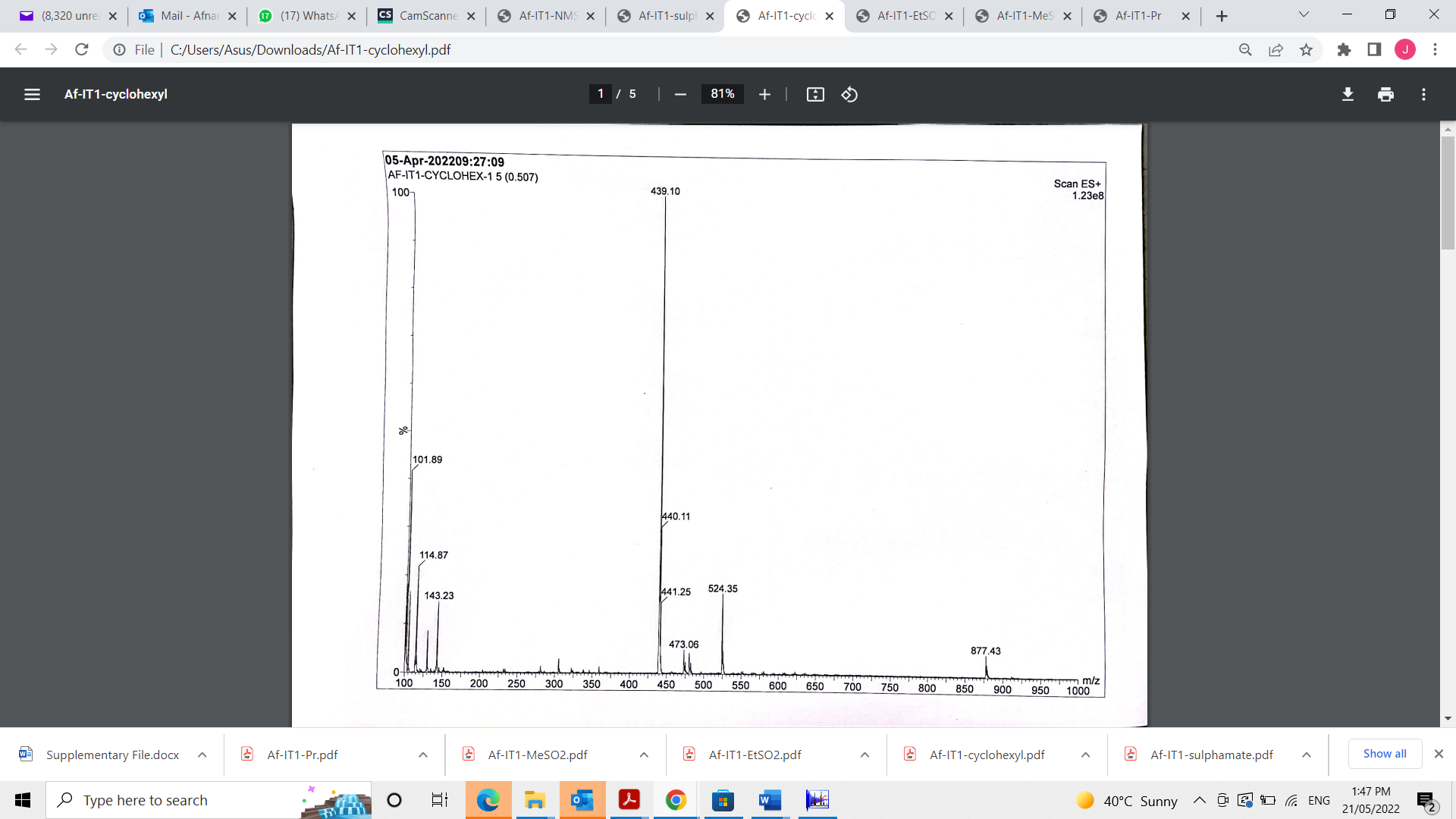


**Figure S7.** LC-MS chart of compound **1d**.


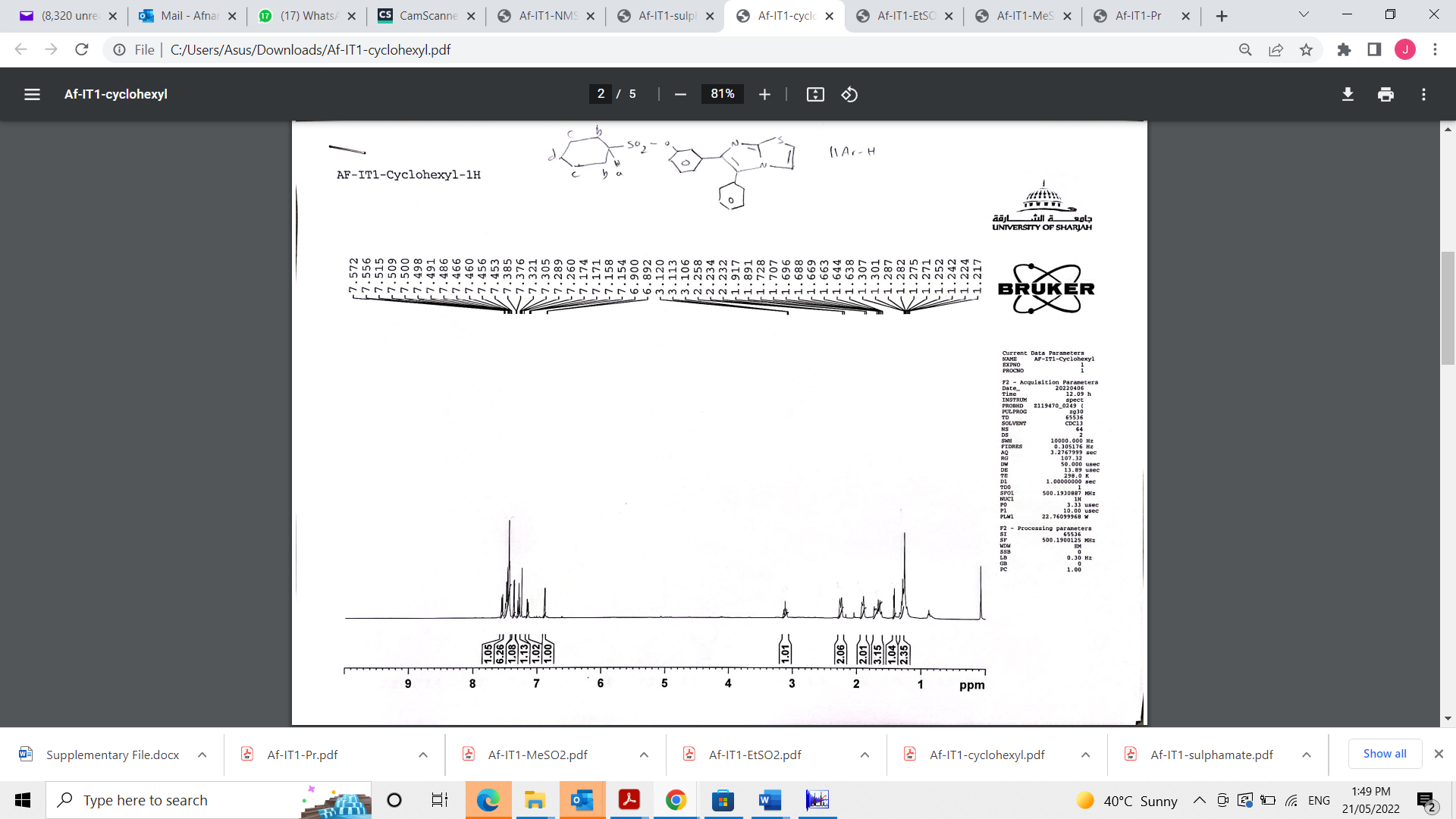

**Figure S8.** ^1^H NMR and ^13^C NMR charts of compound **1d**.

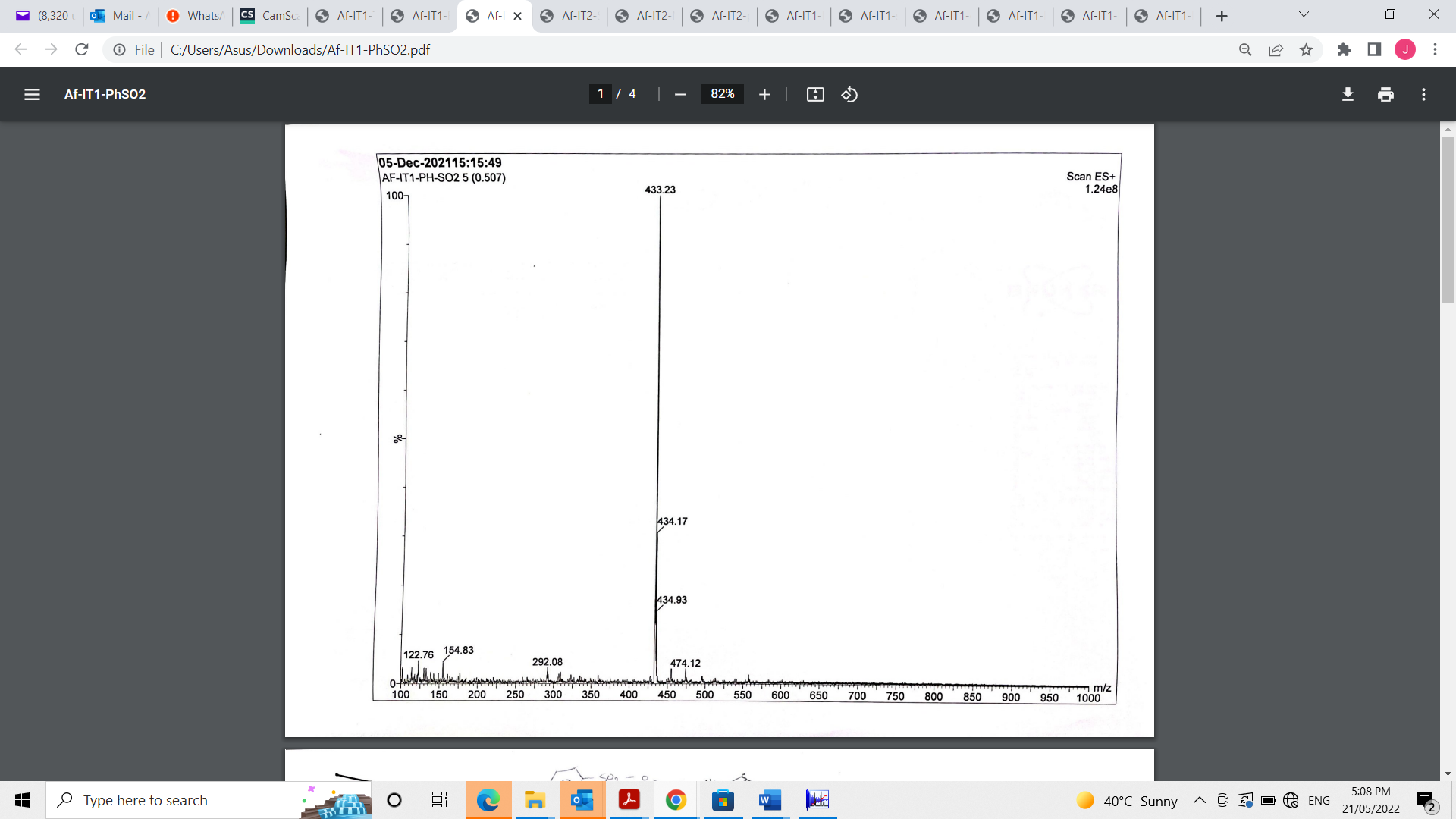


**Figure S9.** LC-MS chart of compound **1e**.


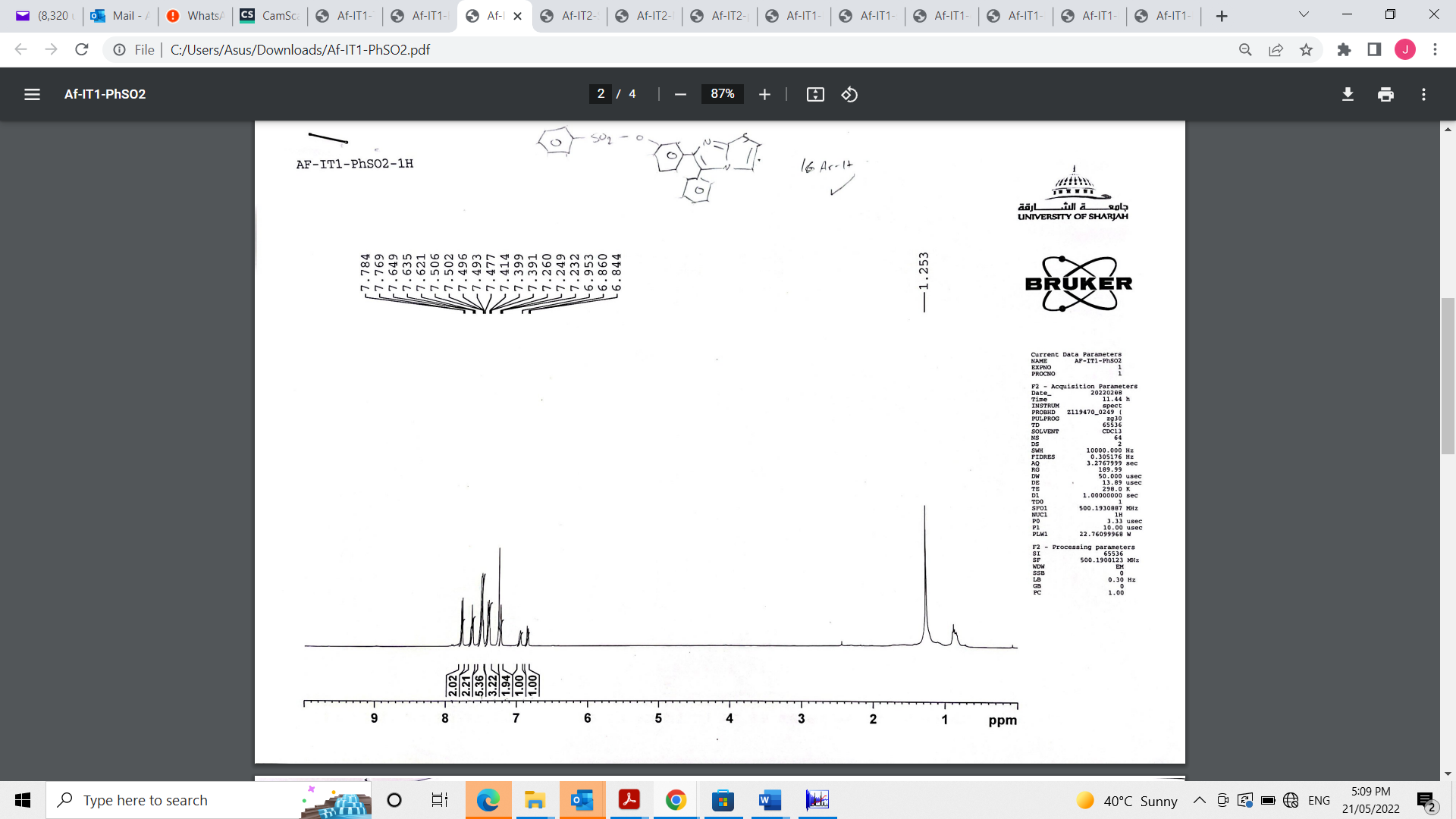


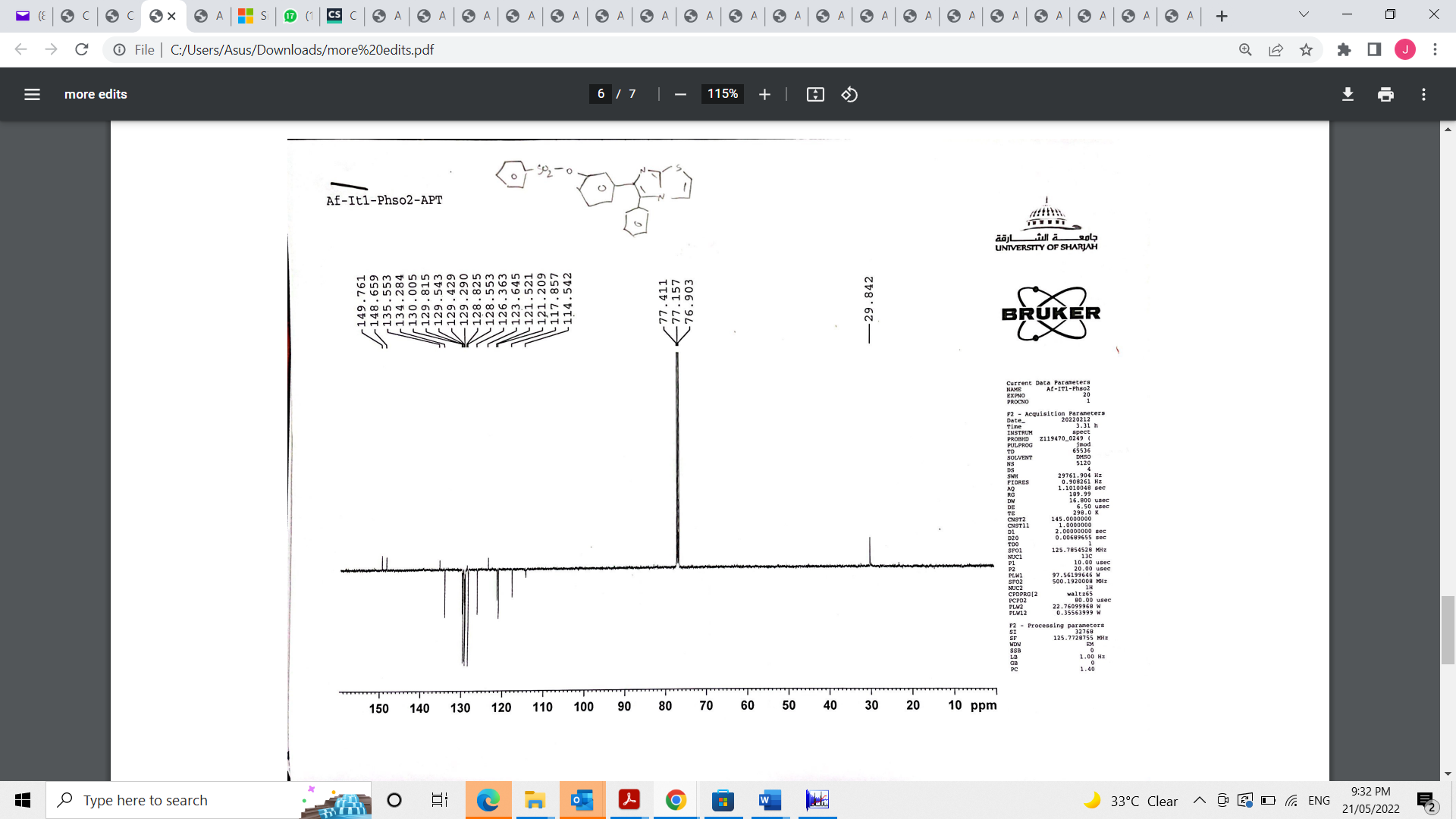


**Figure S10.** ^1^H NMR and ^13^C NMR charts of compound **1e**.

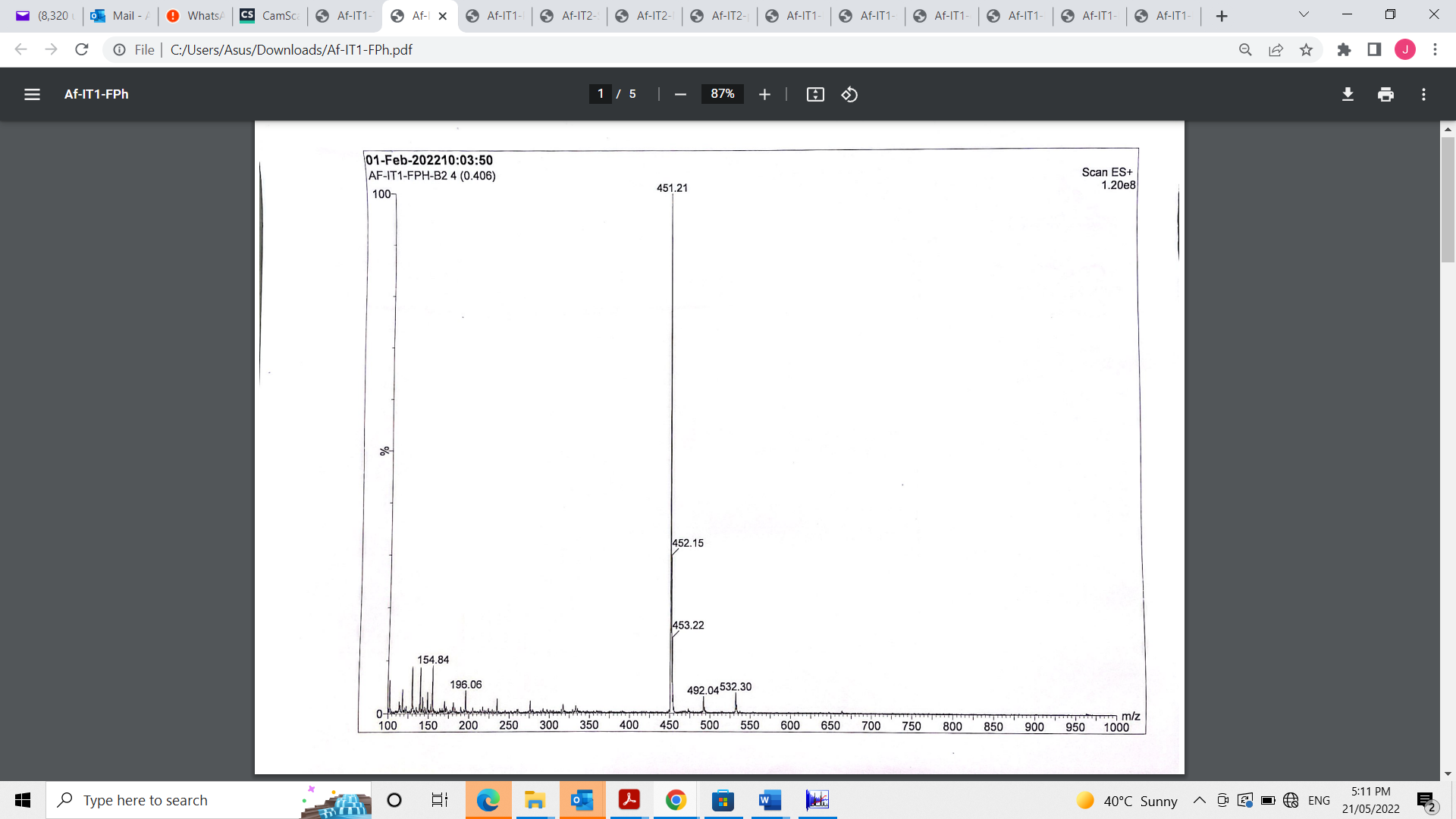


**Figure S11.** LC-MS chart of compound **1f**.


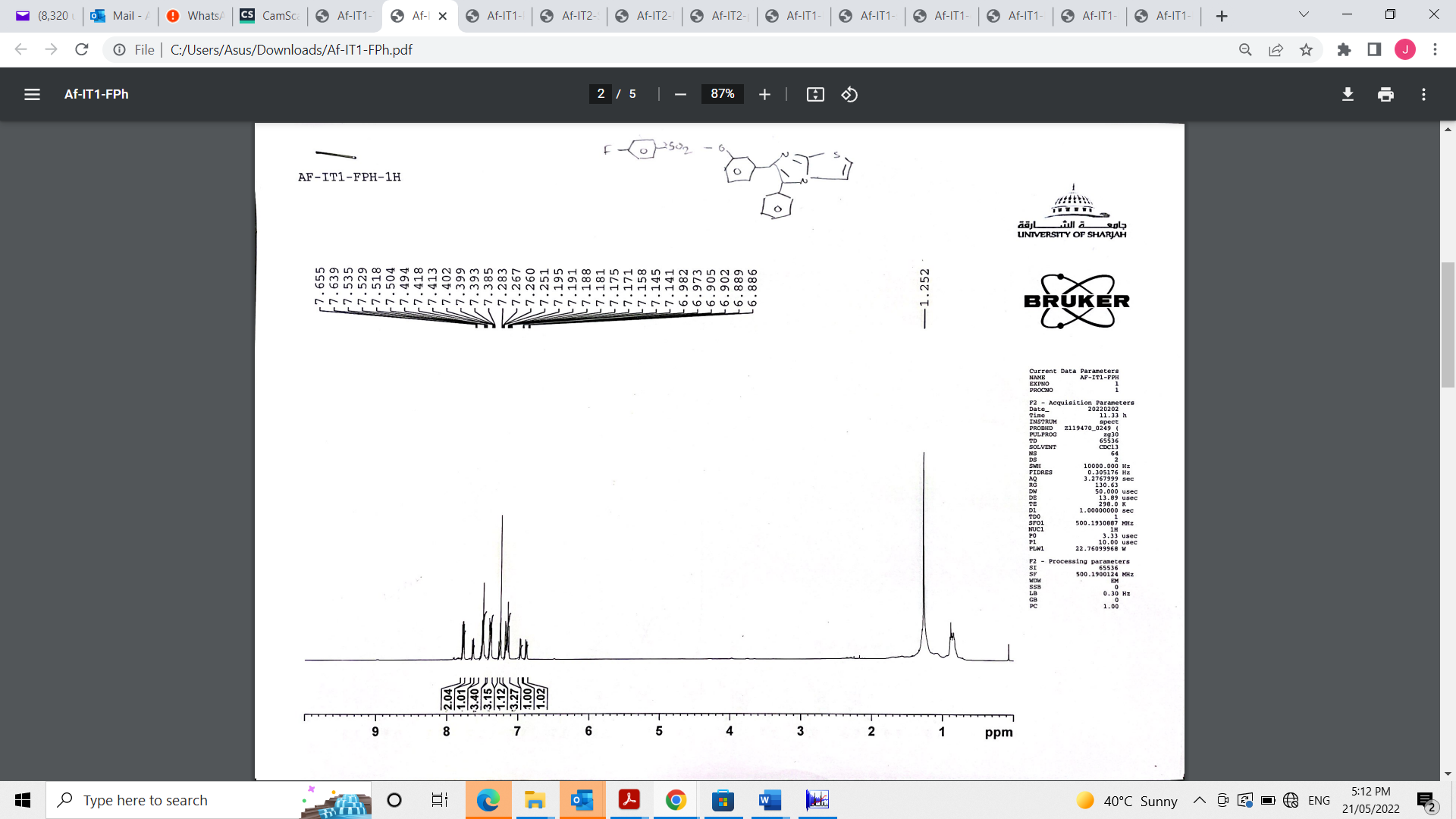


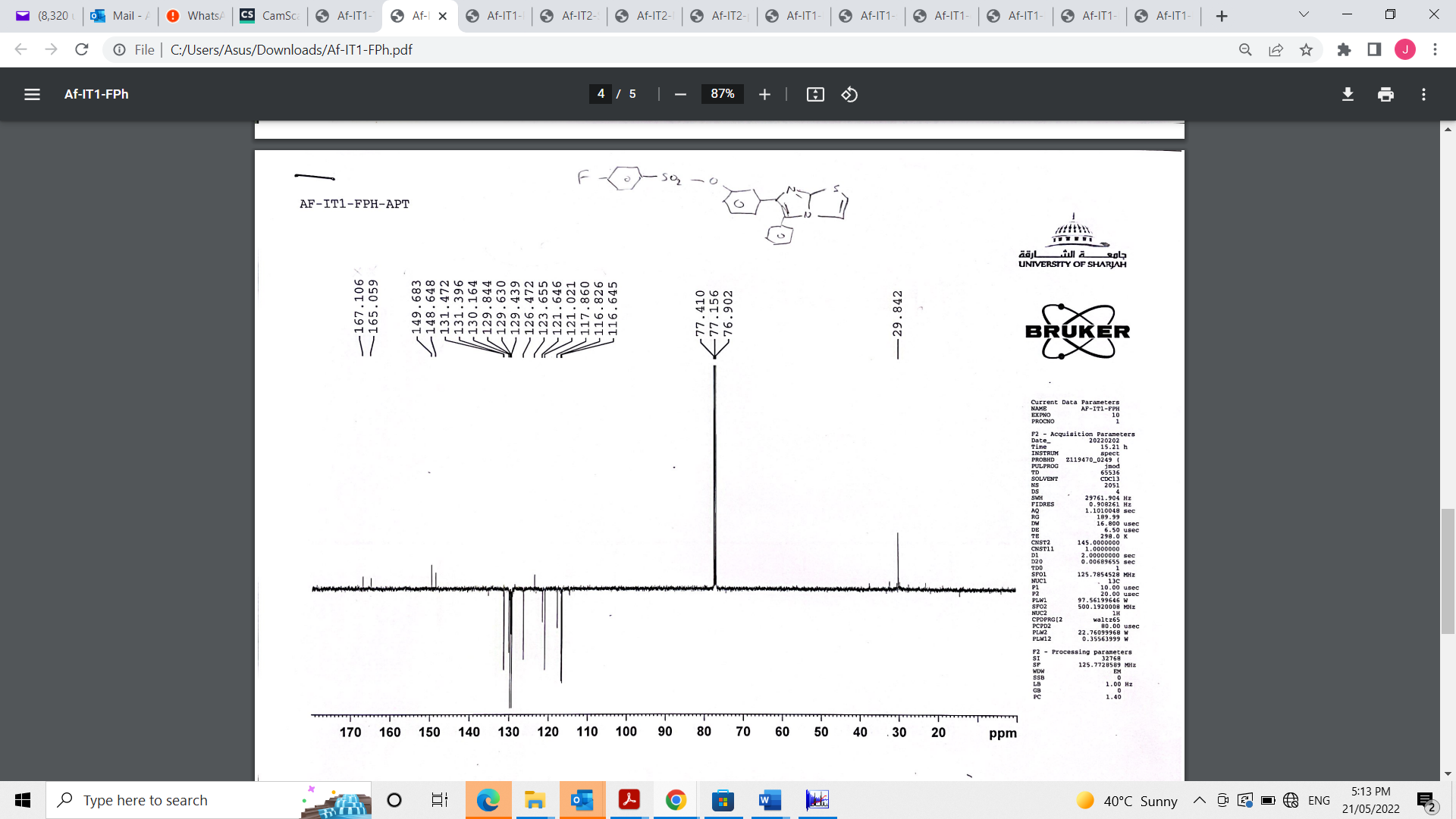


**Figure S12.** ^1^H NMR and ^13^C NMR charts of compound **1f**.

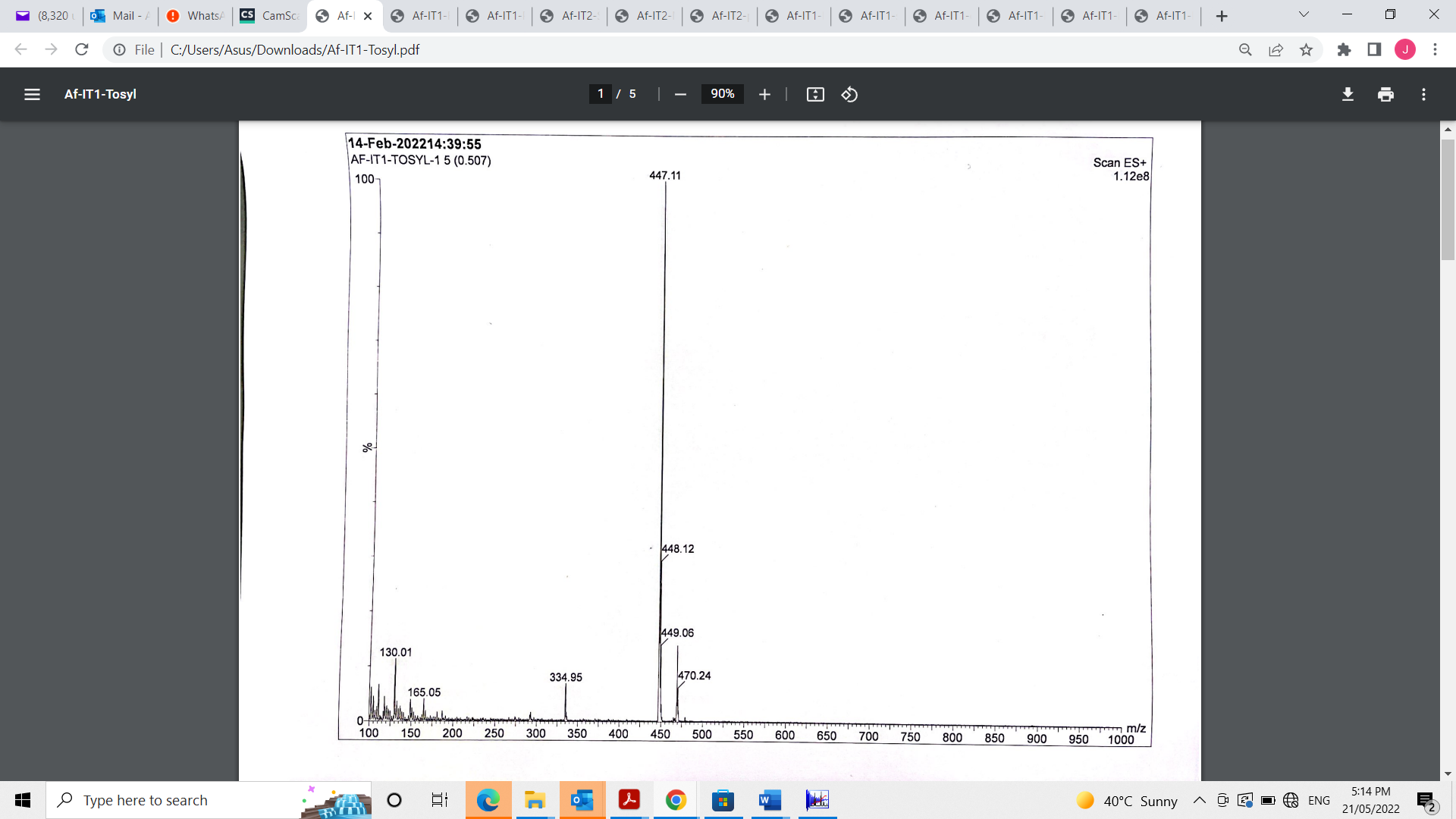


**Figure S13.** LC-MS chart of compound **1g**.


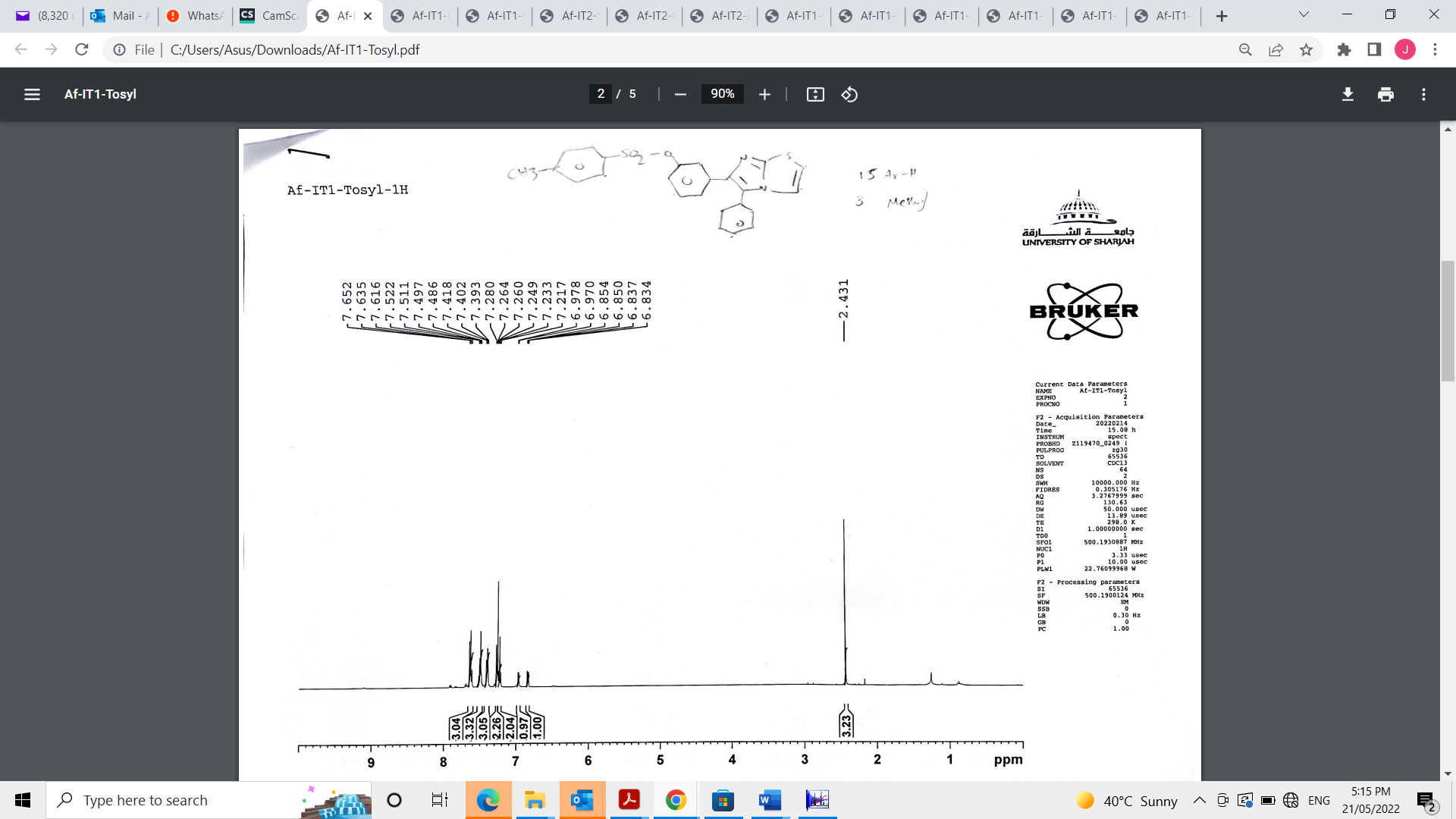


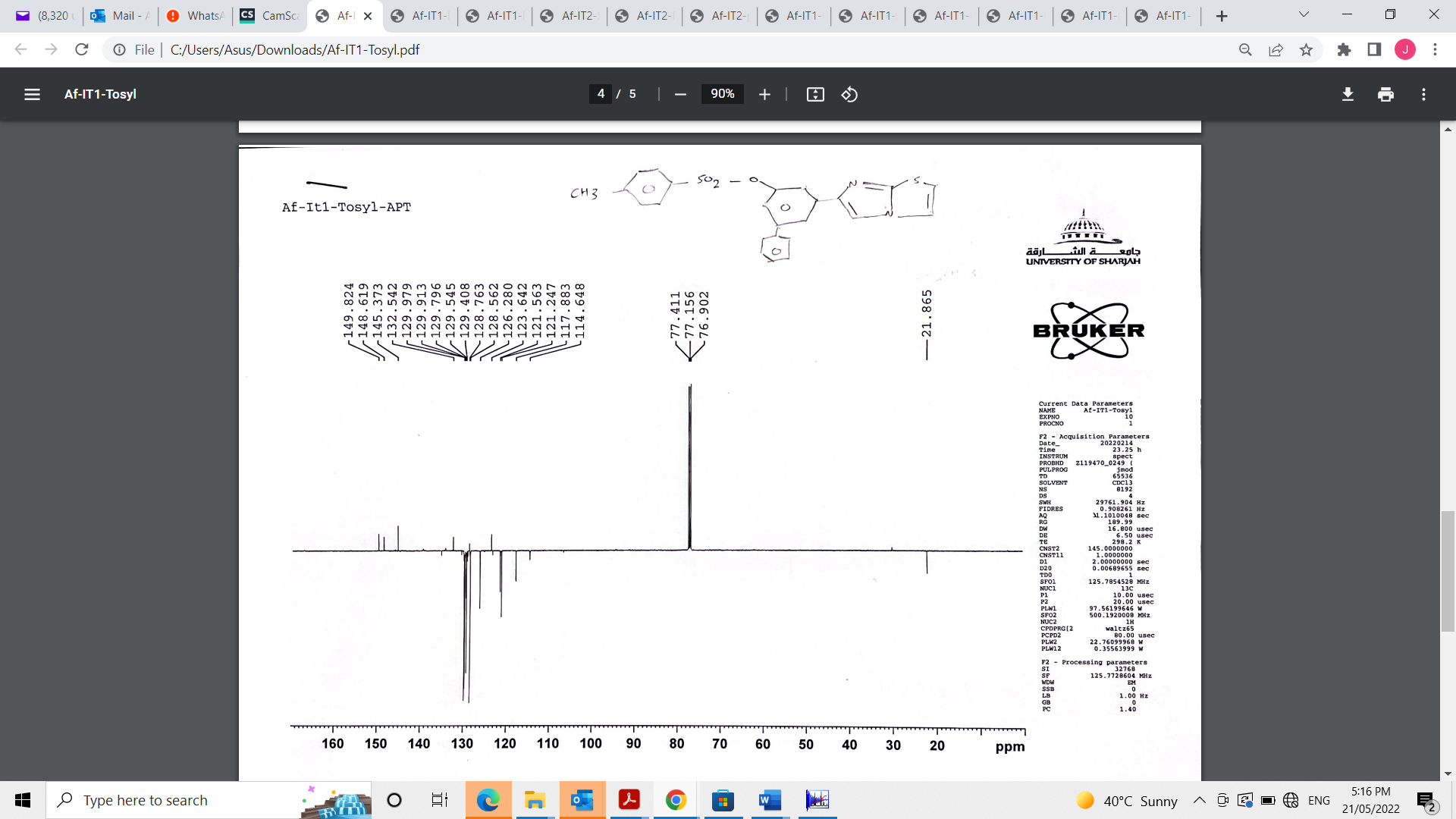


**Figure S14.** ^1^H NMR and ^13^C NMR charts of compound **1g**.

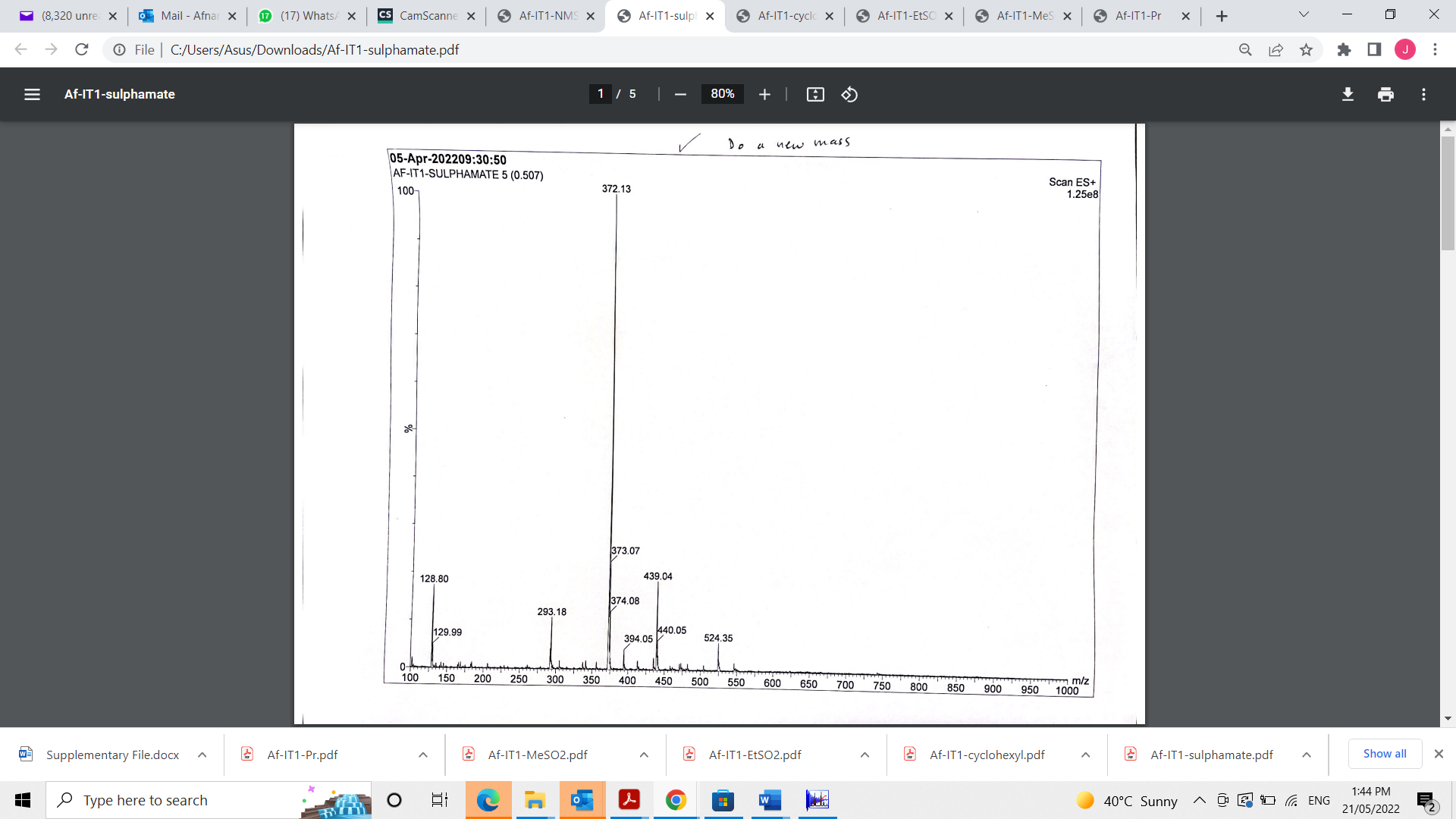


**Figure S15.** LC-MS chart of compound **1h**.


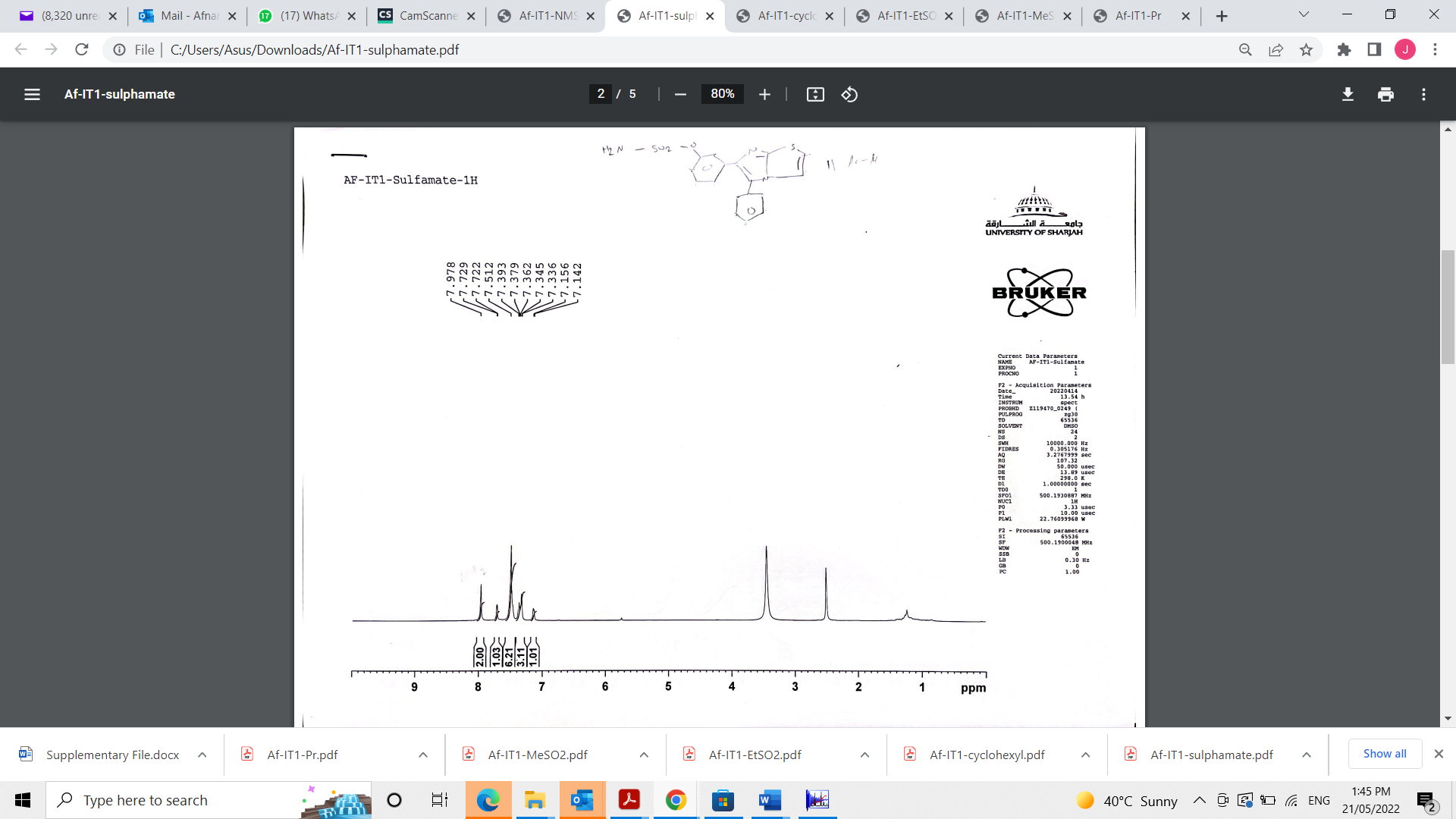


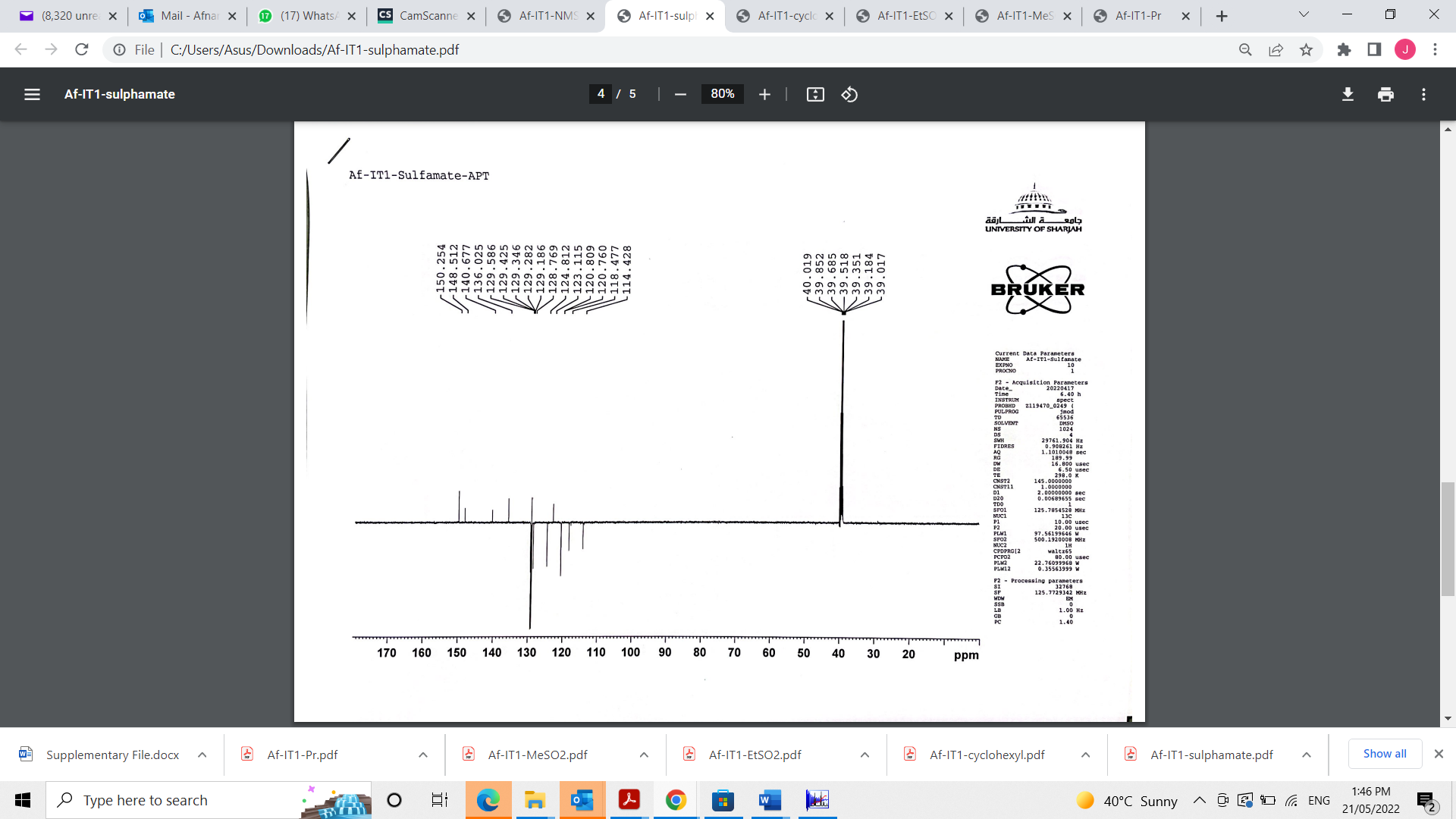


**Figure S16.** ^1^H NMR and ^13^C NMR charts of compound **1h**.

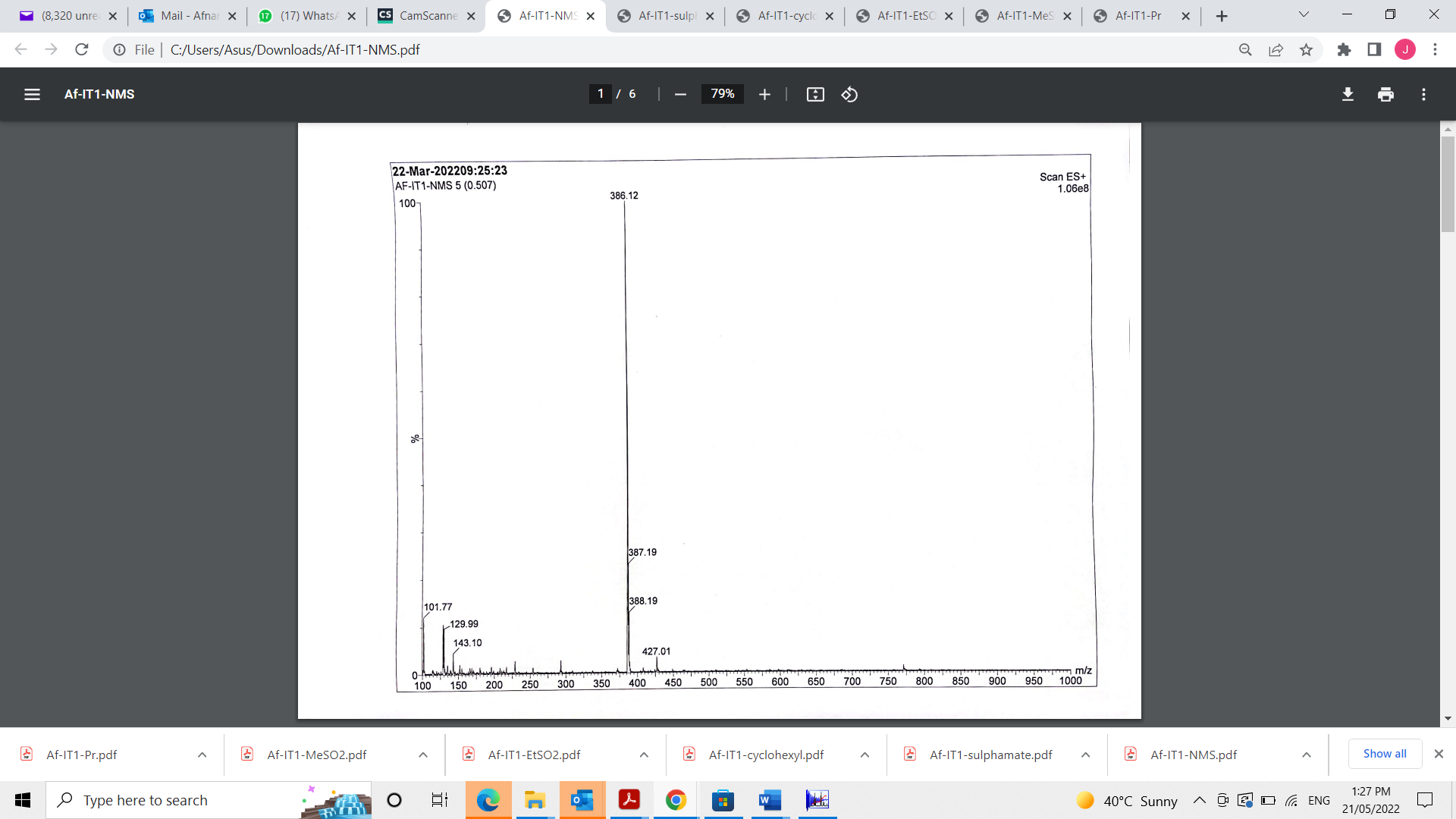


**Figure S17.** LC-MS chart of compound **1i**.


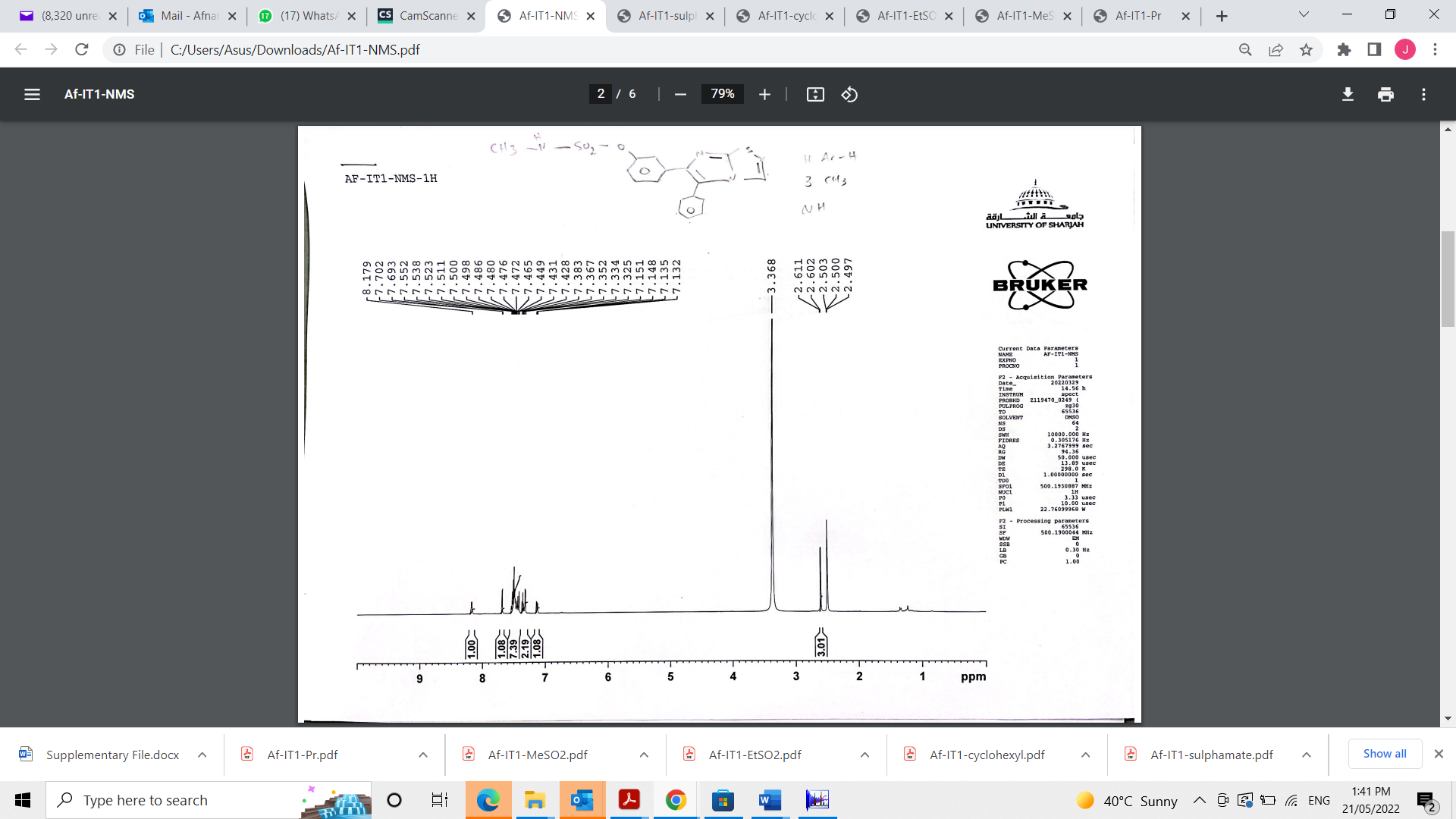


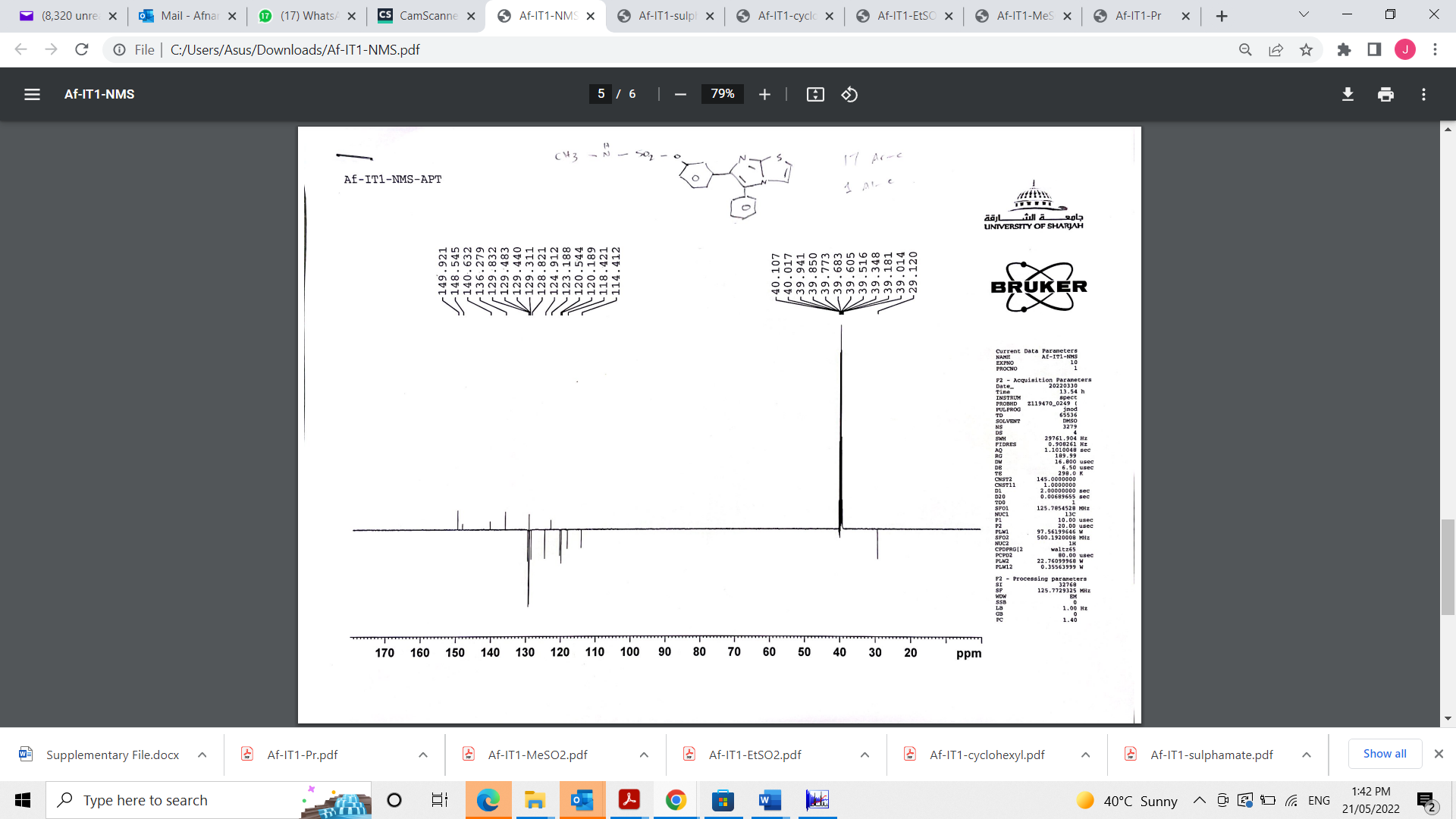


**Figure S18.** ^1^H NMR and ^13^C NMR charts of compound **1i**.

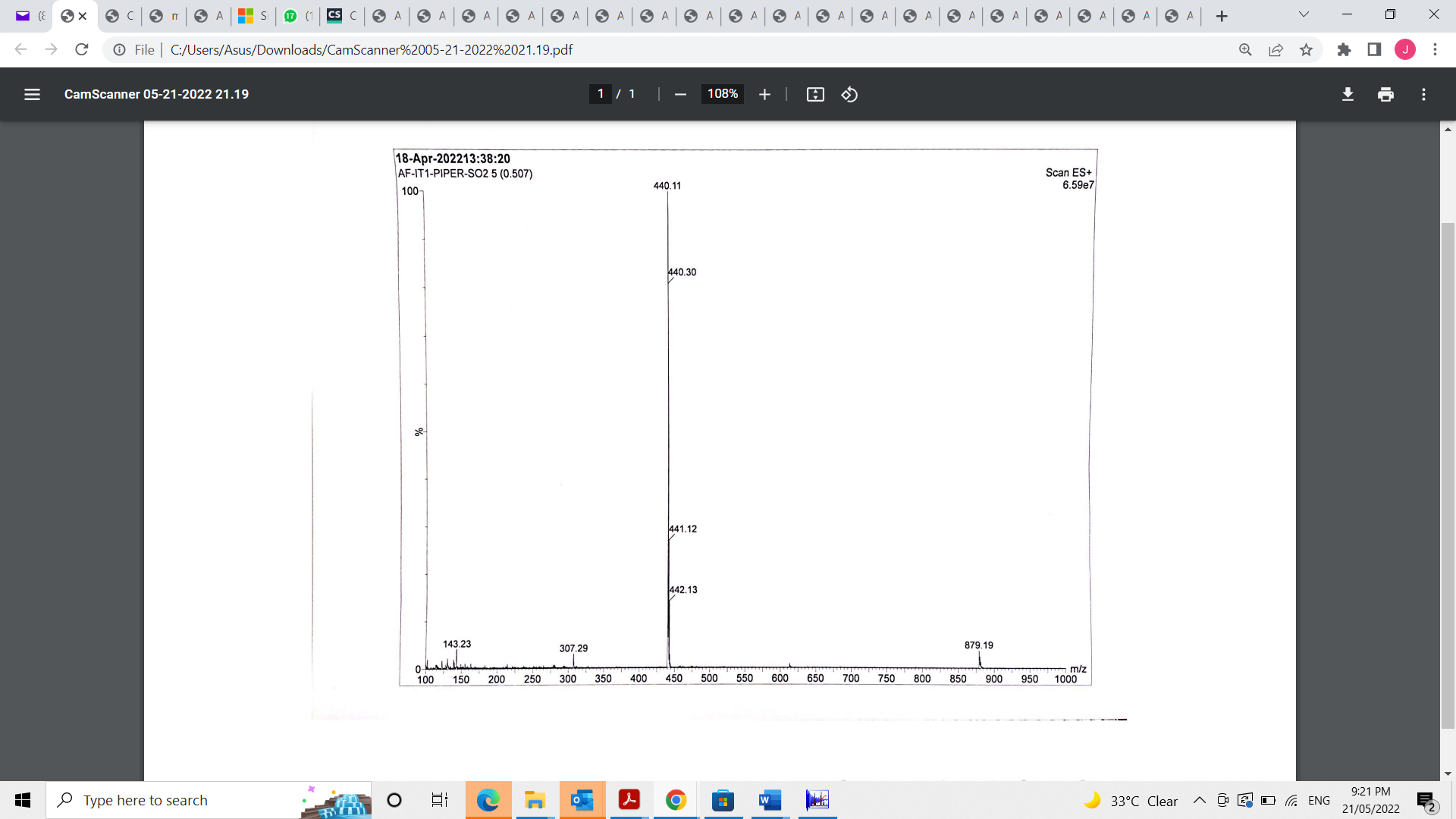


**Figure S19.** LC-MS chart of compound **1j**.


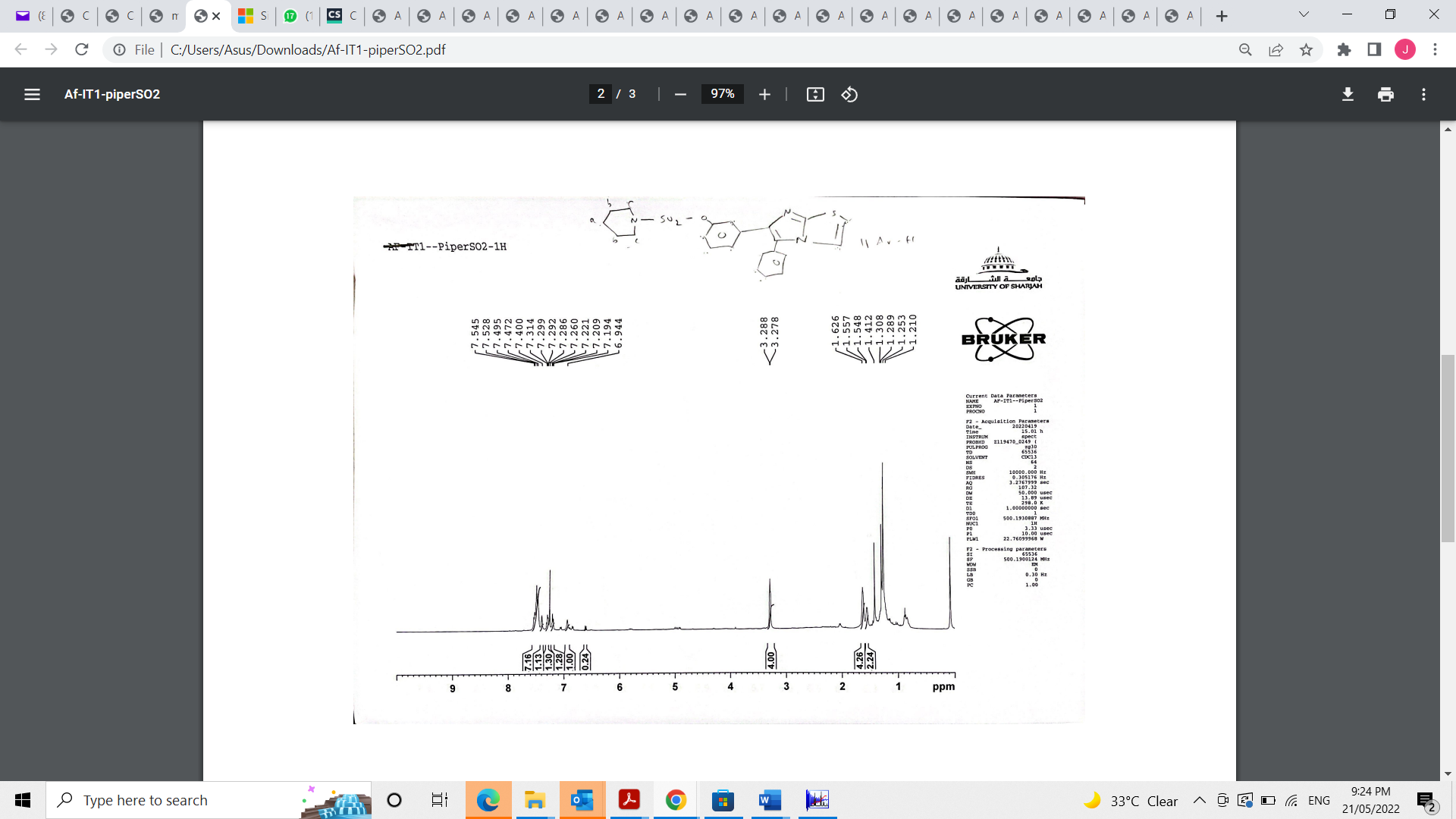


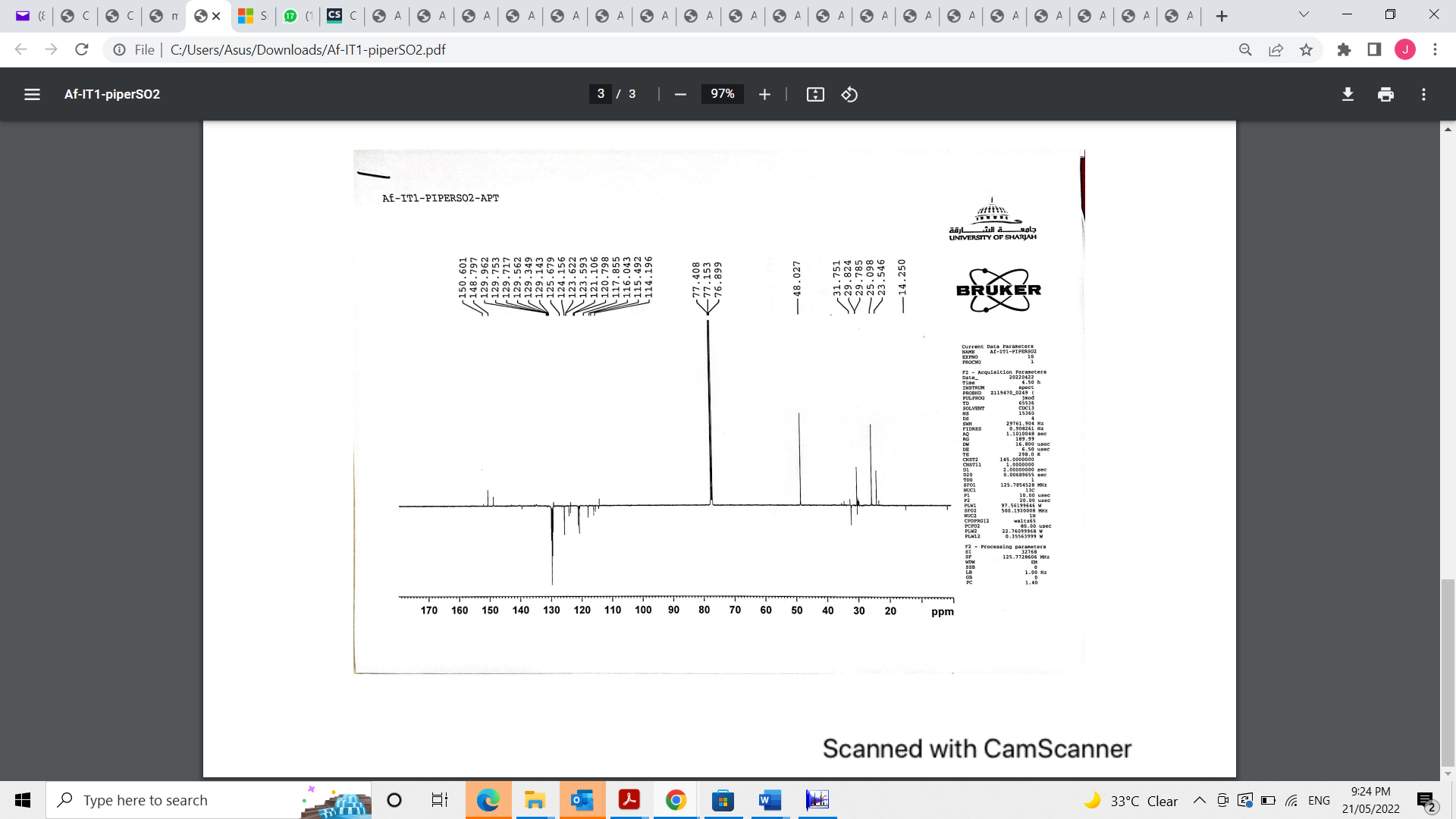


**Figure S20.** ^1^H NMR and ^13^C NMR charts of compound **1j**.

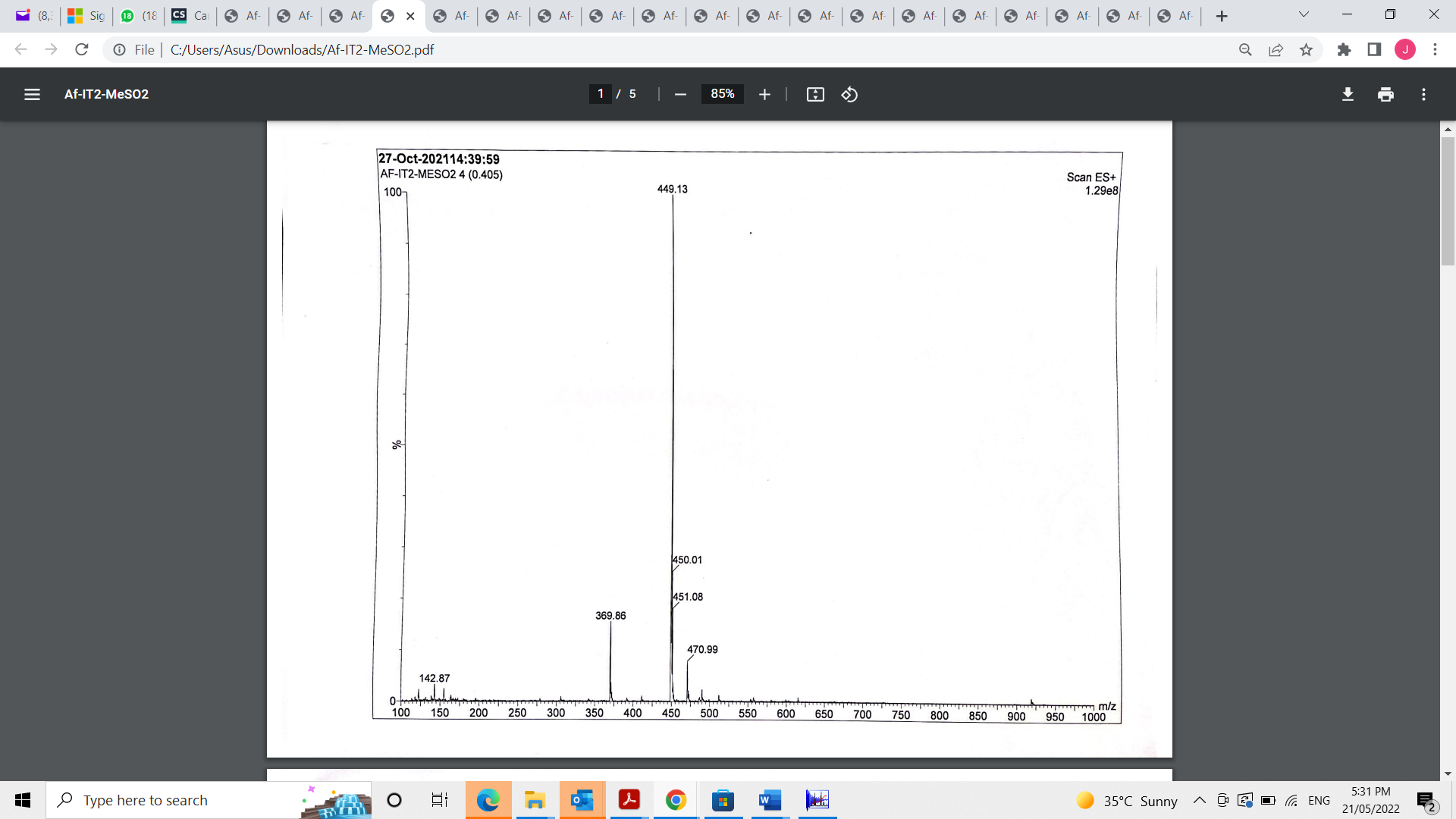


**Figure S21.** LC-MS chart of compound **2a**.


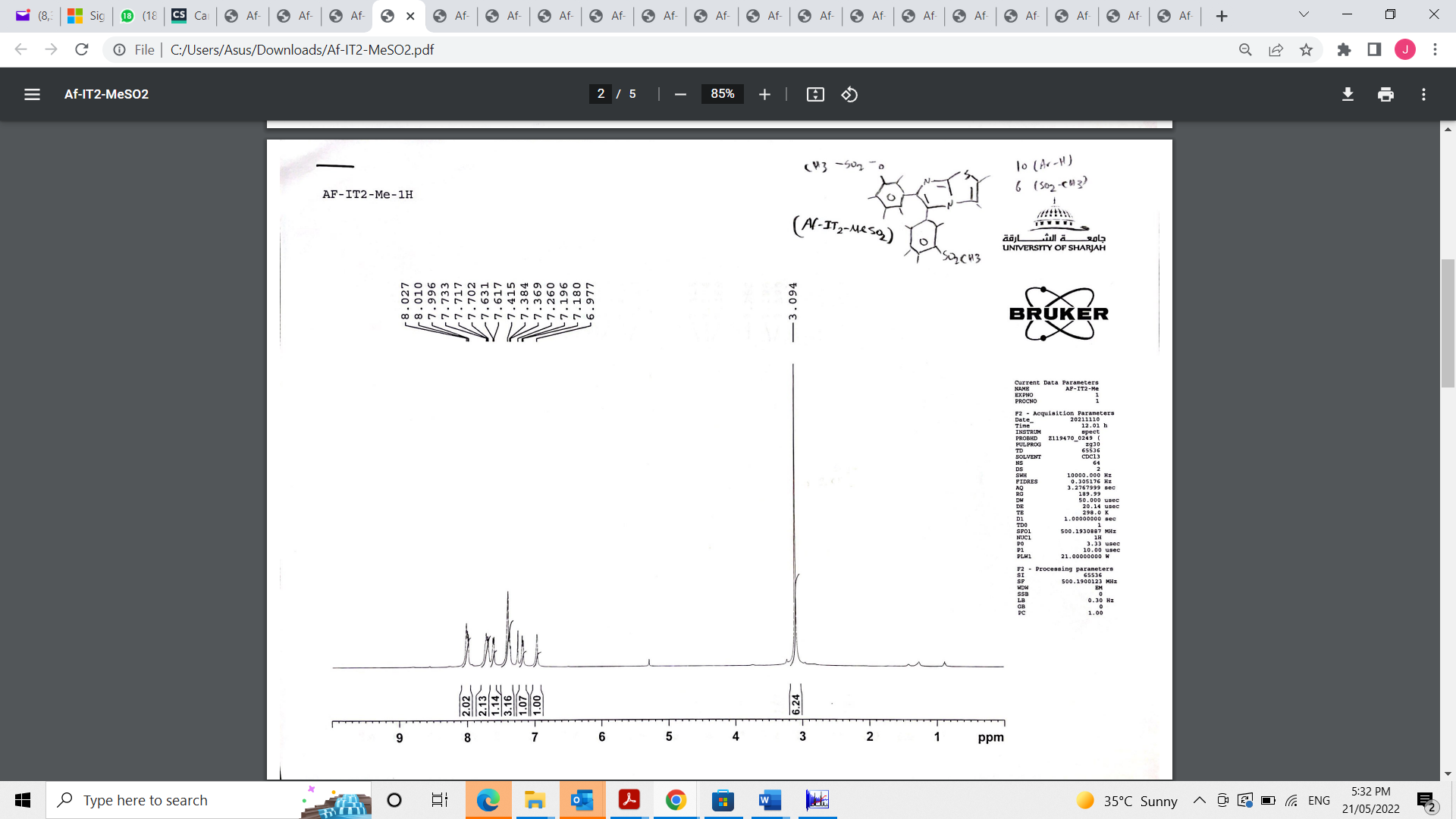


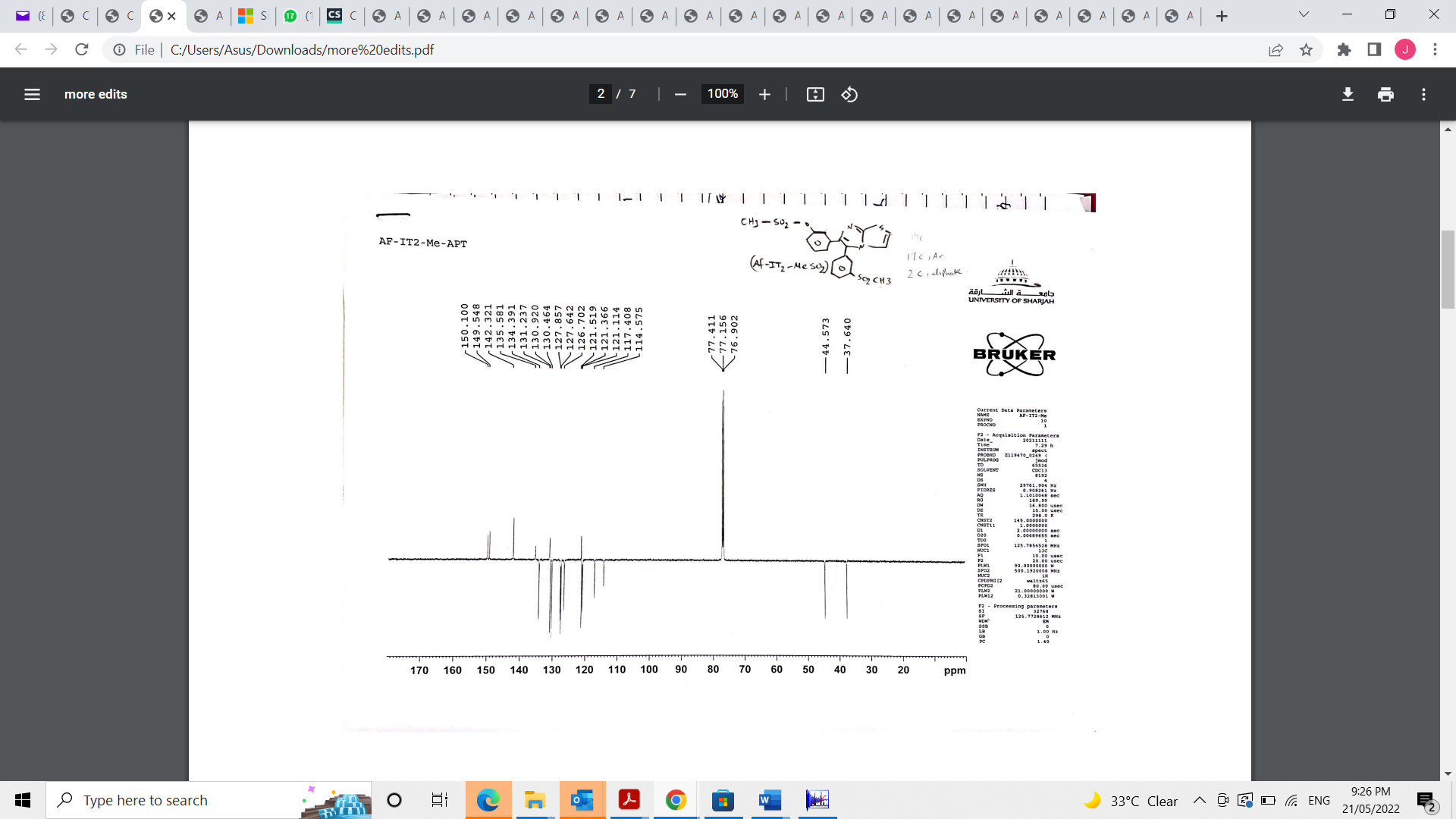


**Figure S22.** ^1^H NMR and ^13^C NMR charts of compound **2a**.

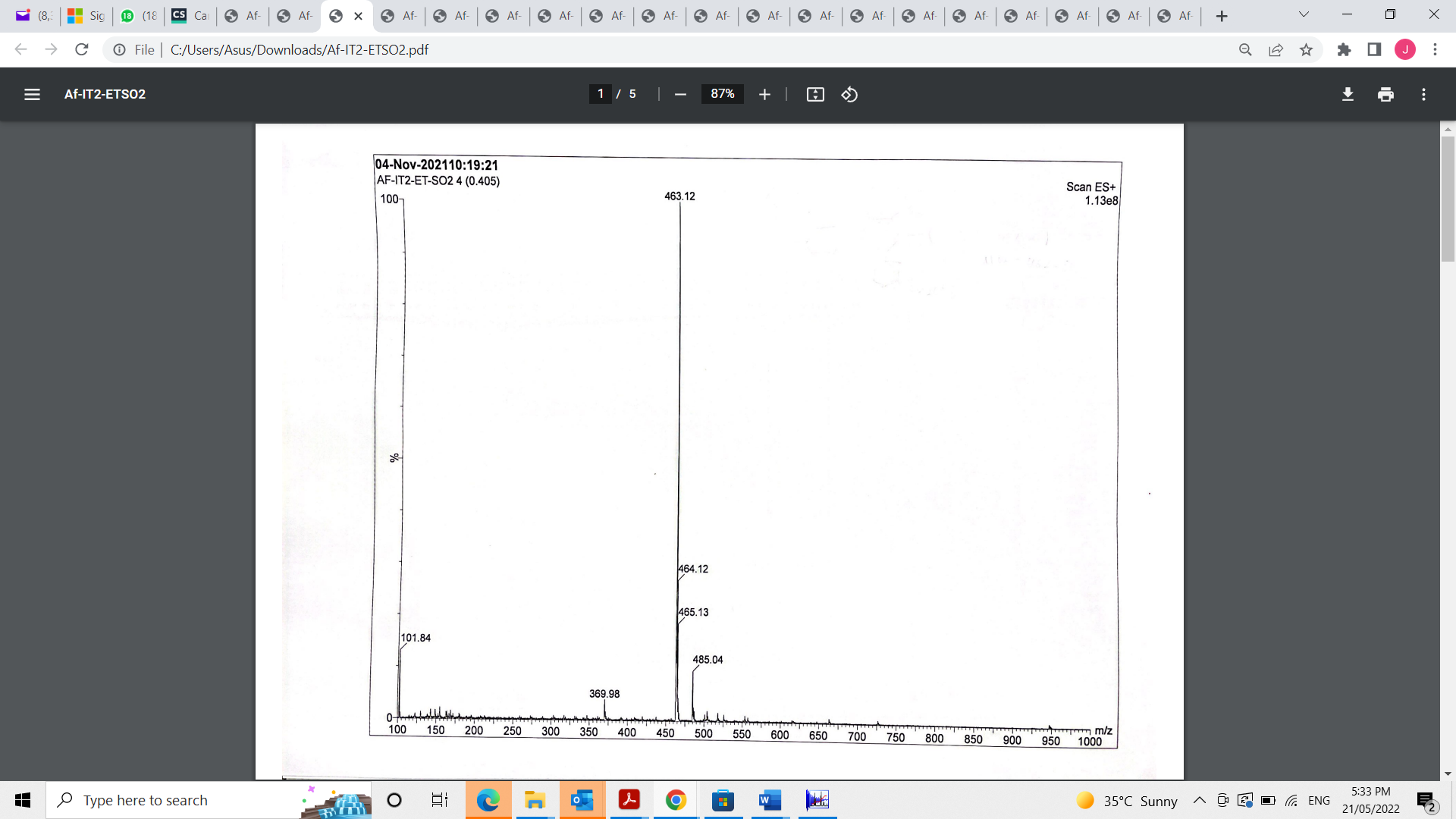


**Figure S23.** LC-MS chart of compound **2b**.


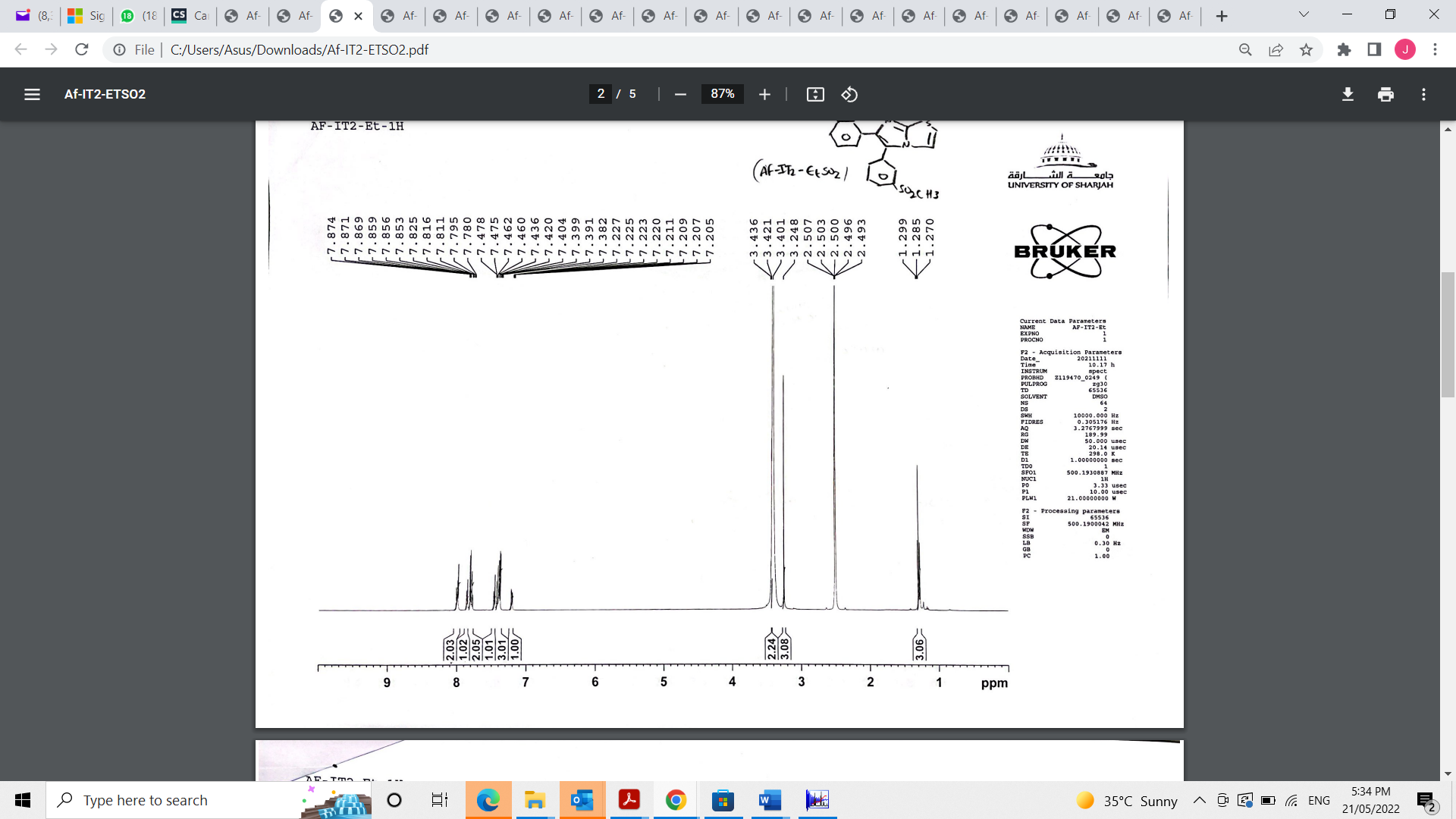


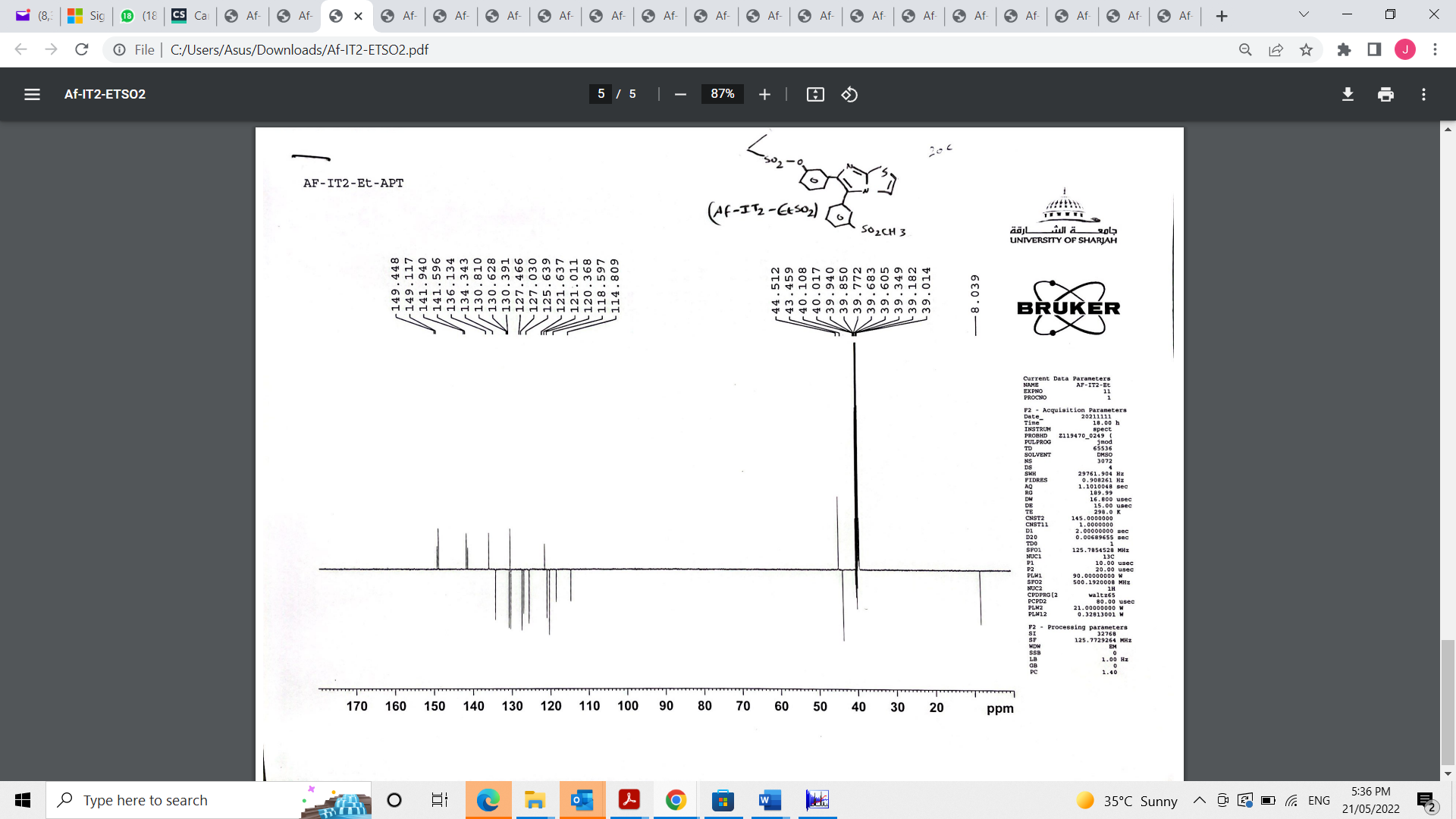


**Figure S24.** ^1^H NMR and ^13^C NMR charts of compound **2b**.

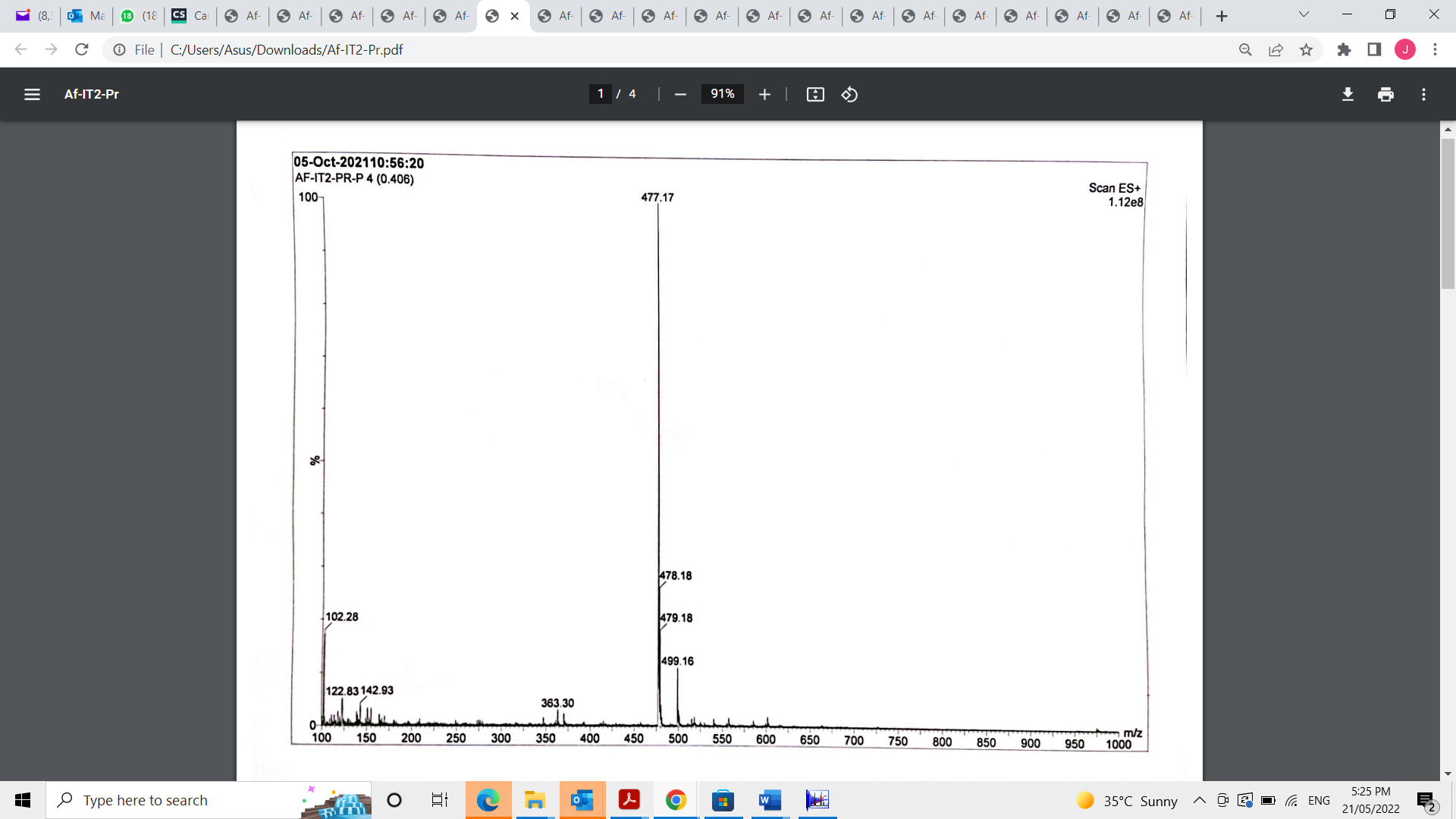


**Figure S25.** LC-MS chart of compound **2c**.


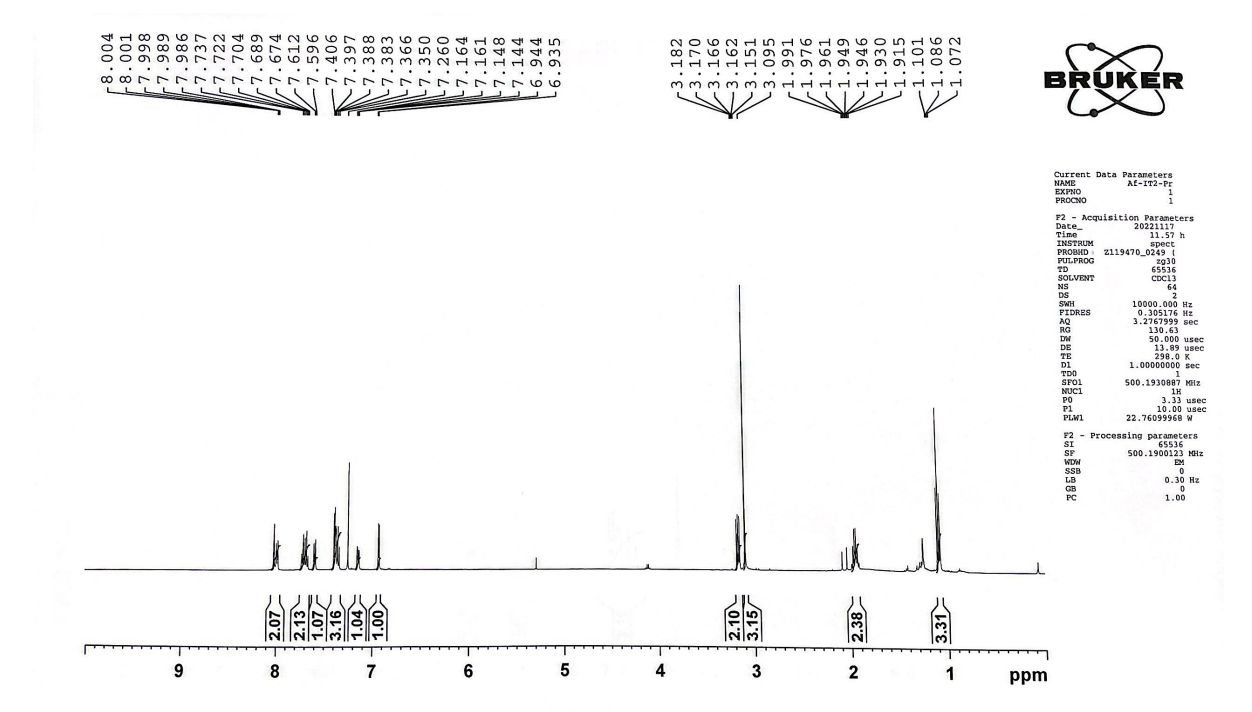


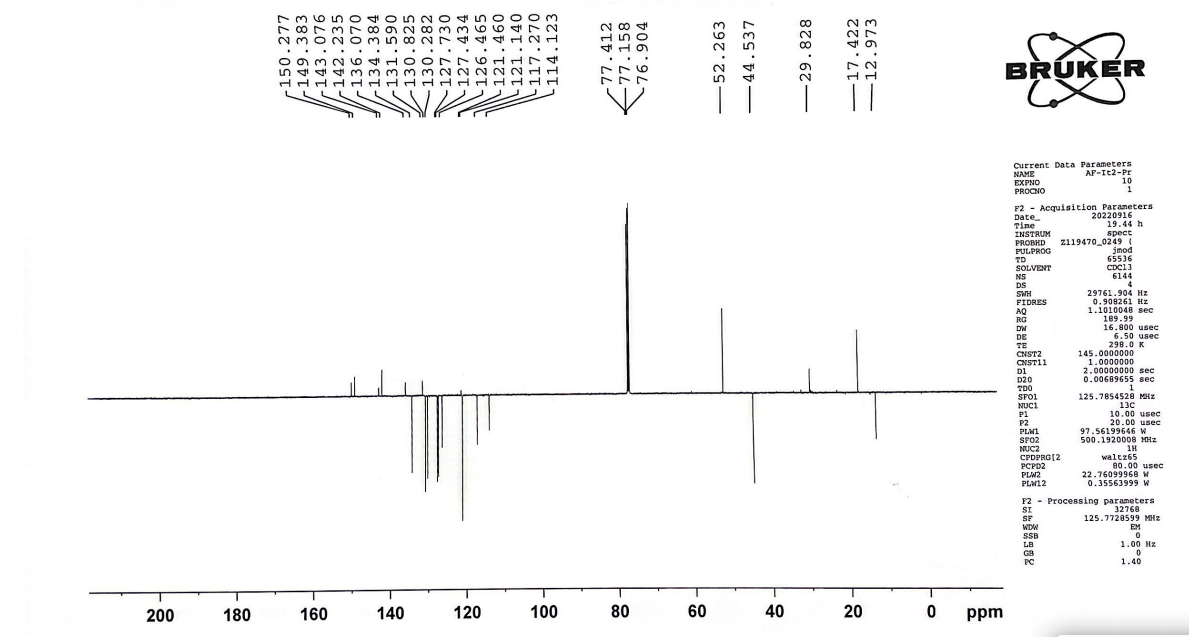


**Figure S26.** ^1^H NMR and ^13^C NMR charts of compound **2c**.

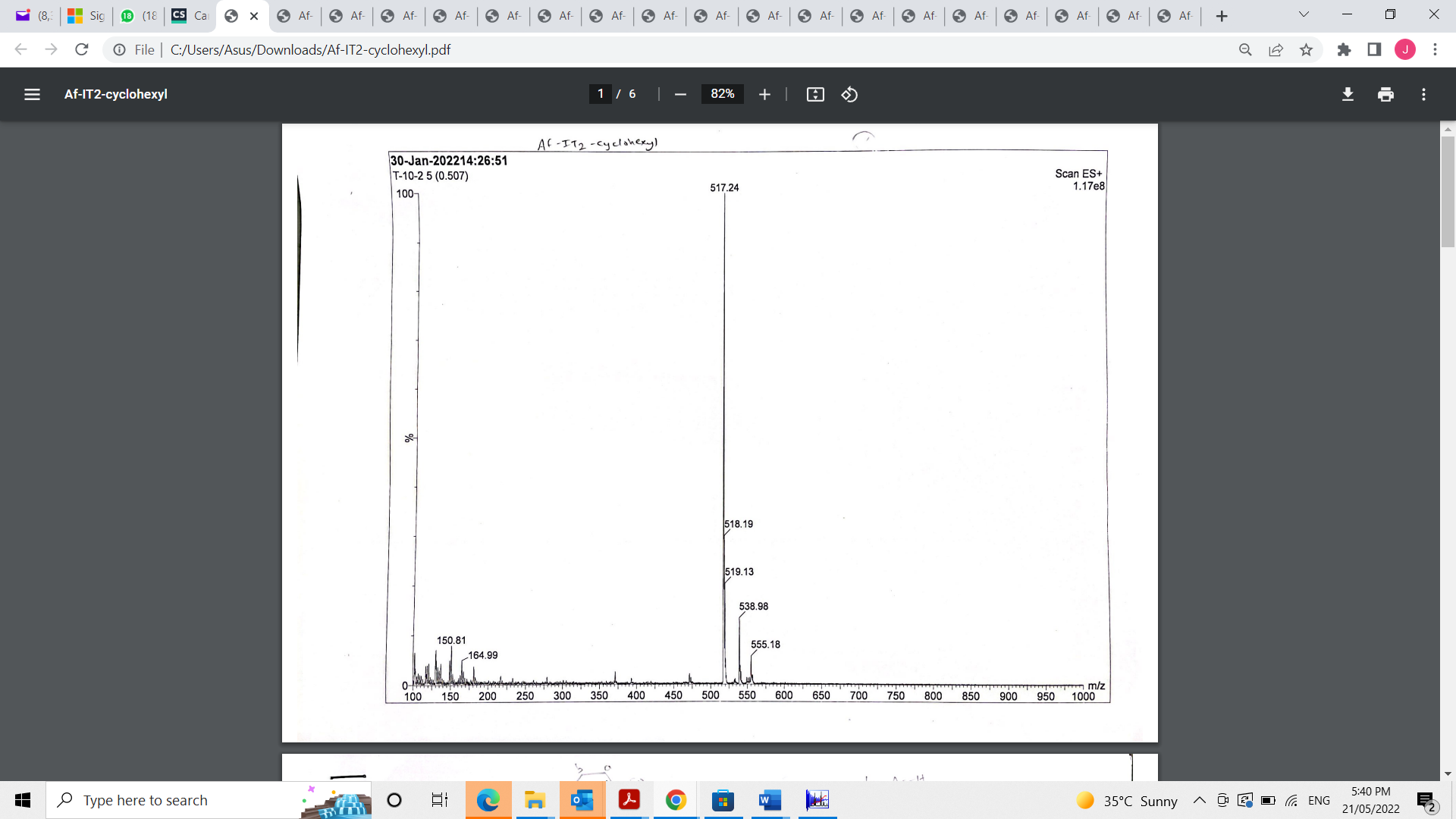


**Figure S27.** LC-MS chart of compound **2d**.


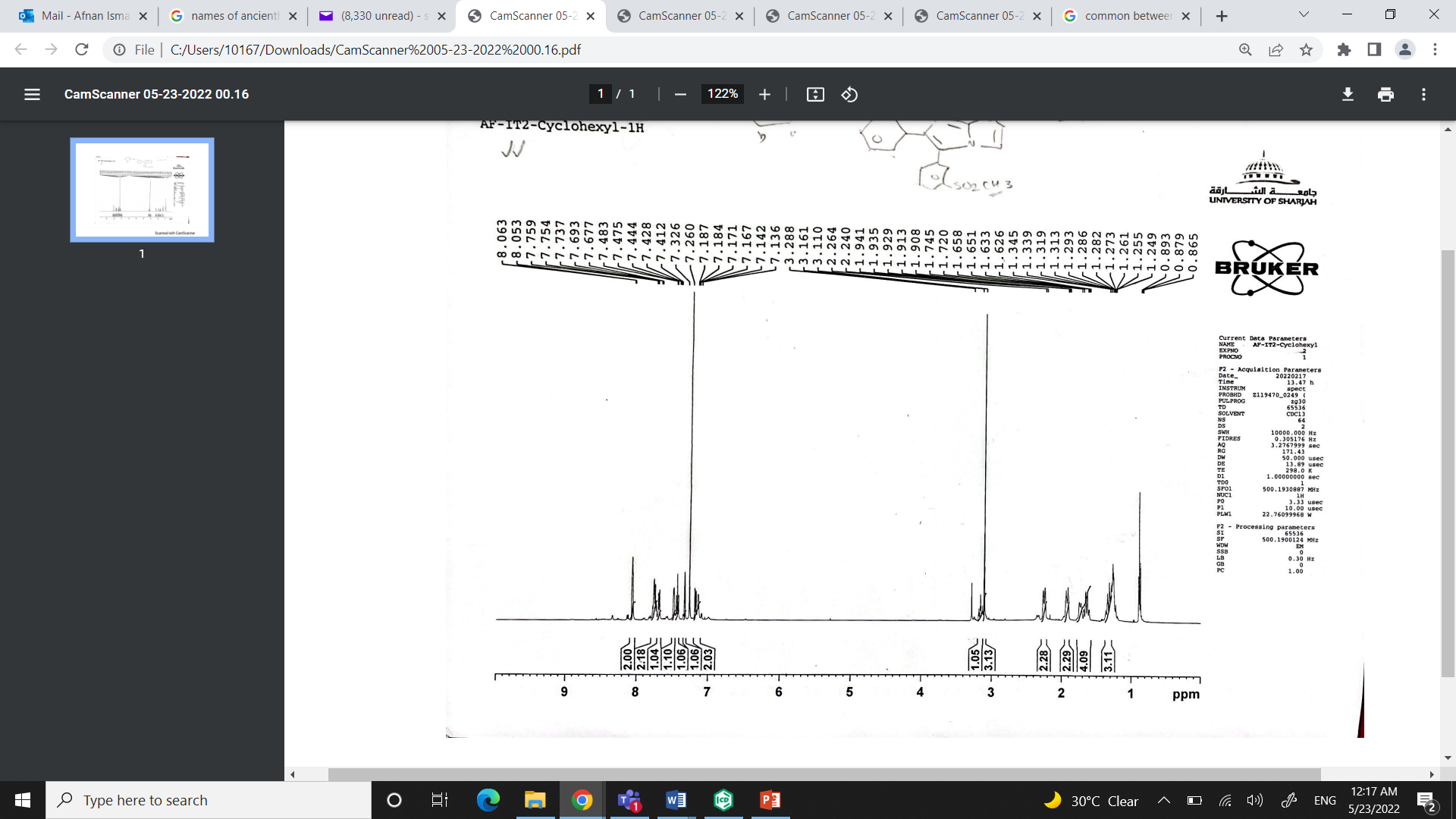


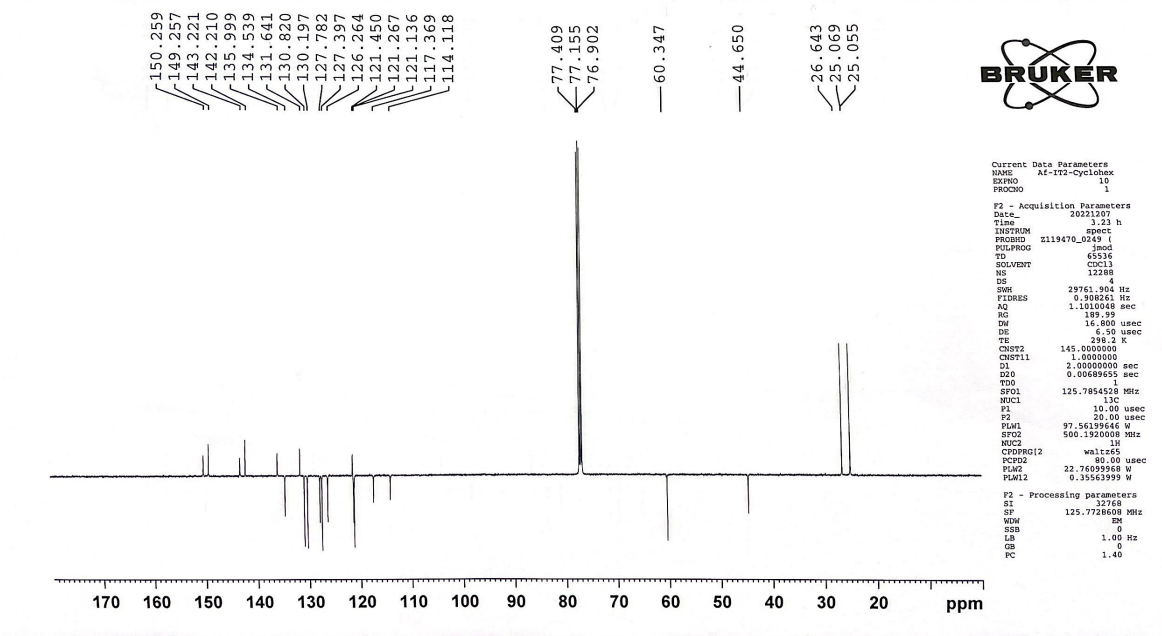


**Figure S28.** ^1^H NMR and ^13^C NMR charts of compound **2d**.

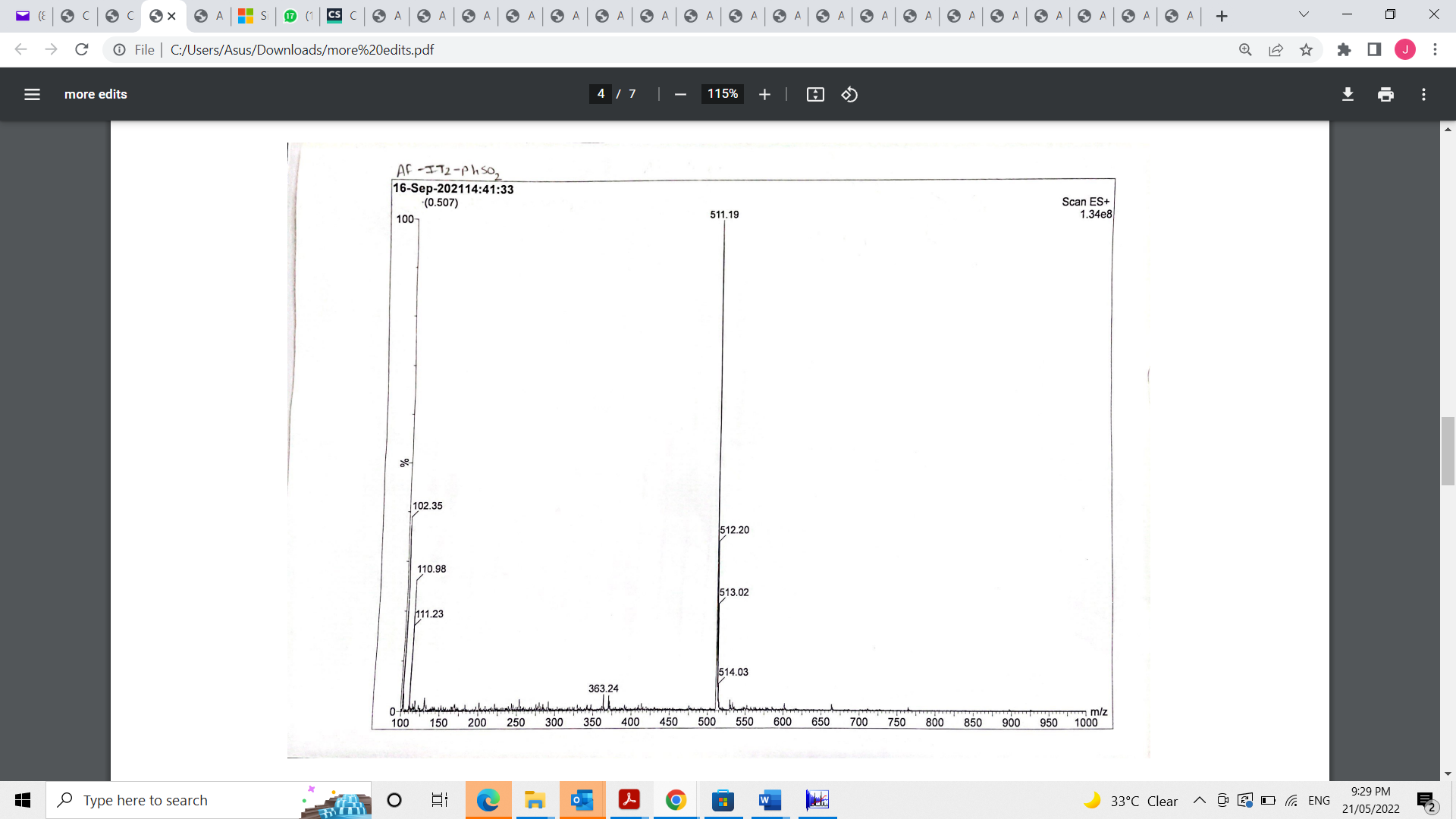


**Figure S29.** LC-MS chart of compound **2e**.


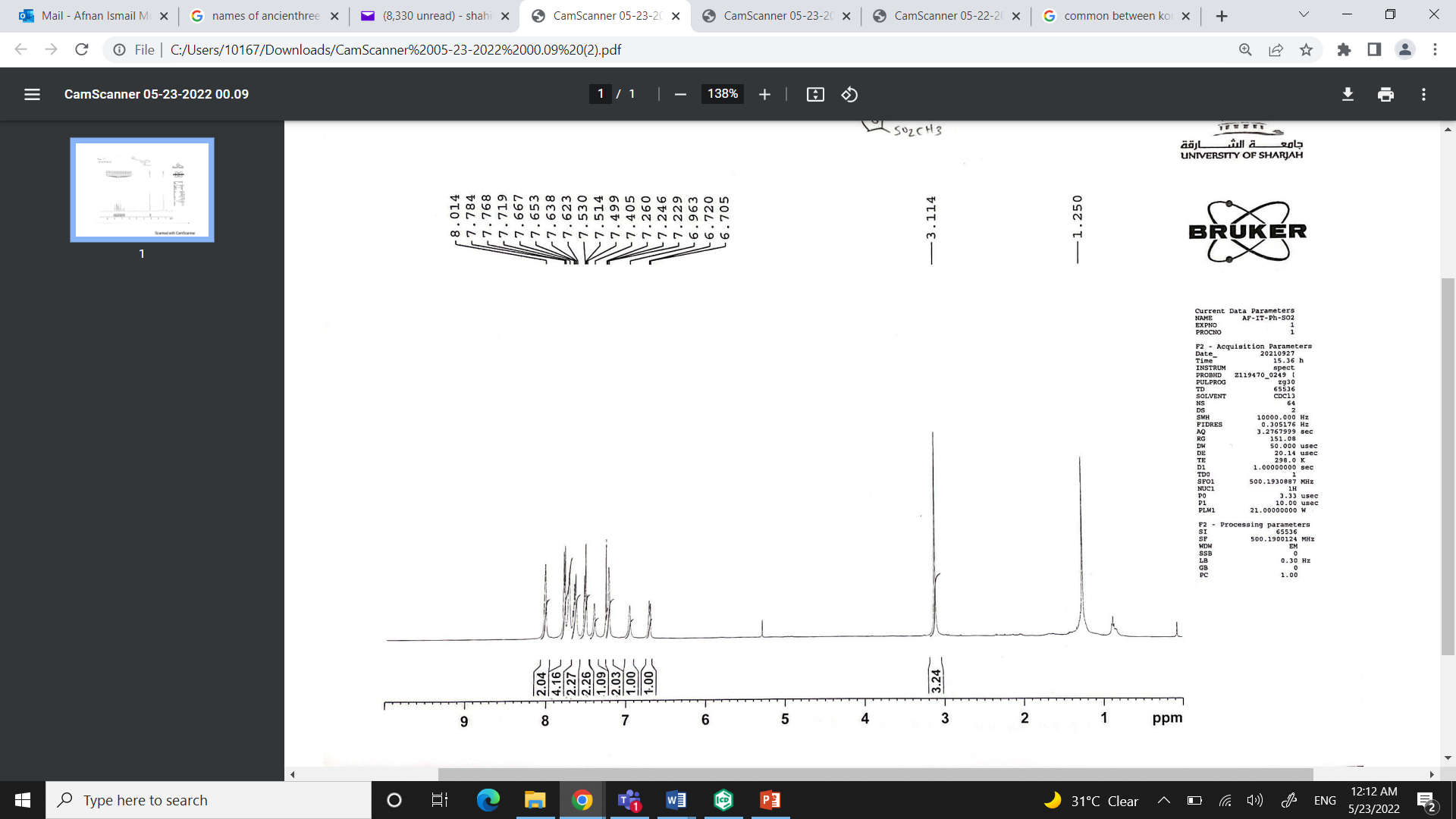


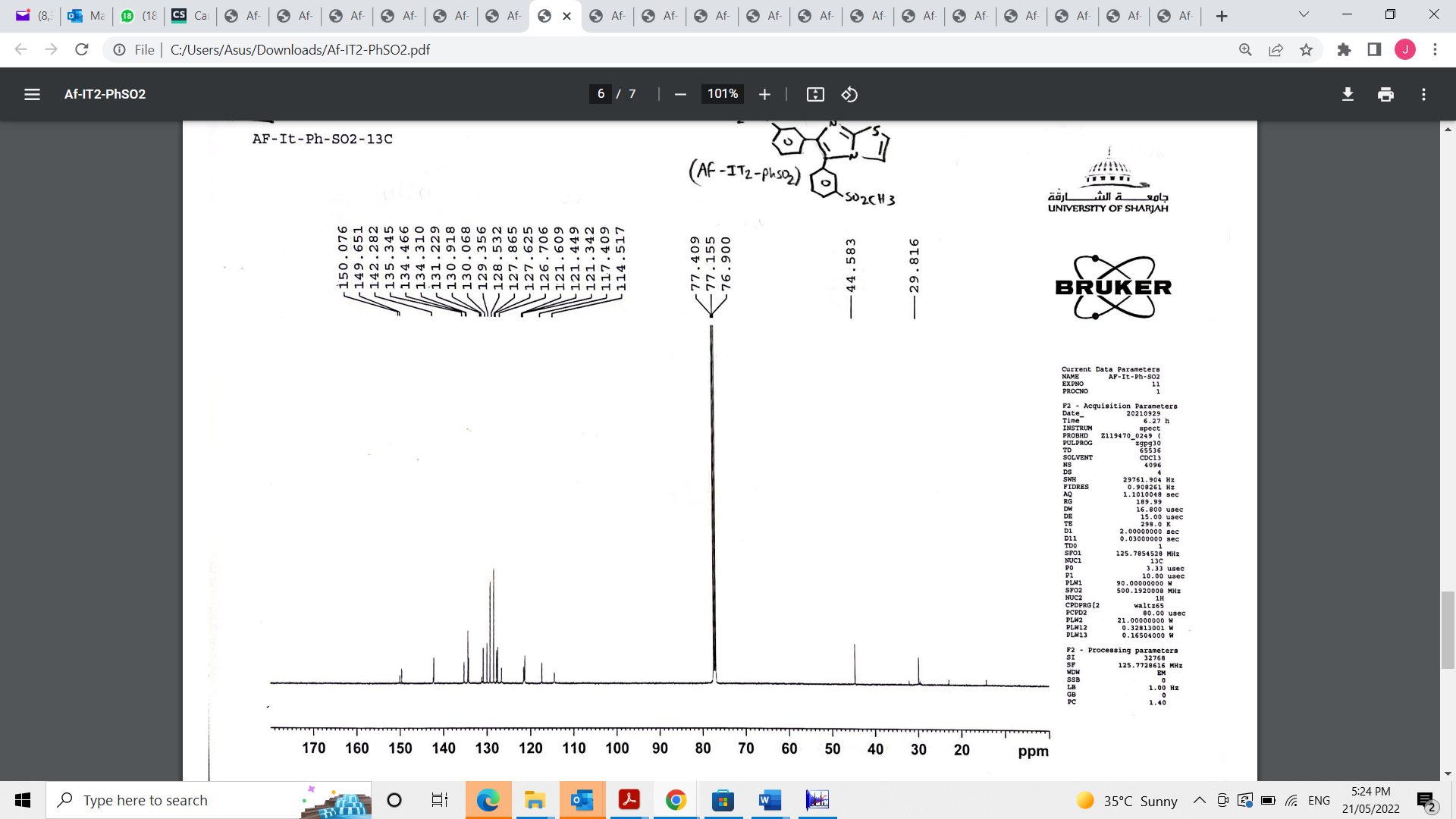


**Figure S30.** ^1^H NMR and ^13^C NMR charts of compound **2e**.

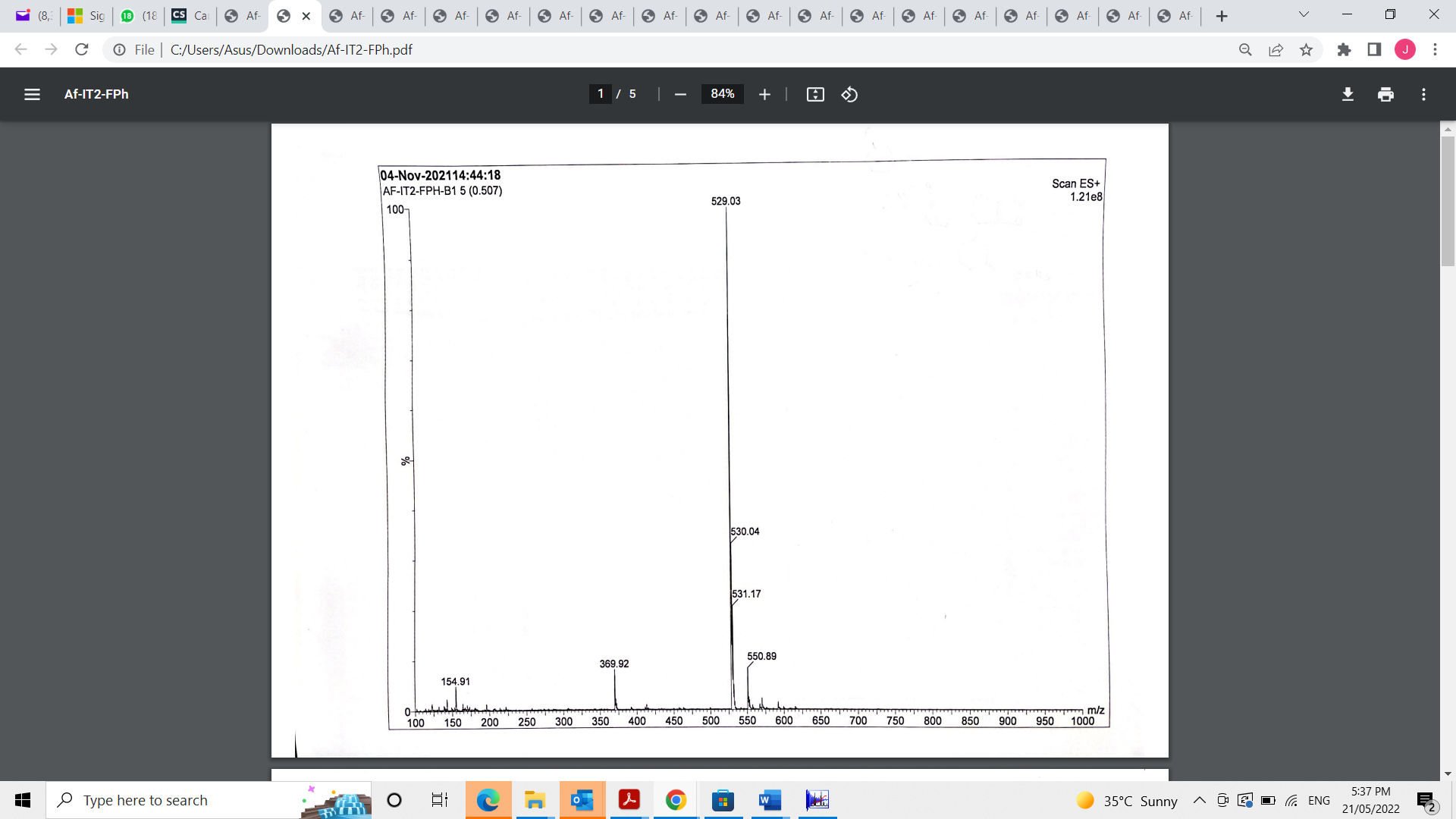


**Figure S31.** LC-MS chart of compound **2f**.


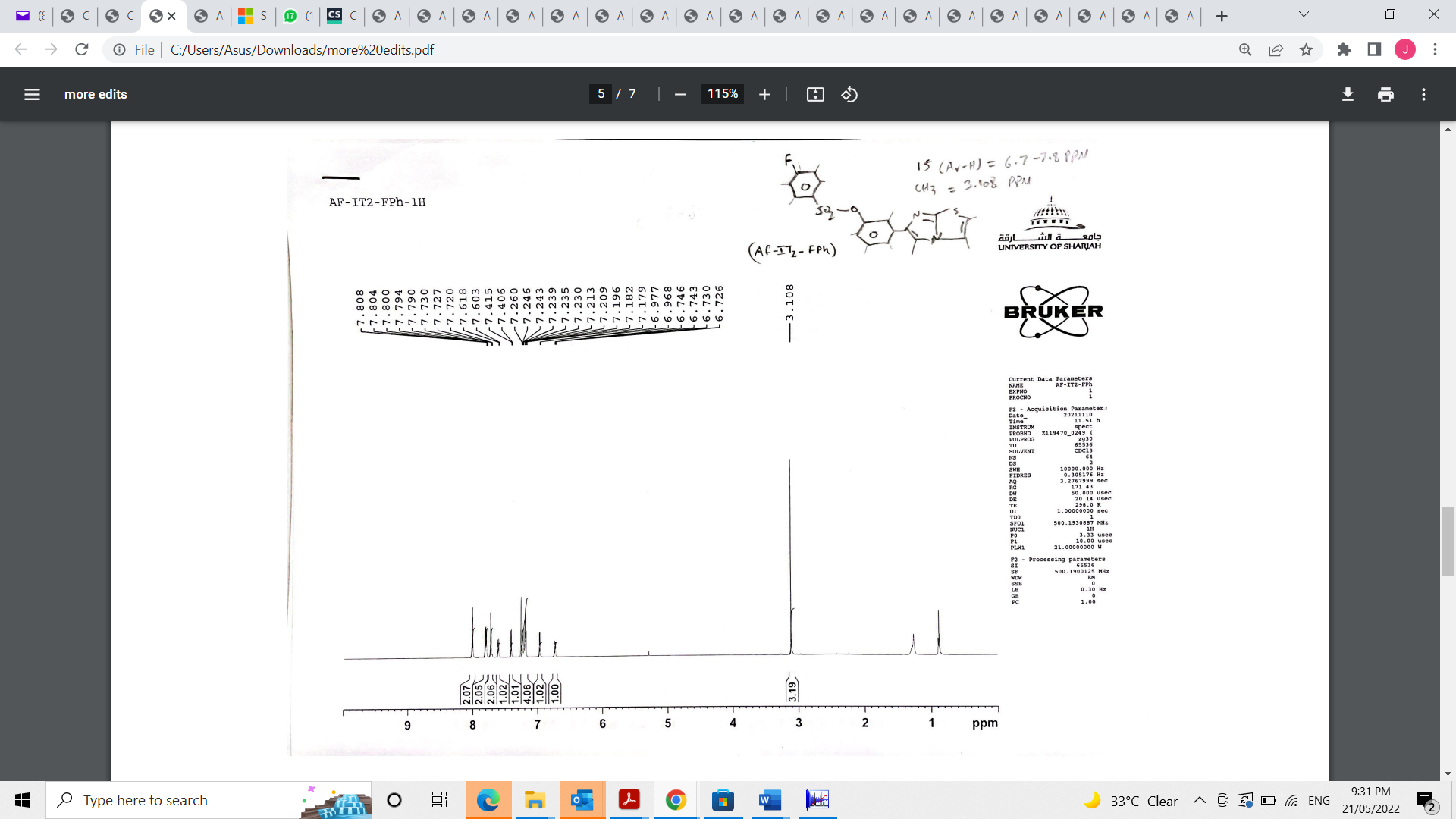


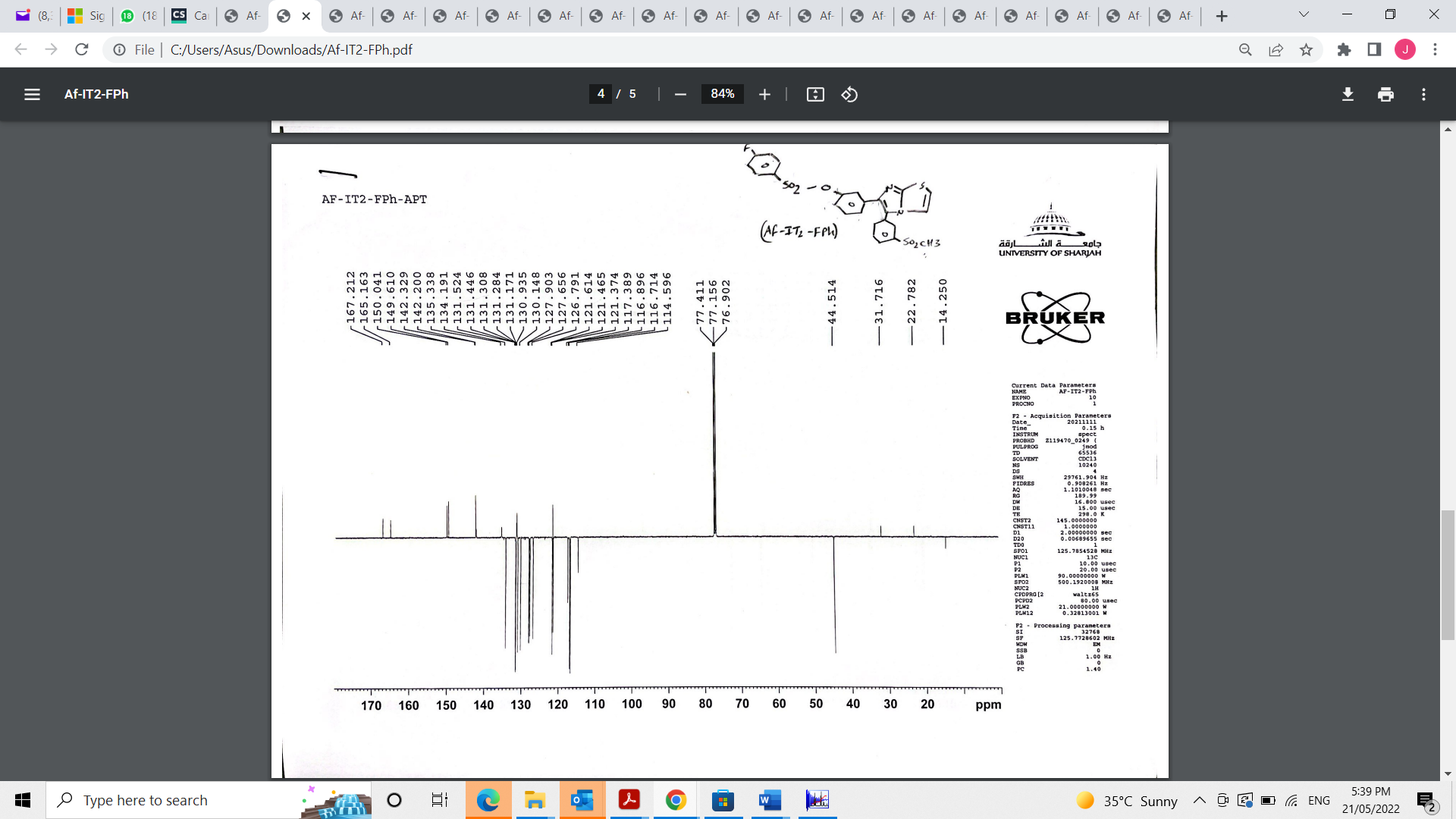


**Figure S32.** ^1^H NMR and ^13^C NMR charts of compound **2f**.

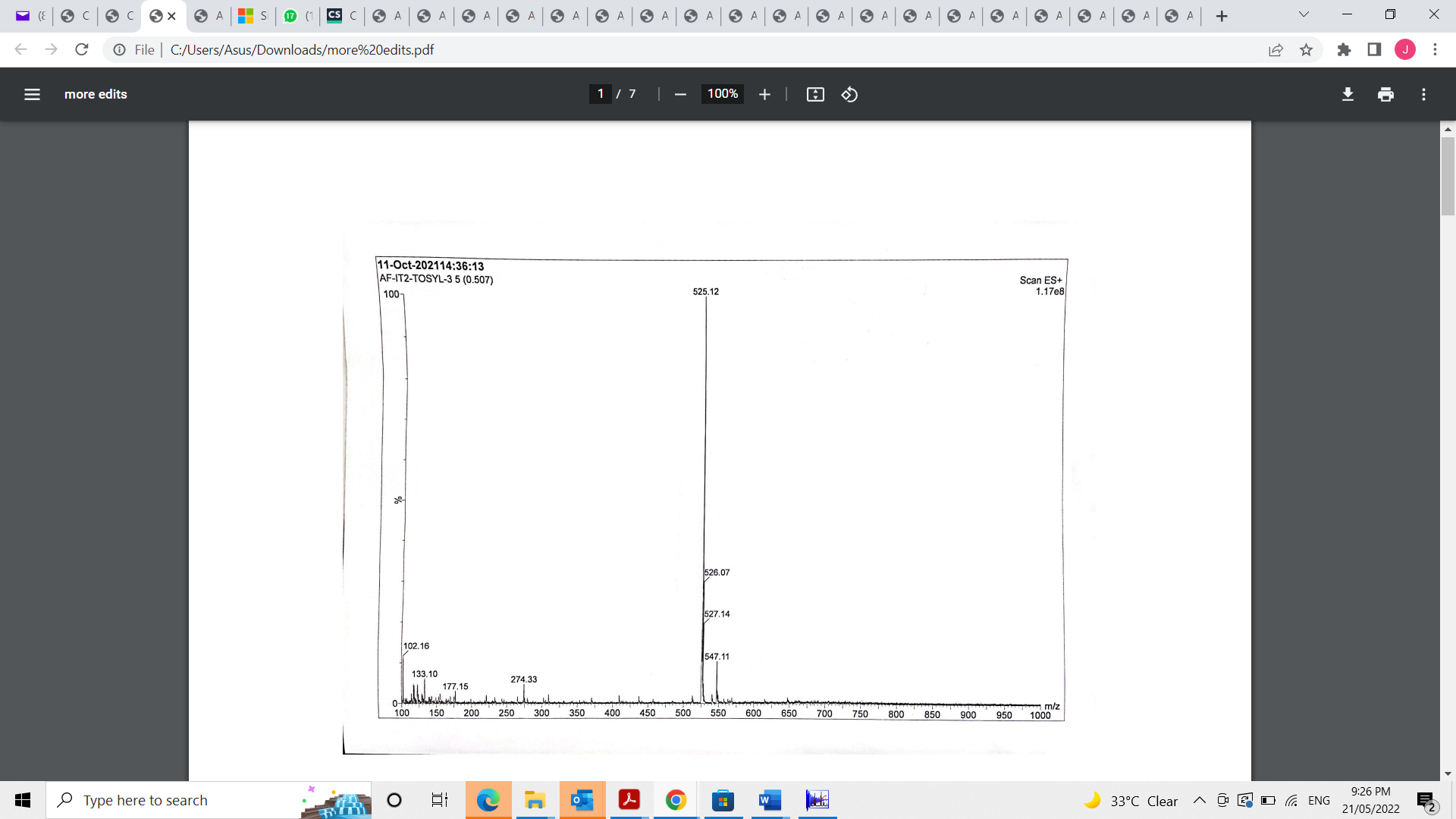


**Figure S33.** LC-MS chart of compound **2g**.


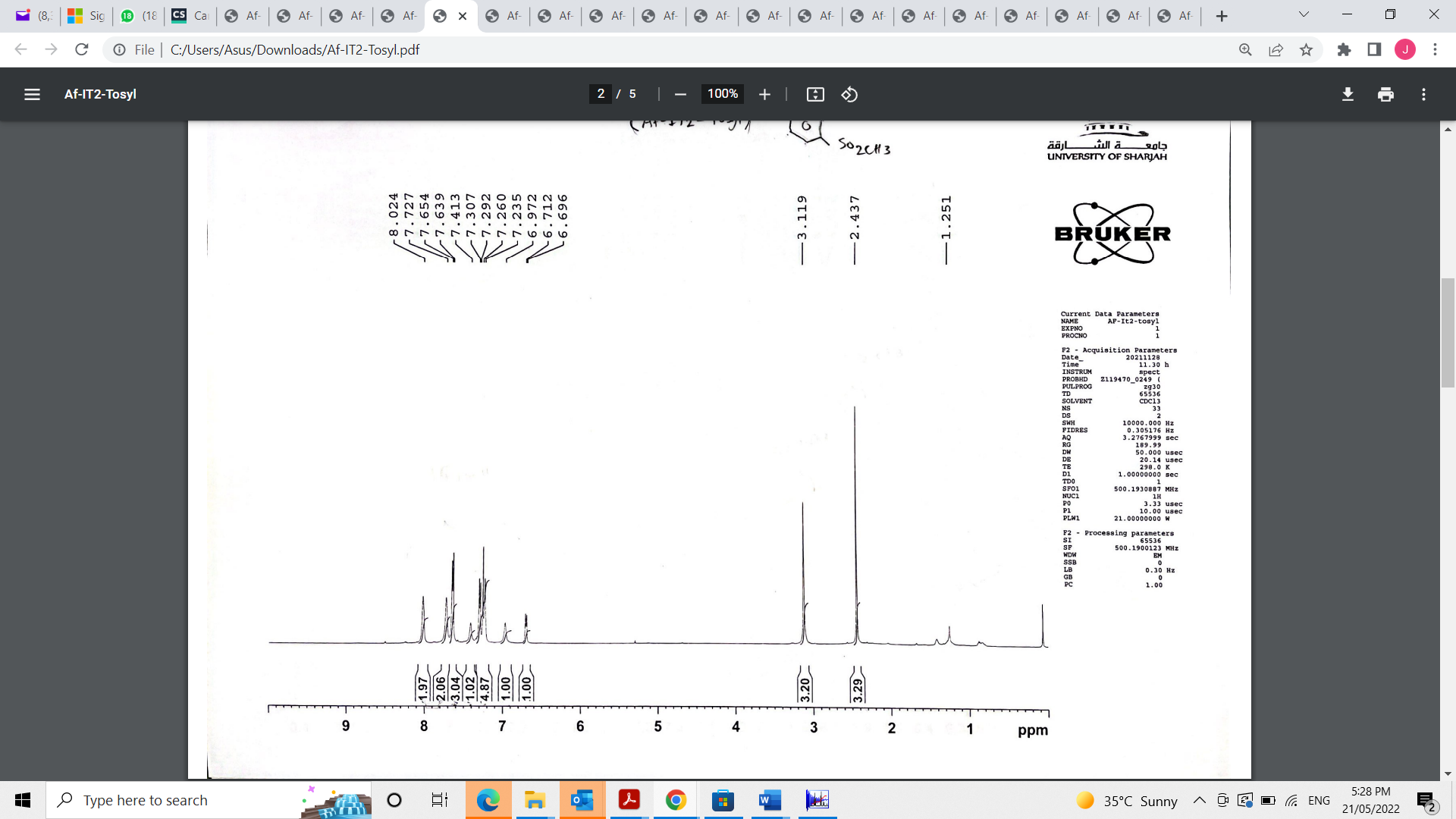


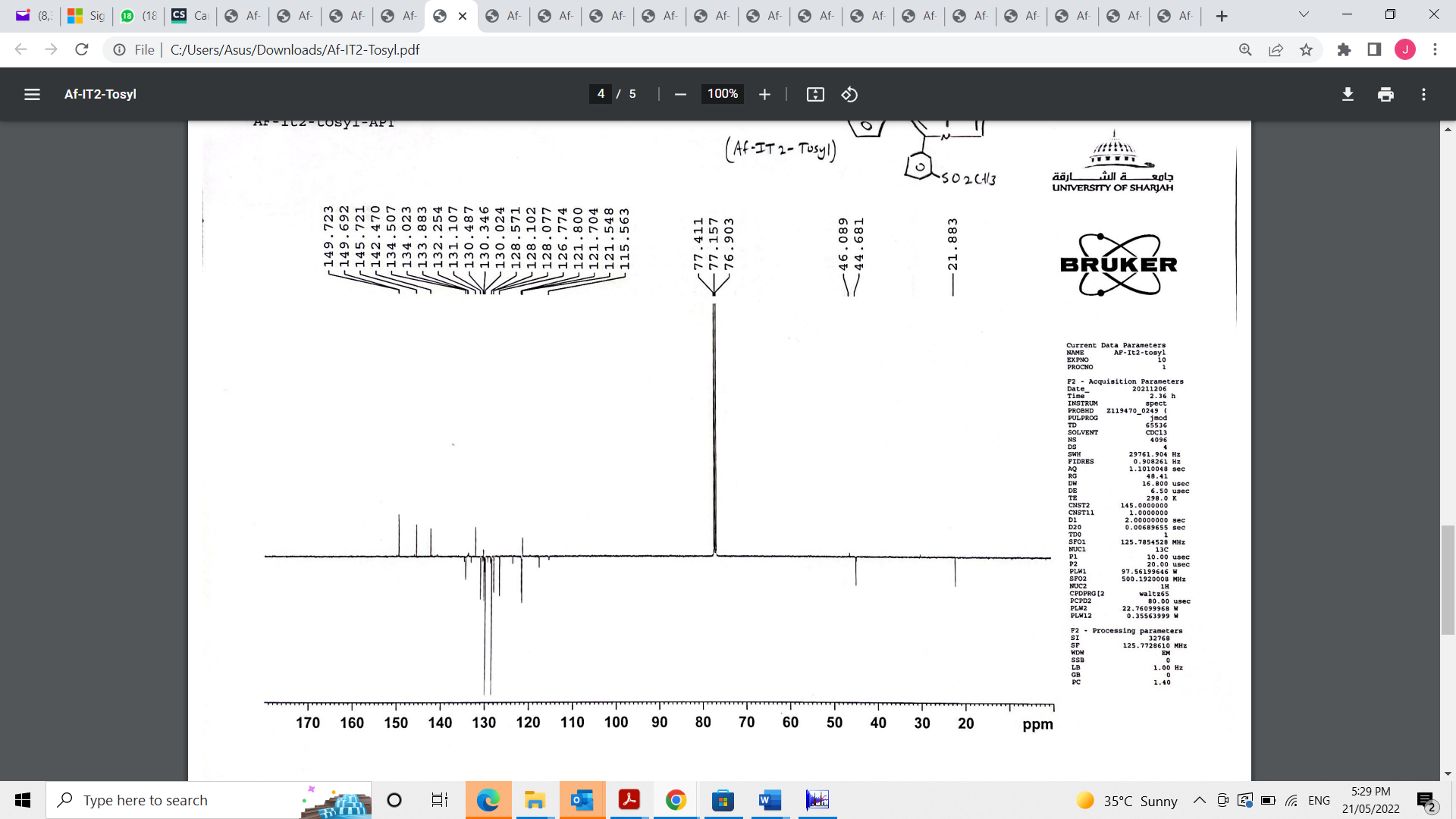


**Figure S34.** ^1^H NMR and ^13^C NMR charts of compound **2g**.

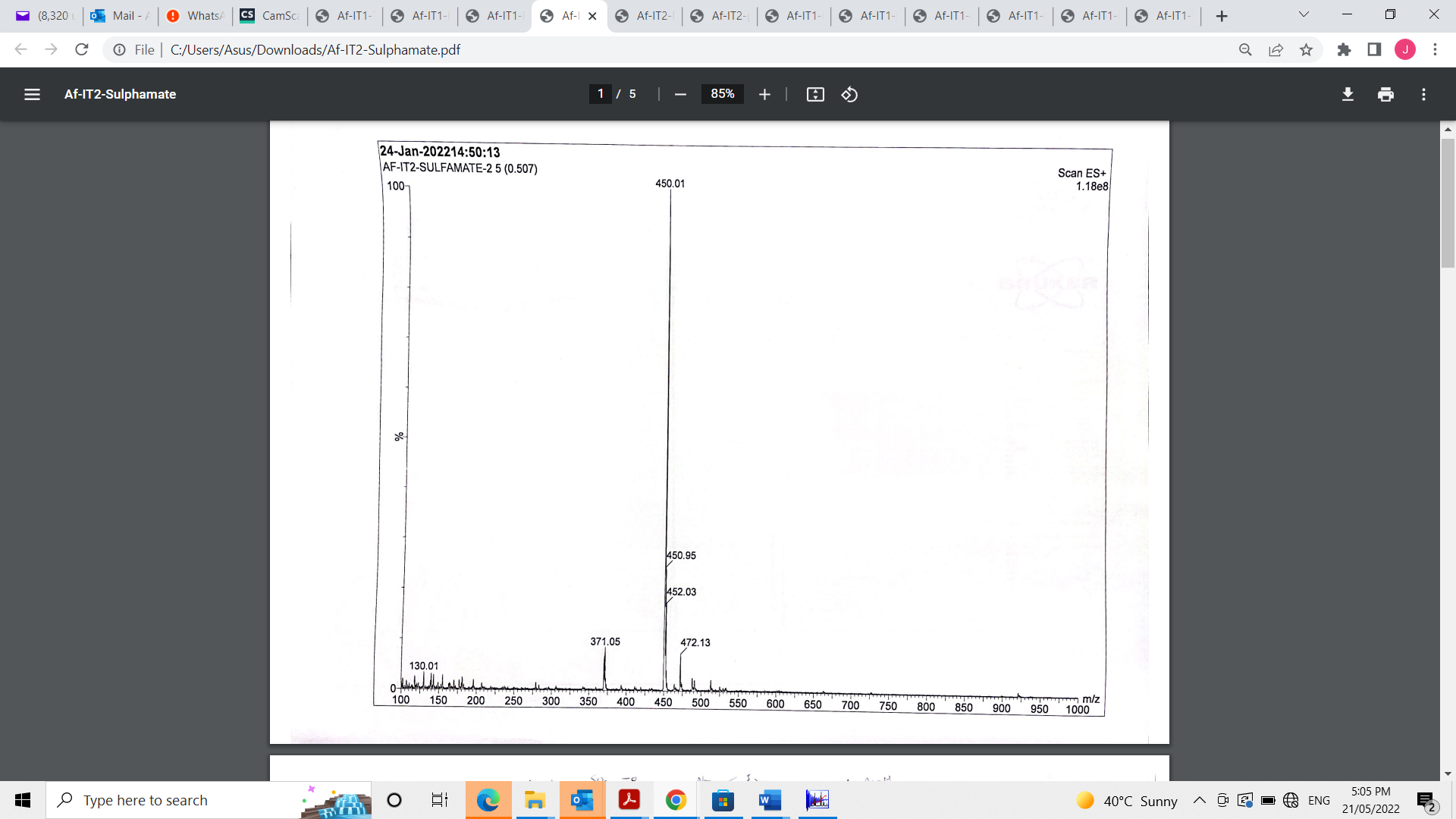


**Figure S35.** LC-MS chart of compound **2h**.


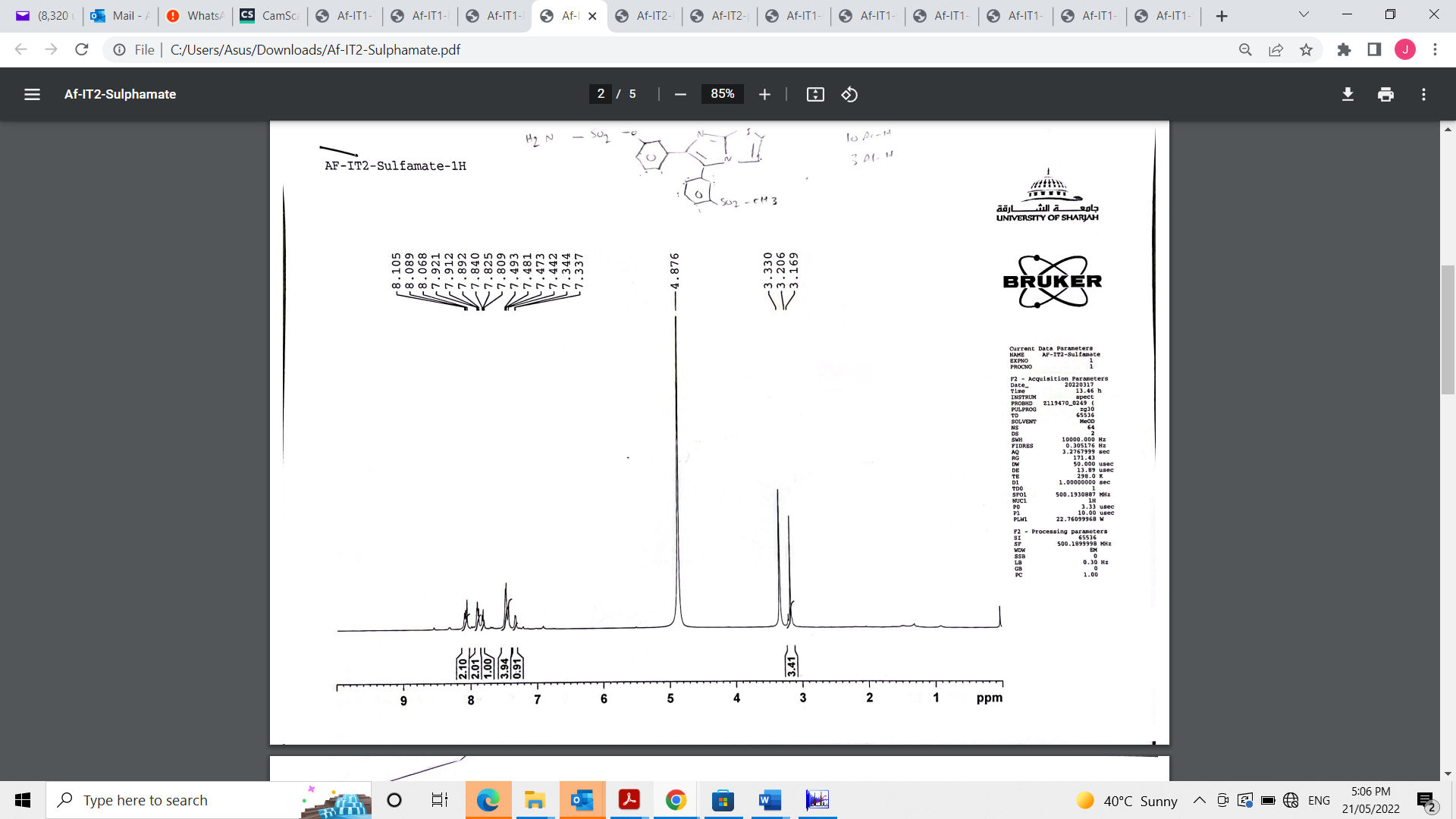


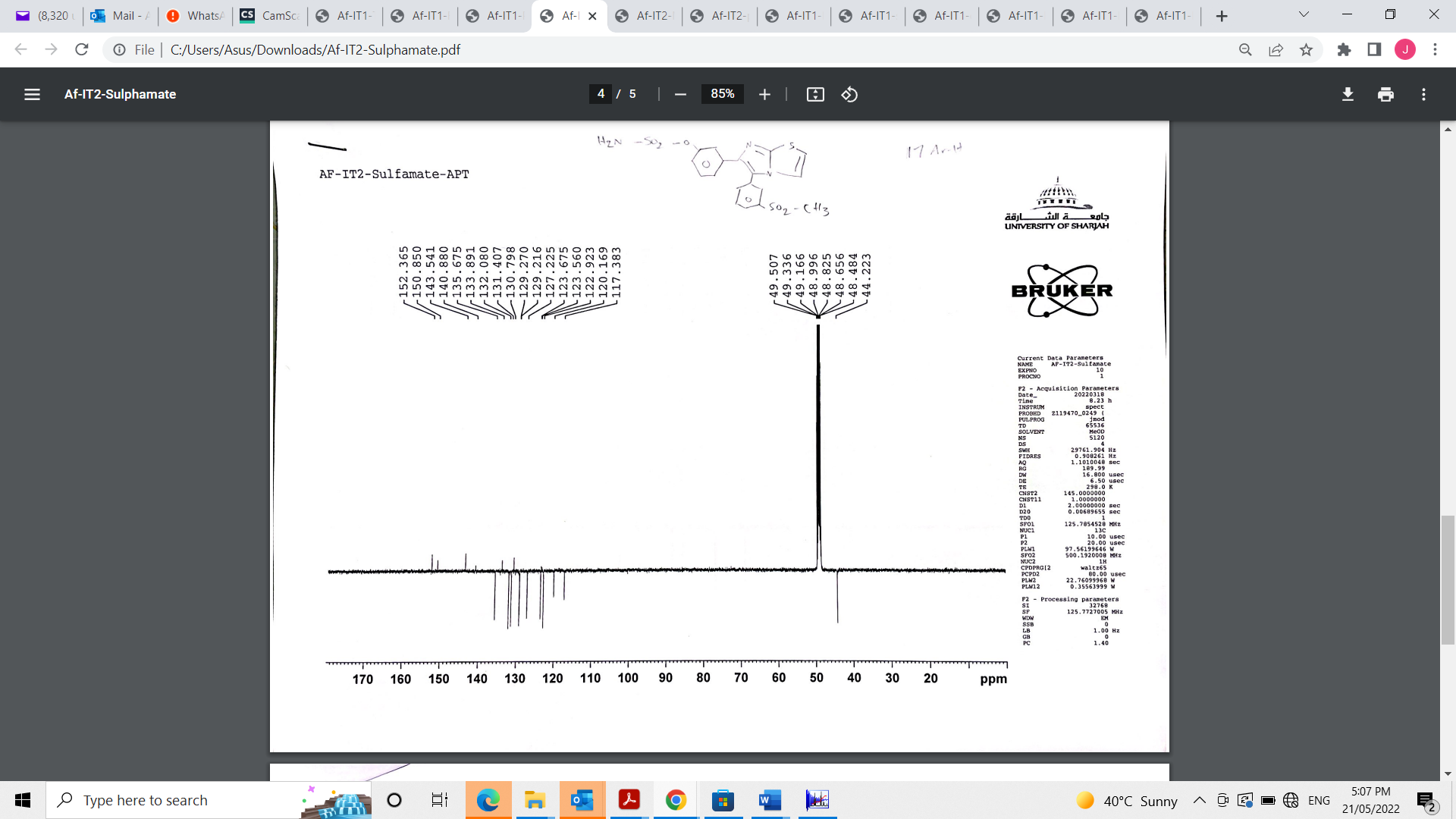


**Figure S36.** ^1^H NMR and ^13^C NMR charts of compound **2h**.

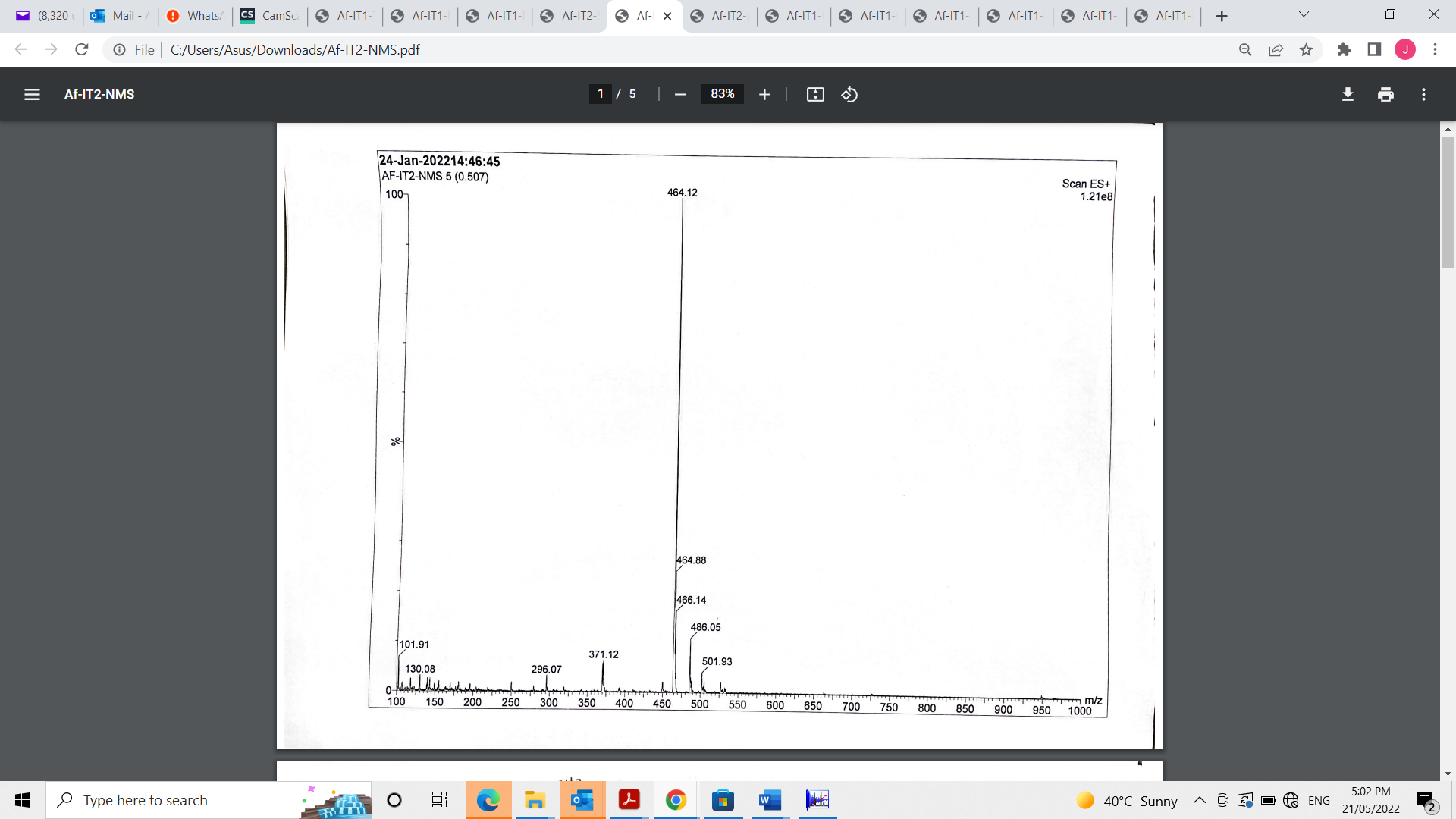


**Figure S37.** LC-MS chart of compound **2i**.


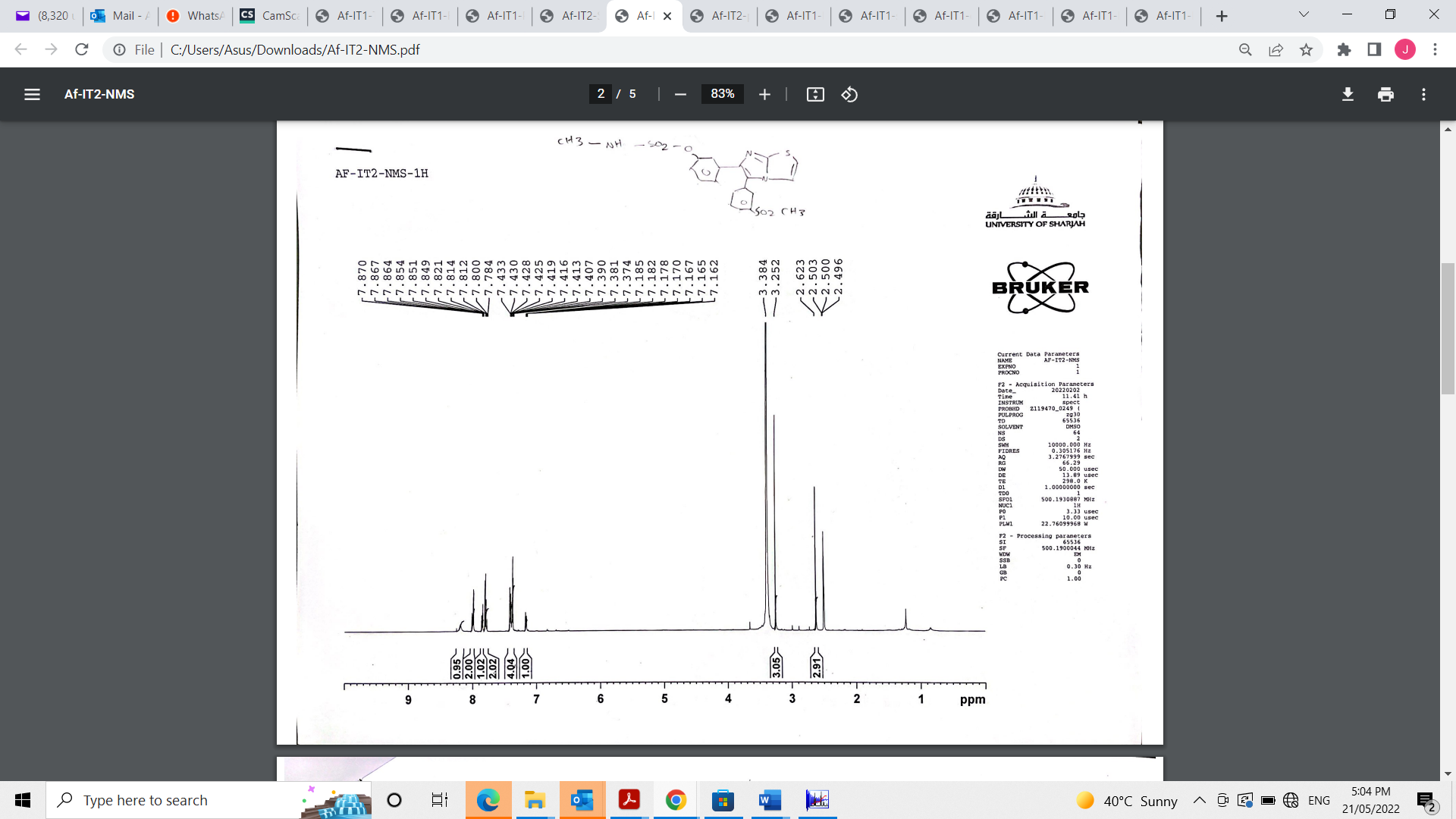


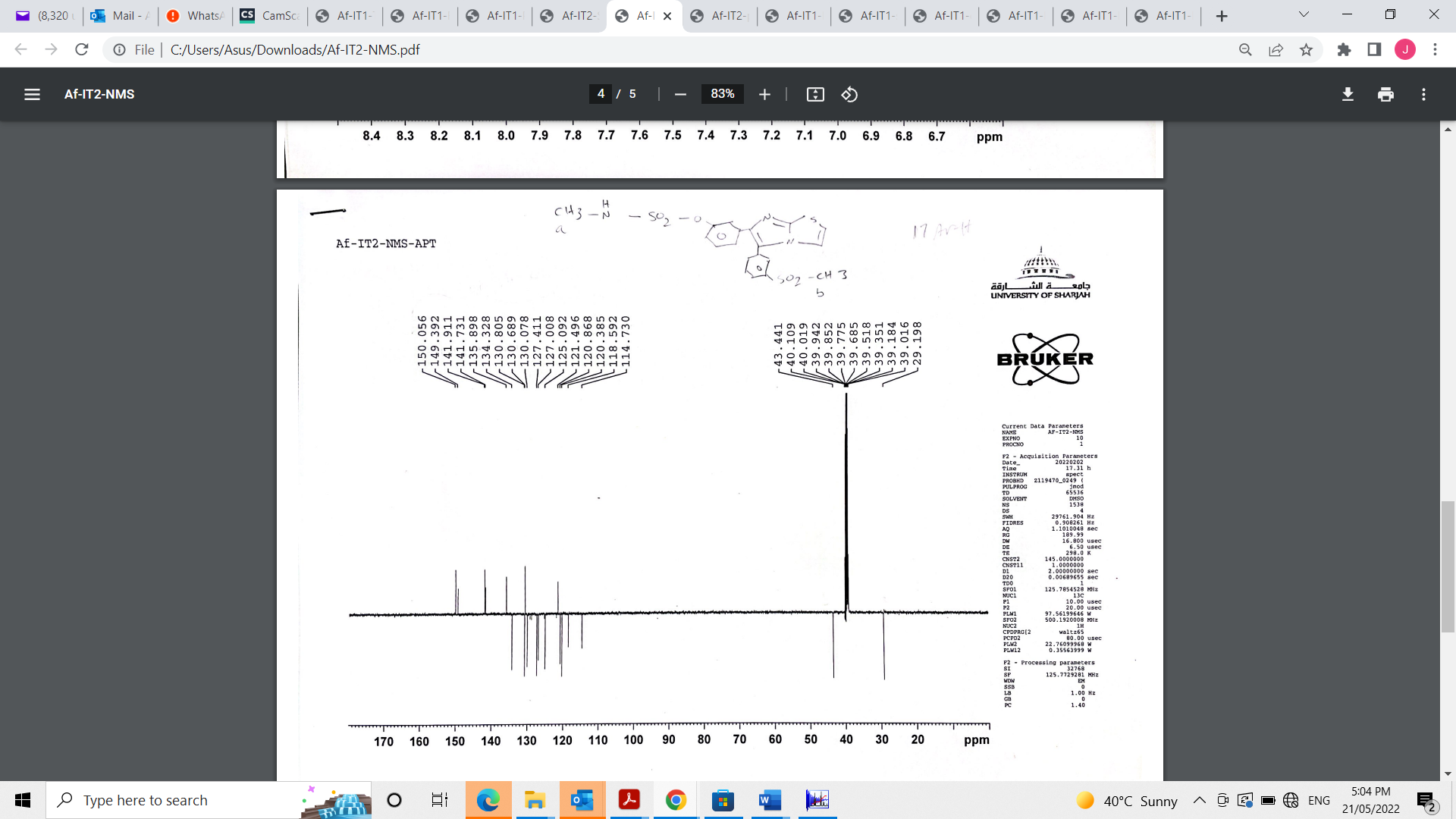


**Figure S38.** ^1^H NMR and ^13^C NMR charts of compound **2i**.

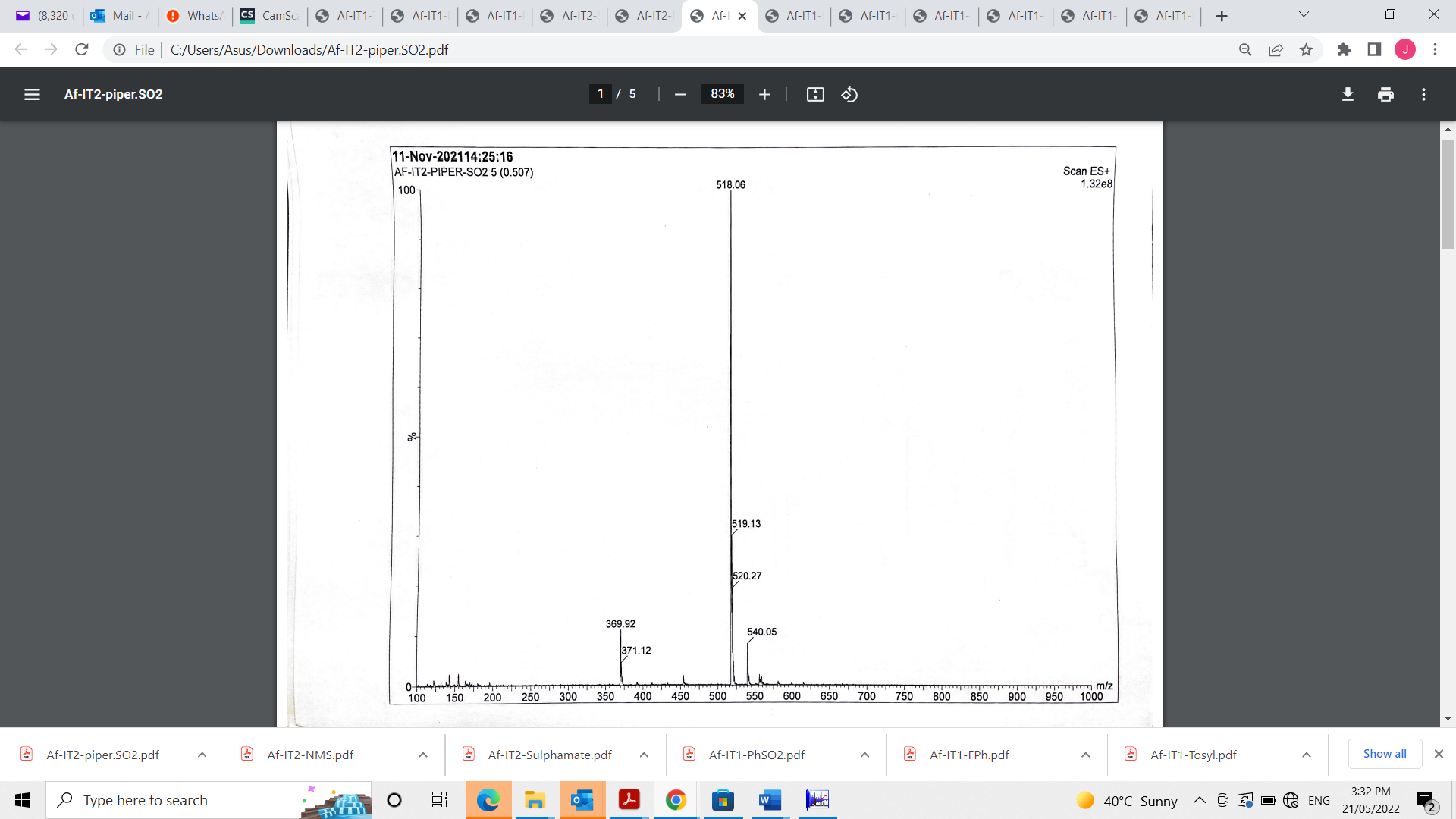


**Figure S39.** LC-MS chart of compound **2j**.


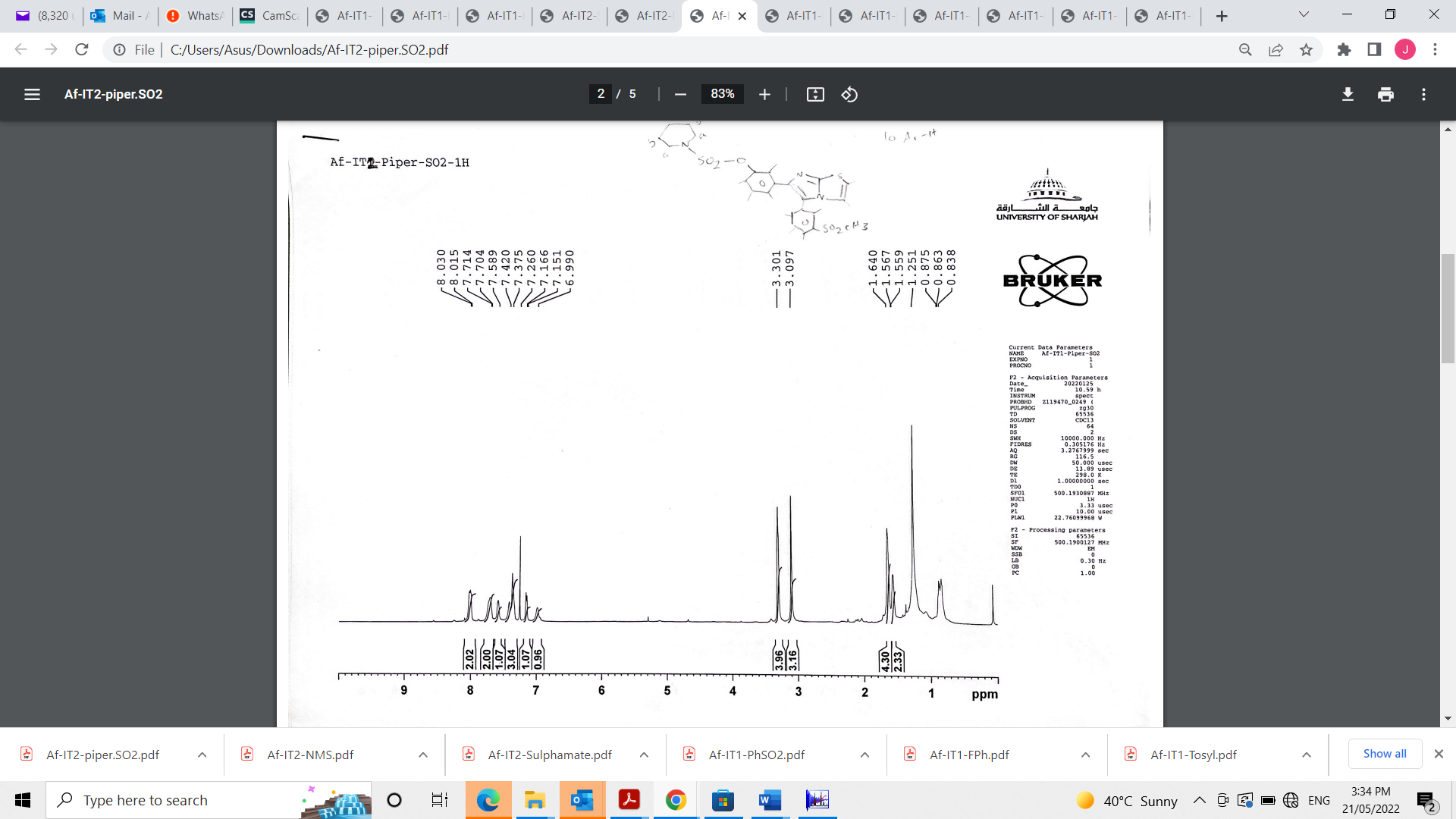


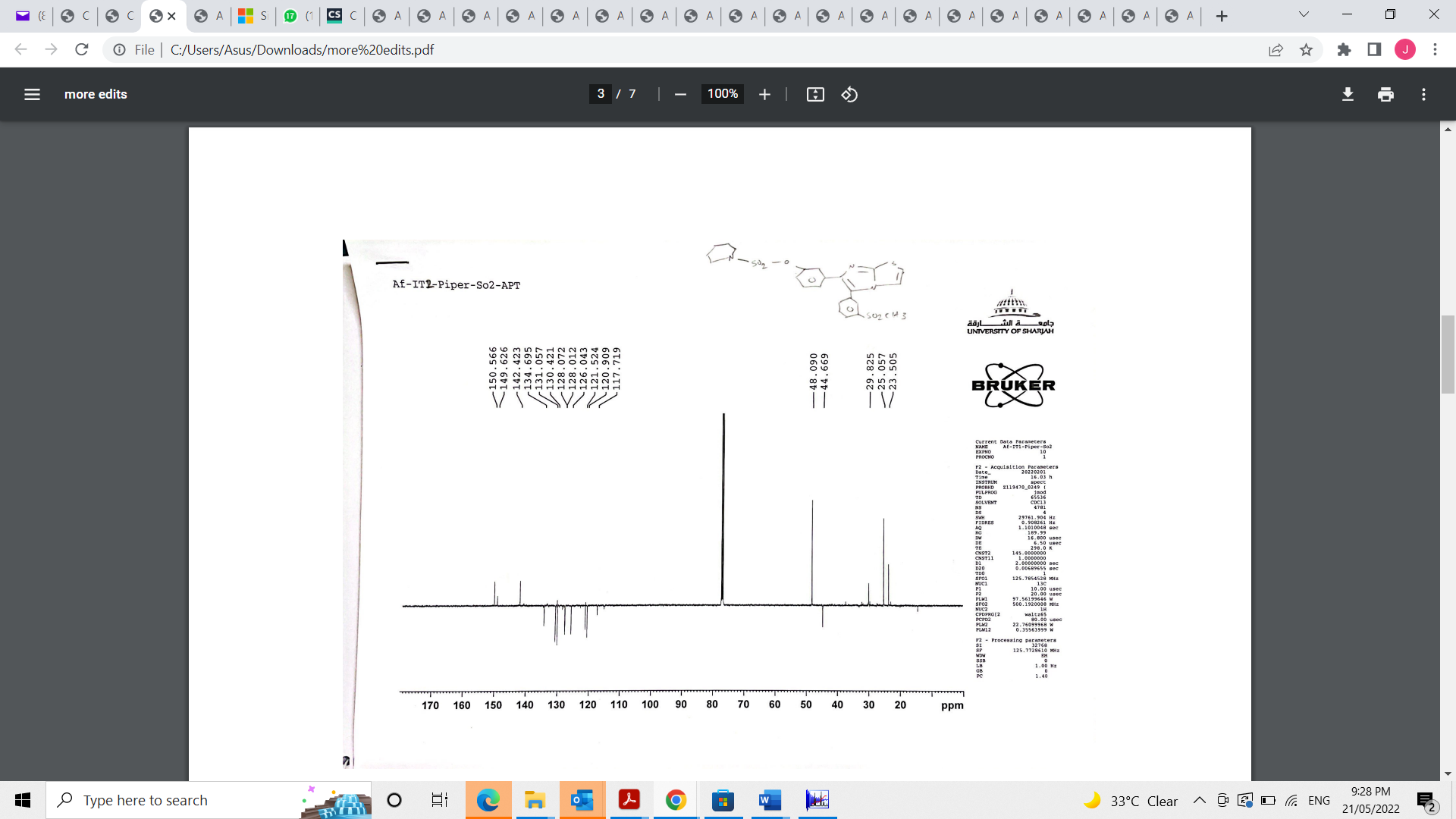


**Figure S40.** ^1^H NMR and ^13^C NMR charts of compound **2j**.

**Dose-response curves of compounds 1a-2j against urease enzyme**

| **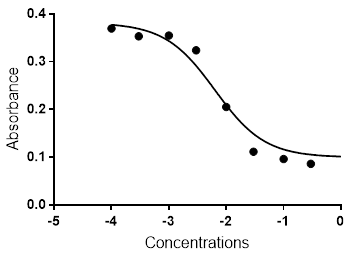**  **1a** | **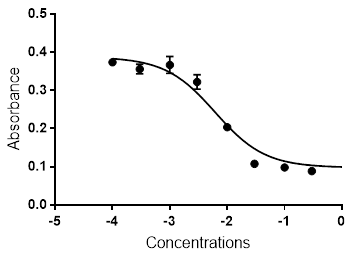1b** | **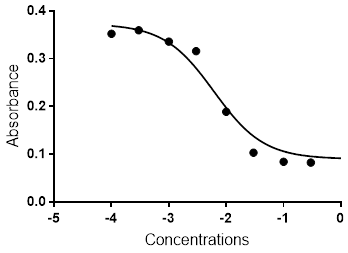**  **1c** |
| --- | --- | --- |
| **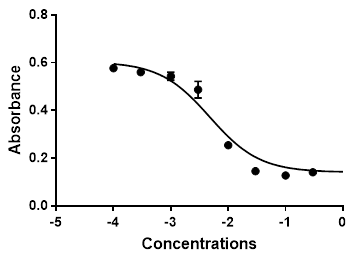**  **1d** | **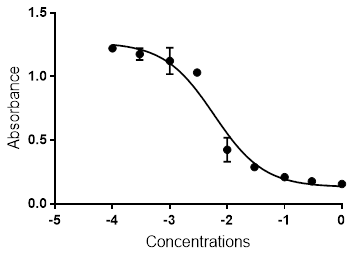**  **1e** | **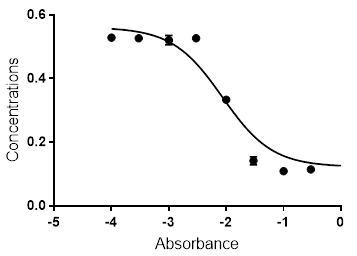**  **1f** |
| **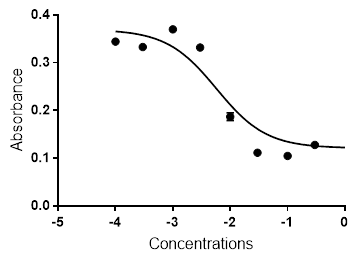**  **1g** | **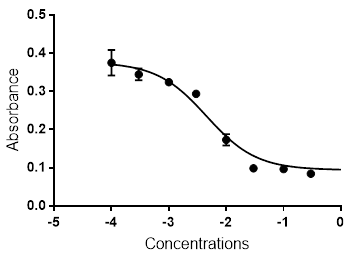**  **1h** | **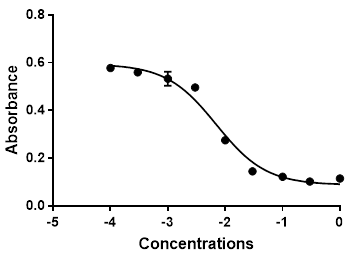**  **1i** |
| **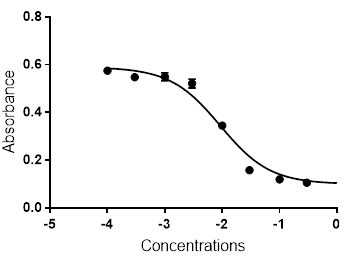**  **1j** | **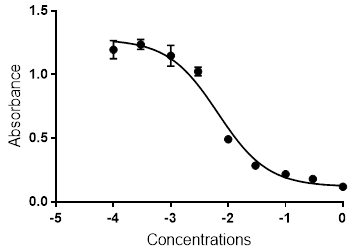2a** | **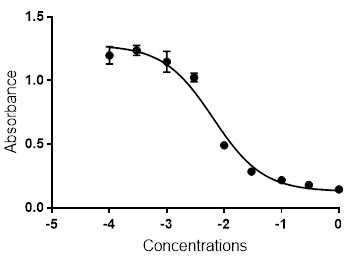2b** |
| **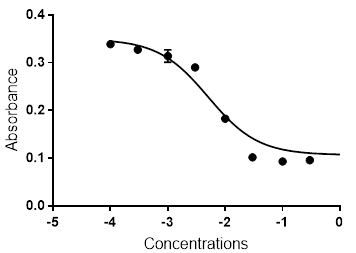**  **2c** | **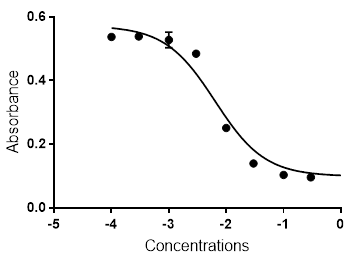**  **2d** | **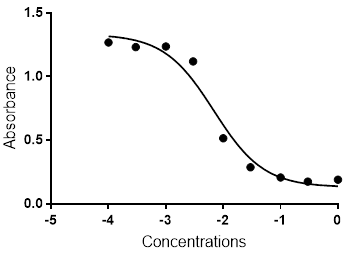**  **2e** |
| **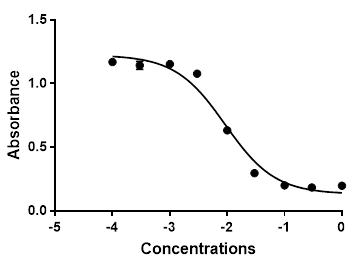2f** | **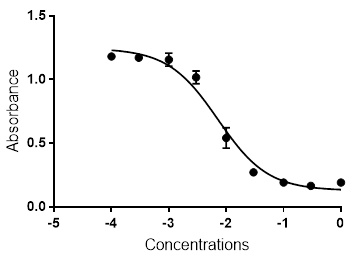**  **2g** | **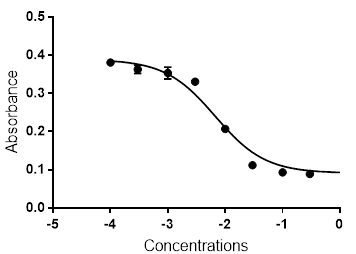**  **2h** |
| **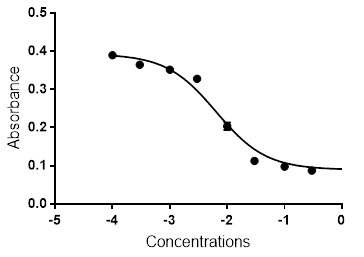2i** | **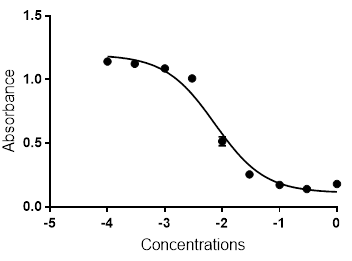**  **2j** | **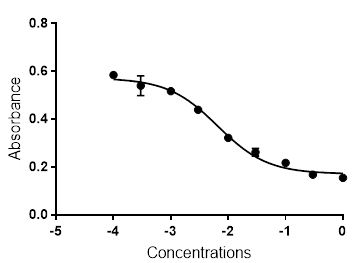**  **Acetohydroxamic acid** |

**Figure S41.** Dose-response curves of compounds **1a-2j** against urease enzyme.


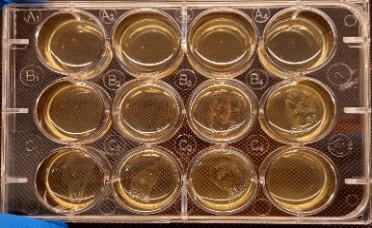

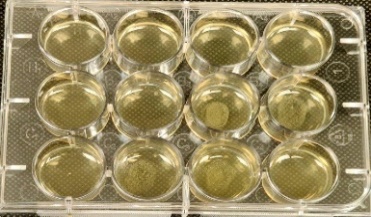

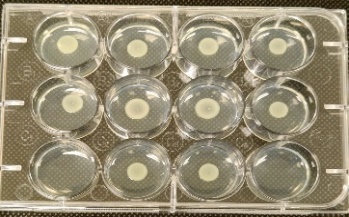

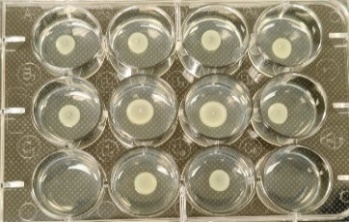

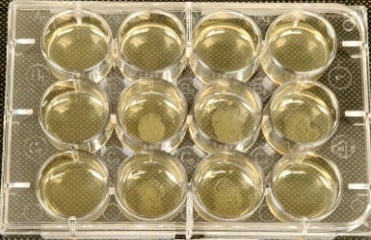

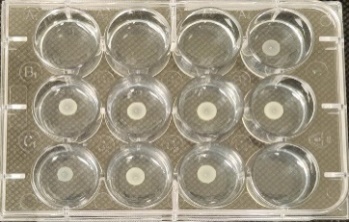

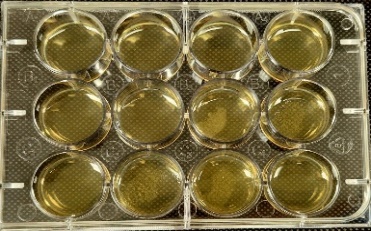

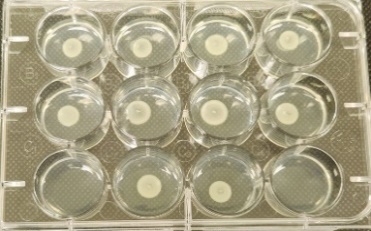

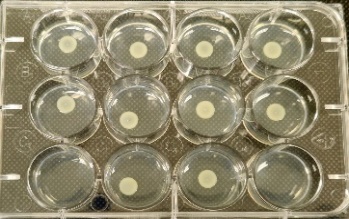

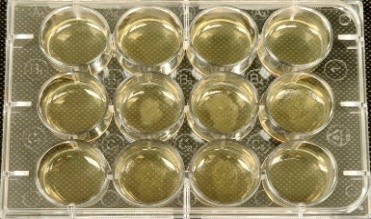

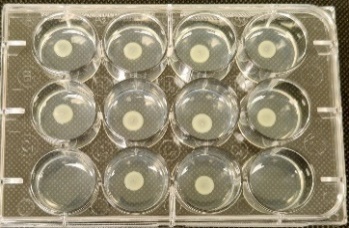

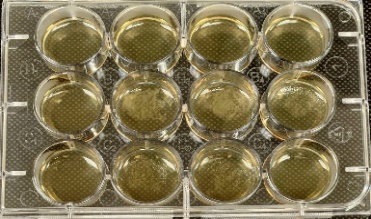


***H. pylori***

***E. coli***

***E. coli***

***H. pylori***

**[Compound 1a]**

**[Compound 1d]**

**[Compound 1h]**

**[Compound 2d]**

**[Compound2f]**

**[AHA]**

**Figure S42.** Minimum inhibitory concentration (MIC, mM) determination of the five most significant anti-urease imidazothiazole derivatives (Compound **1a**, **1d**, **1h**, **2d**, and **2f**) against *H. pylori* and *E. coli* using agar dilution method. Acetohydroxamic acid (AHA) was used as control anti-urease compound. A1-A4 and B1-B4 wells represents bacterial growth at 0.8 mM, 0.4 mM, 0.2 mM, 0.1 mM, 0.05 mM, 0.025 mM, and 0.006 mM of the five tested. Acetohydroxamic acid (AHA) was used as control anti-urease compound at the following concentrations: 100 mM, 50 mM, 25 mM, 12.5 mM, 6.25 mM, 3.12 mM, 1.56 mM, and 0.78 mM (A1-A4 and B1-B4). The antibiotic amoxicillin in C1 and C2 wells was used as positive control of the agar dilution method. C1 and C2 represent the bacterial growth with amoxicillin at 10 µg/mL and 0.007 µg/mL for *H. pylori* and 12 µg/mL and 3 µg/mL for *E. coli*. D1 represents the positive growth control of the bacteria without any anti-urease compounds. D2 represents the negative control media. The above represented MICs were extracted from one over two repeated experiments.

***H. pylori E. coli***


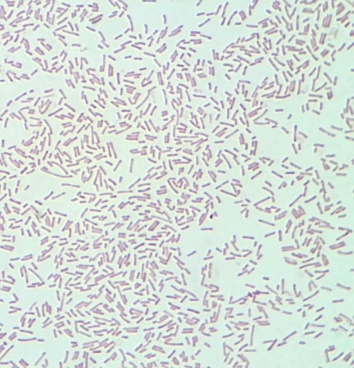

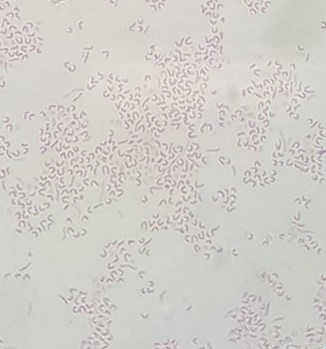


**Figure S43.** Gram stain micrograph confirming the rode curved shape of *Helicobacter pylori* and the rode shape of *Escherichia coli***.**


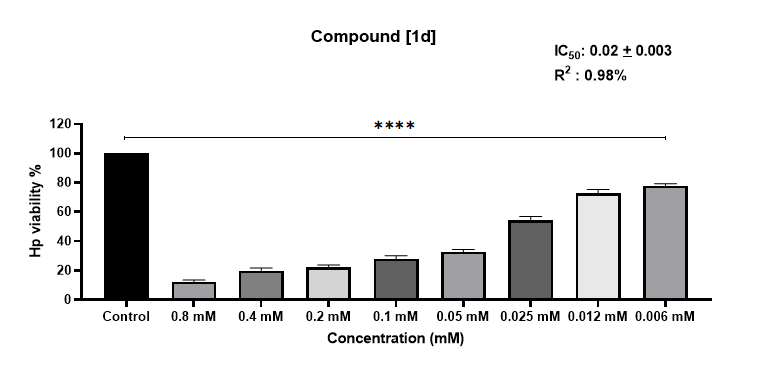

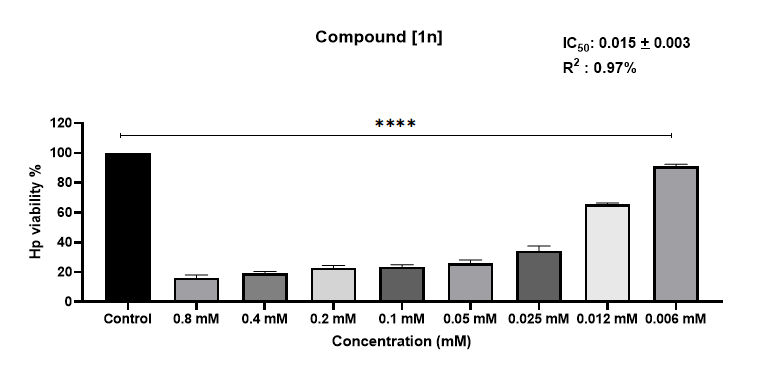

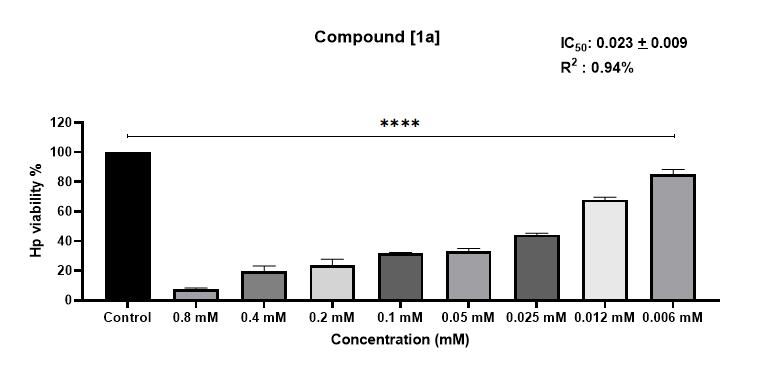

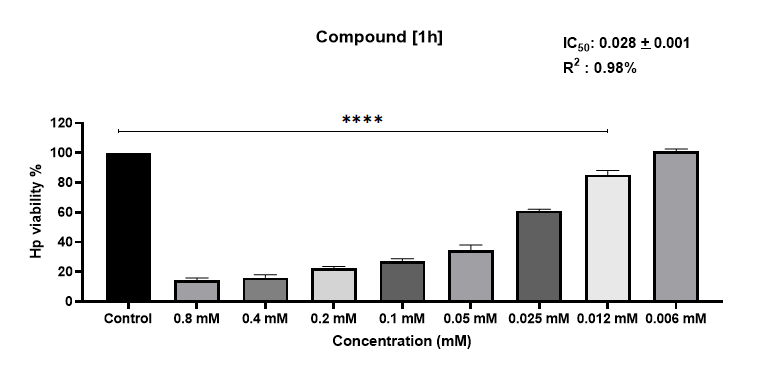

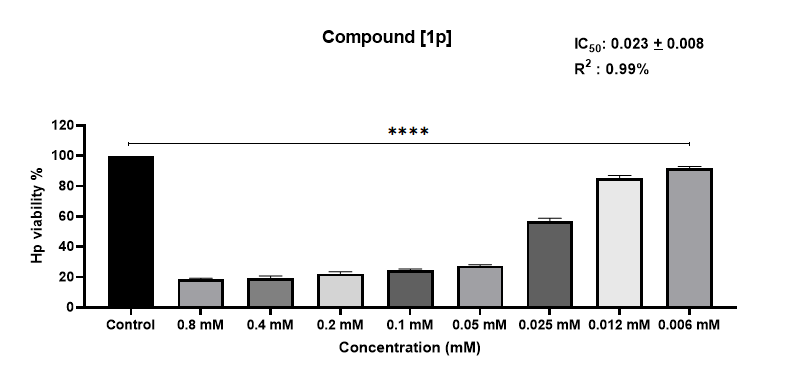

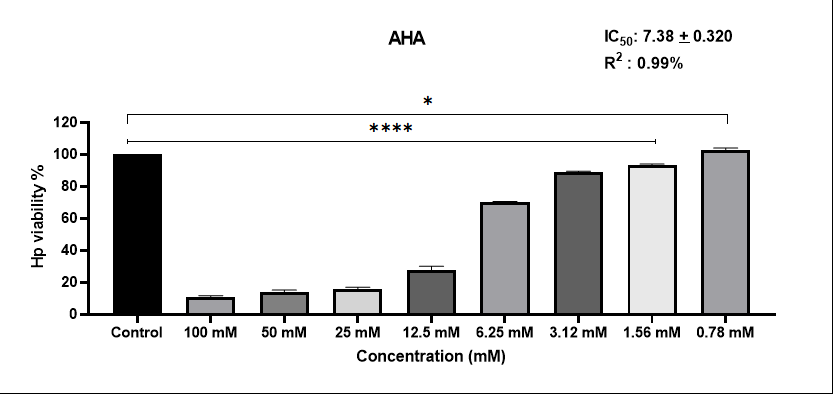


**Compound [1a]**

**Compound [2d]**

**Compound [1d]**

**Compound [2f]**

**Compound [1h]**

**[AHA]**

**Figure S44.** IC_50_ (mM) determination of the five most significant anti-urease imidazothiazole derivatives (Compound **1a**, **1d**, **1h**, **2d**, and **2f**) against *H. pylori* using micro broth dilution method. Acetohydroxamic acid (AHA) was used as control anti-urease compound. The experiments were repeated twice and results were represented as ± SEM. Asterisks above the line indicate differences between adjacent groups * p < 0.05, **** p < 0.0001.


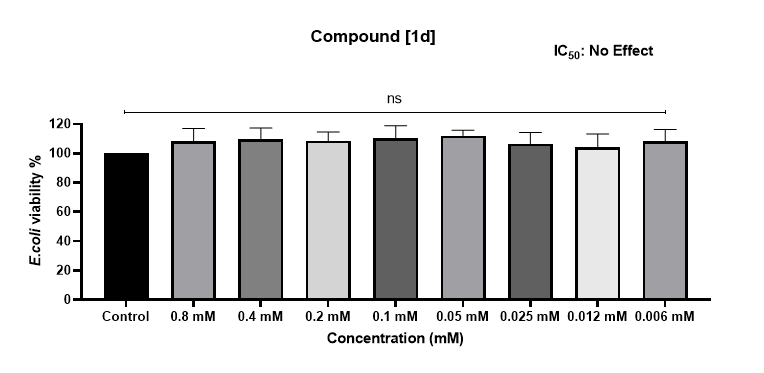


**Compound [1a]**

**Compound [2d]**

**Compound [1d]**

**Compound [2f]**

**Compound [1h]**

**[AHA]**

**Figure S45.** IC_50_ (mM) determination of the five most significant anti-urease imidazothiazole derivatives (Compound **1a**, **1d**, **1h**, **2d**, and **2f**) against *E. coli* using micro broth dilution method. Acetohydroxamic acid (AHA) was used as control anti-urease compound. The experiments were repeated twice and results were represented as ± SEM. Asterisks above the line indicate differences between adjacent groups * p < 0.05, ** p < 0.001, **** p < 0.0001.

**Compound [1a]**

**Compound [2d]**

**Compound [1d]**

**Compound [2f]**

**Compound [1h]**

**[AHA]**

**Figure S46.** Cytotoxicity evaluation (IC_50_) of the five most significant anti-urease imidazothiazole derivatives (Compound **1a**, **1d**, **1h**, **2d**, and **2f**) against gastric cell line (AGS) using MTT assay. Acetohydroxamic acid (AHA) was used as control anti-urease compound. The experiments were repeated twice and results were represented as ± SEM. Asterisks above the line indicate differences between adjacent groups * p < 0.05, ** p < 0.001, **** p < 0.0001. The cell viability is presented from two repeated experiment as ± SE.
