# Supplementary figures and images for: Design and synthesis of novel anti-urease imidazothiazole derivatives with promising antibacterial activity against Helicobacter pylori
Source: PLoS One. 2023 Jun 2;18(6):e0286684. doi: 10.1371/journal.pone.0286684 (PMC10237672; doi:10.1371/journal.pone.0286684)

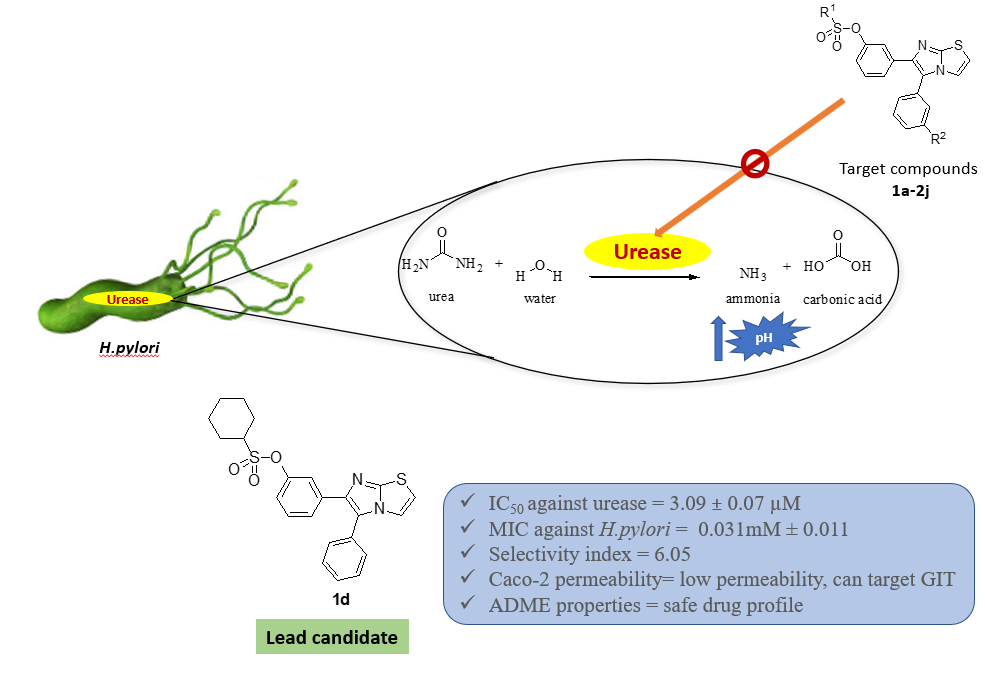

Supplement: S1 Graphical abstract — (DOCX) [file pone.0286684.s002.docx]
